# Structure/affinity studies in the bicyclo-DNA series: Synthesis and properties of oligonucleotides containing bc^en^-T and iso-tricyclo-T nucleosides

**DOI:** 10.3762/bjoc.10.194

**Published:** 2014-08-12

**Authors:** Branislav Dugovic, Michael Wagner, Christian J Leumann

**Affiliations:** 1 Department of Chemistry and Biochemistry, University of Bern, Freiestrasse 3, CH-3012 Bern, Switzerland

**Keywords:** DNA/RNA affinity, nucleic acids, nucleosides, oligonucleotides, oligonucleotide therapy, X-ray structures

## Abstract

We present the synthesis of the two novel nucleosides iso-tc-T and bc^en^-T, belonging to the bicyclo-/tricyclo-DNA molecular platform. In both modifications the torsion around C6’–C7’ within the carbocyclic ring is planarized by either the presence of a C6’–C7’ double bond or a cyclopropane ring. Structural analysis of these two nucleosides by X-ray analysis reveals a clear preference of torsion angle γ for the gauche orientation with the furanose ring in a near perfect 2’-endo conformation. Both modifications were incorporated into oligodeoxynucleotides and their thermal melting behavior with DNA and RNA as complements was assessed. We found that the iso-tc-T modification was significantly more destabilizing in duplex formation compared to the bc^en^-T modification. In addition, duplexes with complementary RNA were less stable as compared to duplexes with DNA as complement. A structure/affinity analysis, including the already known bc-T and tc-T modifications, does not lead to a clear correlation of the orientation of torsion angle γ with DNA or RNA affinity. There is, however, some correlation between furanose conformation (N- or S-type) and affinity in the sense that a preference for a 3’-endo like conformation is associated with a preference for RNA as complement. As a general rule it appears that *T*_m_ data of single modifications with nucleosides of the bicyclo-/tricyclo-DNA platform within deoxyoligonucleotides are not predictive for the stability of fully modified oligonucleotides.

## Introduction

Antisense oligonucleotides (ASOs) can interfere with gene expression via various biological mechanisms, depending on the nature of the cellular RNA target [1]. First and foremost they can inhibit translation by targeting a mature mRNA in either its coding or non-coding part of the sequence by a steric block or an RNase H dependent degradation mechanism. Furthermore, it has recently been shown that ASOs can alter RNA splicing when targeting exon/intron junctions or splice enhancer or silencer binding sites on pre-mRNAs, thus leading to alternative splicing [2–3], to exon skipping [4–5] or to exon inclusion [6]. In addition they can restore the function of mRNAs containing extended aberrant repeat sequences in their non-coding region by either restoring correct cellular localization or inhibiting vital protein sequestration by the aberrant repeats [7]. Last but not least, there is an ever growing number of micro RNAs (miRNAs) that are involved in genetic and epigenetic regulation of gene expression. Their misregulation stays at the onset of various forms of cancer and other metabolic diseases, and targeting of such miRNAs with ASOs (antimirs or anatagomirs) has been shown in the recent past to be a promising therapeutic principle [8].

**Figure 1:**
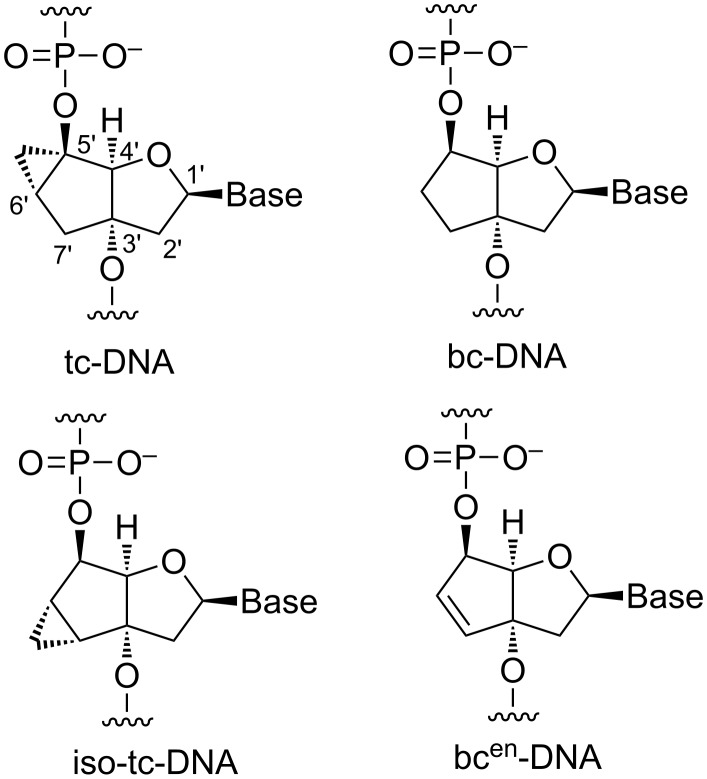
Chemical structures and carbon numbering scheme of tricyclo(tc)-DNA (top, left), bicyclo(bc)-DNA (top, right) and the newly synthesized iso-tricyclo(iso-tc)-DNA (bottom, left) and bicyclo-en(bc^en^)-DNA (bottom, right).

There exists a multitude of chemical modifications in ASOs. Historically, the first modification was the replacement of the phosphodiester linking units in DNA by phosphorothioate groups, thus conferring higher metabolic stability to ASOs in plasma and tissue [9–10]. Another site of modification is the 2’-OH group of RNA that can be equipped or replaced with various chemical entities typically aiming at higher affinities to the corresponding RNA targets [11–14]. More diverse analogues include structures in which the sugar phosphate backbone has been replaced by a charge neutral peptide backbone, such as the peptide nucleic acids (PNAs) [15] or by a nucleotide derived phosphorodiamidate backbone, such as the morpholino oligonucleotides (PMOs) [16]. Of particular interest is the class of conformationally constrained oligonucleotides. Members of this class are amongst others the locked nucleic acids (LNA) [17–18], the hexose nucleic acids (HNAs) [19] and the family of bi- and tricyclo-DNA (Figure 1) [20–23]. These analogues aim at increasing RNA affinity by structurally preorganizing single strands for duplex formation.

Bc- and tc-DNA have been conceived to reduce the entropy upon duplex formation with a nucleic acid target by reducing the conformational flexibility around the C3’–C4’ and C4’–C5’ bonds, while achieving as much as possible of a geometric match with the backbone conformation of DNA in duplexed form. From this a gain in the free energy of duplex formation and, hence, more stable duplexes are expected [24]. Over the years we became interested in determining the structure/RNA affinity relationship of the underlying sugar scaffold and to develop them into a molecular platform for oligonucleotide therapeutics. Given the exclusiveness of the ethylene bridge between the centers 3’ and 5’ with respect to DNA or RNA we have identified this structural element to be the primary goal for chemical modification [25–30]. In continuation of this work we decided to investigate on two novel thymine nucleosides with restricted conformation of the C6’–C7’ bond, namely bc^en^-T and iso-tc-T (Figure 1). Here we present the synthesis and X-ray structural characterization of the respective nucleosides, their incorporation into oligodeoxynucleotides by phosphoramidite chemistry as well as the DNA and RNA affinity profiles of the modified oligonucleotides.

## Results

### Synthesis of building blocks

**Scheme 1:**
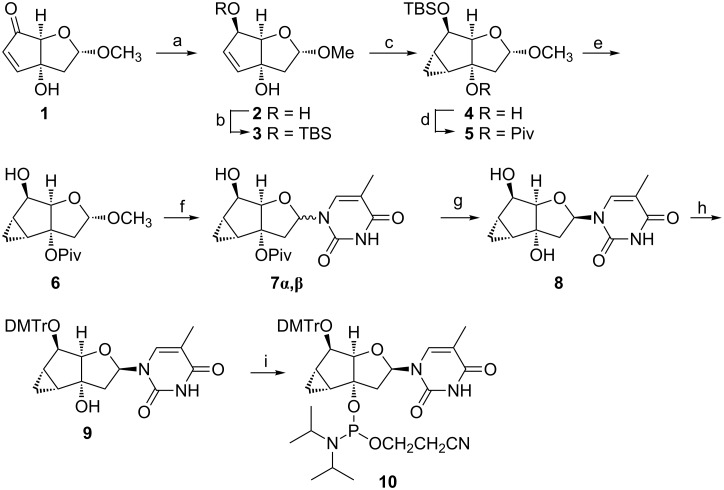
Conditions: (a) NaBH_4_, CeCl_3_·7H_2_O, MeOH, −78 °C → rt, 1.5 h, 73% (+9% of C6-epimer); (b) TBS-Cl, imidazole, CH_2_Cl_2_, rt, 16 h, 79%; (c) Et_2_Zn in hexane (1 M), CH_2_I_2_, CH_2_Cl_2_, 0 °C → rt, 16 h, 86%; (d) PivCl, DMAP, pyridine, ClH_2_C–CH_2_Cl, 70 °C, quant.; (e) TBAF, THF, rt, 19 h, 95%; (f) thymine, BSA, SnCl_4_, CH_3_CN, 0 °C → rt, 17 h, 56% **7β** + 22% (**7α/β** 2.5:1); (g) Bu_4_NOH, H_2_O/dioxane, rt, 16 h, 94%; (h) DMTrCl, pyridine, CH_2_Cl_2_, rt, 24 h, 98%; (i) CEP-Cl, DIPEA, THF, rt, 4 h, 89%.

The synthesis of the phosphoramidite **10** started with the bicyclic intermediate **1** that had previously been described on the way to related bicyclo-DNA derivatives (Scheme 1) [30]. Following an obvious synthetic strategy, compound **1** was subjected to carbonyl reduction which occurred with high stereoselectivity from the less hindered, convex side of the bicyclic system, resulting in alcohol **2** along with traces of its epimer. To increase the stereoselectivity of the upcoming cyclopropanation reaction it seemed appropriate to protect the secondary hydroxy group as TBS ether (→ **3**). Indeed cyclopropanation of **3** with diethylzinc and CH_2_I_2_ proceeded stereospecifically, again from the convex side of the bicyclic system, to give **4**. Subsequent nucleosidation of **4** via the Vorbrüggen procedure [31–32] with transient protection of the tertiary hydroxy group in **4** as TMS ether, however, was unsuccessful and yielded only the corresponding α-nucleoside in yields below 25%. We reasoned that the exclusive formation of α-nucleosides is due to the steric bulk of the TBS group, further suppressing the intrinsically disfavored β-(endo)-face attack of the base. To counterbalance these effects we chose to protect the tertiary hydroxy group as a pivaloyl ester thus increasing the steric bulk on the α-face, and relieving that on the β-face by replacing the TBS by a transient TMS group. The conversion of **4** → **6** proceeded smoothly and indeed, the use of compound **6** as nucleobase acceptor improved the yield of nucleosides **7α**,**β** in general and led to an acceptable β:α = 2.5:1 ratio of anomers. Subsequent saponification of **7α**,**β** (unseparable by flash chromatography) proved to be tricky and after testing a series of standard techniques, only treatment with Bu_4_NOH in a mixed organic/aqueous solvent gave nucleosides **8α**,**β** in good yield. It was at this step where the two anomers could be readily separated by flash chromatography. Continuing with **8β** the synthesis of **10** was concluded by standard tritylation (→ **9**) and phosphitylation.

**Scheme 2:**
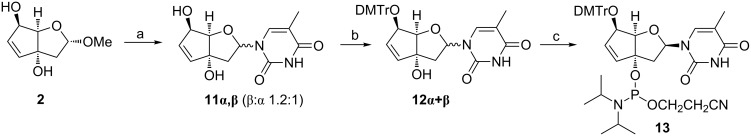
Conditions: (a) thymine, BSA, TMSOTf, TMSCl, CH_3_CN, rt, 2.5 h; (b) DMTrCl, pyridine, rt, 16 h, 29% of **12α** and 34% of **12β** (over two steps); (c) CEP-Cl, DIPEA, THF, rt, 1 h, 94%.

To extend on the structure/nucleic acid affinity profile of this modification we also became interested in nucleoside **11β**, containing a double bond instead of the cyclopropane ring. In the context of oligonucleotides this derivative seemed appropriate to investigate the direct steric influence of the cyclopropyl/methylene group in a bicyclic sugar scaffold that is otherwise very similar in flexibility and geometry. The corresponding building block **13** (Scheme 2) was easily available via nucleosidation of sugar intermediate **2** (in situ TMS protection of both hydroxy groups) leading to the mixture of anomeric nucleosides **11α**,**β** in a ratio of β:α = 1.2:1. After standard tritylation of **11α**,**β** the anomeric mixture **12α**,**β** became separable by flash chromatography and the corresponding β-nucleoside **12β** could be smoothly converted into the phosphoramidite **13** by standard methods.

### Structural properties of nucleosides

**Figure 2:**
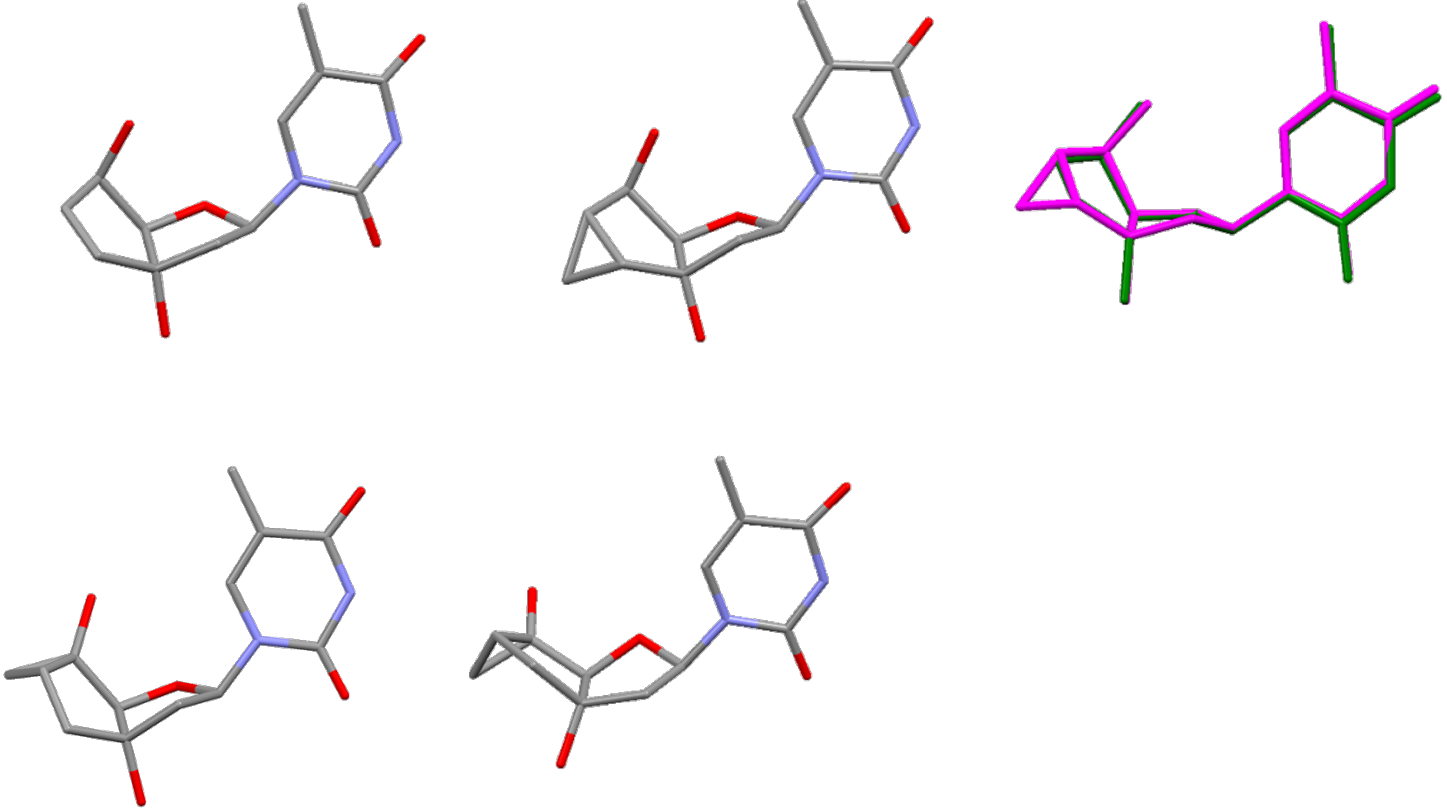
X-ray structure of top row: nucleosides **8β** (left), **11β** (center) and overlay of both structures (right); bottom row: tc-T (Mol A, left, Mol B, right).

To get an independent proof on the relative configuration around the cyclopropane ring and the glycosidic bond and to obtain insight into the conformational properties of the central bicyclic sugar scaffold, crystals of **8β** and **11β** were grown and subjected to X-ray analysis (Figure 2, Table 1). It clearly emerges that in both nucleosides the furanose unit appears in an almost perfect 2’-endo conformation giving rise to a *trans* arrangement of torsion angle δ (O3’–C3’–C4’–C5’). In both structures the cyclopentane ring exists in a shallow envelope conformation with C5’ being slightly out of plane. This leads to a *gauche* orientation of torsion angle γ (C3’–C4’–C5’–O5’). In both structures the base thymine is, as expected, in the *anti*-orientation. The overlay of both structures clearly highlights the similarity of both structures, indicating that the extra methylene group in **8β** plays no direct role in controlling the conformation of the bicyclic ring system.

**Table 1:** Selected backbone torsion angles and sugar pucker data for **8β** and **11β** and related bi/tricyclo-nucleosides from X-ray structures.

	γ	δ	χ	*P*	*ν*_max_

**8β**	86.8°	150.1°	−106.4°	167.8°	36.1
**11β**	86.9°	146.0°	−115.7°	160.7°	36.3
bc-T^a^	149.3°	126.5°	−112.7°	128.4°	42.4
tc-T^b^ Mol A	125.0°	152.5°	−130.4°	172.2°	36.7
tc-T^b^ Mol B	154.8°	98.7°	−120.3°	94.4°	36.0

^a^Ref [33]; ^b^two structurally independent molecules per asymmetric unit.

A comparison of **8β** and **11β** with bc-T, having a saturated cyclopentane unit clearly reveals structural differences. The largest deviation is associated with the position of the 5’-OH group which is in a pseudoequatorial orientation in bc-T, giving rise to a torsion angle γ in the anticlinal range. The saturation of the carbocyclic ring translates to a lesser extent also into the furanose ring where a 1’-exo instead of a 2’-endo conformation is observed in bc-T. Both furanose conformations, however, belong to the *S*-type and are thus structurally related in the context of nucleic acid duplex conformation. Quite interestingly, the original tc-T nucleoside [34], for which we solved the X-ray structure here for the first time (Figure 2), shows considerable conformational variability in the furanose part. The asymmetric unit contains two independent molecules (Mol A and Mol B) of which the furanose part in Mol A adopts a 2’-endo (S-type) conformation, while in Mol B a 4’-endo (N-type) conformation is observed. In summary it appears that rigidifying and planarizing the C5’–C6’-bond as in tc-nucleosides leads to an anticlinal orientation of torsion angle γ and variability between S- and N-type in the furanose conformation, whereas rigidifying and planarizing the C6’–C7’-bond, as in **8β** and **11β**, leads to a synclinal torsion angle γ and a consistent 2’-endo furanose conformation. Saturation of the carbocyclic ring, as in bc-T, leads to an anticlinal arrangement of γ and a somewhat attenuated but clear preference for an S-type furanose conformation.

### Oligonucleotide synthesis

**Scheme 3:**
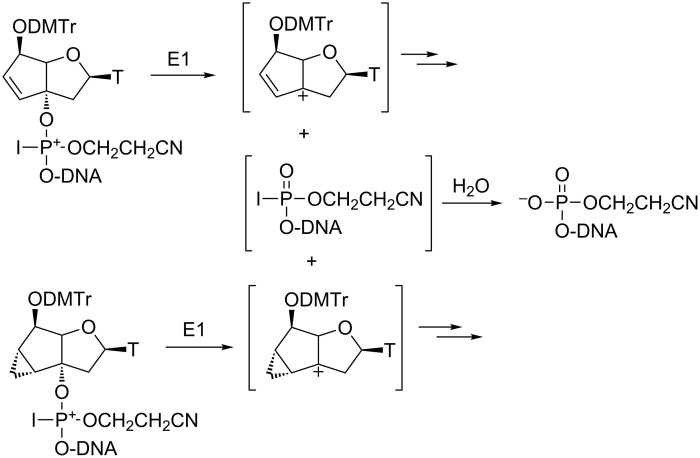
Pathways for elimination of the modified nucleotides during the oxidation step in oligonucleotide assembly.

The dodecamers **ON1–4**, shown in Table 2, containing one to two modifications, were synthesized in order to test the consequences of the two modified bicyclic nucleotides on RNA and DNA affinity. **ON5–7**, containing the known tc-T residues in the respective positions, were synthesized for comparison. **ON1–3** were assembled on the 1.3 μmol scale on a DNA synthesizer utilizing standard phosphoramidite chemistry protocols first. The trityl assay after incorporation of **13** and the subsequent building block revealed typically a drop of synthesis yield by roughly 20%. This was also reflected in the HPLC traces after final cleavage from the solid support (33% aq NH_3_, 55 °C, 16 h) which revealed besides the expected oligonucleotides **ON1–3** also truncated sequences corresponding to 5’-phosphorylated fragments arising from cleavage 3’ to the modification as determined by mass spectrometry. Re-subjection of the isolated full length oligonucleotides **ON1–3** to deprotection conditions did not lead to any further degradation, suggesting that E1 elimination of the 3’-P-unit occurs during the oxidation step of the modified residues, most likely on the level of the iodinated phosphite intermediate [35], leading to the formation of an allylic carbocation in the bc^en^-T unit and 5’-phosphorylated DNA fragment (Scheme 3).

The synthesis of oligonucleotides using building block **10** proved to be even more difficult. Using the standard phosphoramidite protocol, the oligonucleotide synthesis failed completely at the site of modification and not even traces of a full length oligonucleotide could be observed after chain assembly and deprotection. Only 5’-phosphorylated, truncated oligonucleotide fragments could be isolated. We reasoned that oxidation with iodine followed by E1 elimination of the 3’-P-unit happend also in this case, leaving behind an alpha-cyclopropyl cation that undergoes subsequent rearrangement (Scheme 3). The fact that elimination is quantitative in the iso-tc-T case may be explained by the release of ring strain during cyclopropyl rearrangement which contributes to the stabilization of the E1 transition state. Based on these assumptions we changed the oxidant from iodine to *t*-BuOOH, which has successfully been used in the past in the allyloxycarbonyl base- and phosphate protecting scheme for oligonucleotide synthesis [36]. Under these conditions, full length oligonucleotide **ON4** could be isolated in 80% as determined by trityl assay. Due to limited availability of the phosphoramidite building block **10**, only this particular oligonucleotide could be obtained in sufficient quantities for biophysical experimentation.

### *T*_m_ data

To assess DNA and RNA affinity of the two novel modifications we measured UV-melting curves at 260 nm. With a gradient of 0.5 °C/min the heating and cooling curves are superimposable, indicating equilibrium conditions and excluding degradation of the modified oligonucleotides under the conditions of measurement. The corresponding *T*_m_-data are summarized in Table 2.

**Table 2:** Sequence information and analytical data of **ON1–7** as well as *T*_m_ data from UV-melting curves (260 nm) in 10 mM NaH_2_PO_4_/Na_2_HPO_4_, 150 mM NaCl, pH 7.0. Duplex concentration: 1.2 μM.

	Sequence	Modification t	*T*_m_ vs DNA^a^	*T*_m_ vs RNA^a^

**ON1**	d(GGATGTTCtCGA)	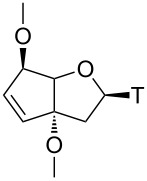	45.3 (−2.0)	45.6 (−2.2)
**ON2**	d(GGAtGTTCtCGA)		43.9 (−1.7)	45.0 (−1.4)
**ON3**	d(GGATGttCTCGA)		44.4 (−1.4)	46.2 (−0.8)

**ON4**	d(GGATGTTCtCGA)	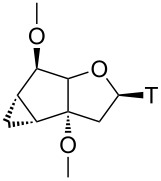	44.8 (−2.5)	43.0 (−4.8)

**ON5**	d(GGATGTTCtCGA)	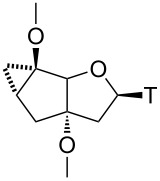	45.8 (−1.6)	46.6 (−1.2)
**ON6**	d(GGAtGTTCtCGA)		46.8 (−0.3)	46.8 (−0.5)
**ON7**	d(GGATGttCTCGA)		47.2 (−0.1)	49.6 (+0.9)

^a^*T*_m_ of unmodified duplex d(GGATGTTCTCGA): 47.3 °C vs DNA; 47.8 °C vs RNA; Δ*T*_m_ per modification in parenthesis.

The bc^en^-T modification destabilizes duplexes with complementary DNA by −1.4 to −2.0 °C per modification relative to dT in a somewhat sequence dependent context. If flanked by two pyrimidine nucleotides (**ON1**) the destabilization is higher as compared to purines as nearest neighbors (**ON2**). Two consecutive residues lead to less destabilization which is in line with earlier observations on tc-DNA where it was found that the highest *T*_m_/modification were observed in fully modified oligonucleotides [37]. Duplexes with RNA as complement are also destabilized albeit to a lesser extent (Δ*T*_m_/mod −0.8 to −2.2 °C). The same sequence dependence as for DNA as complement appears and again, two consecutive modifications are associated with the least depression in *T*_m_/modification. Thus, it turns out that bc^en^-DNA prefers RNA over DNA as a complement which is remarkable given that the parent nucleoside adopts a 2’-endo (S-type) sugar conformation and not a 3’endo (N-type) as do modifications that typically prefer RNA as complement (e.g., LNA). This is somewhat similar to observations with the α-L-LNA analogue which also prefers RNA over DNA as complement despite being a DNA mimic [38].

Also the iso-tc-T modification (**ON4**) turns out to destabilize duplexes with complementary DNA and RNA. However, in contrast to the bc^en^-modification, where there is essentially no difference in binding to DNA and RNA, destabilization of DNA as complement is lower (Δ*T*_m_ −2.5 °C) while that of RNA is higher (Δ*T*_m_ −4.8 °C), this despite the fact that the sugar conformations of the monomers (see Figure 2) are virtually identical. The differential behavior therefore has to be attributed to steric effects of the cyclopropyl methylene group on the adjacent 3’-phosphodiester function solely.

## Discussion

The two novel bc-/tc-modifications presented in this work are part of our endeavor to understand the structure/affinity relationship of this particular oligonucleotide molecular platform in more detail. More precisely we aimed with these modifications to learn how subtle structural changes influence not only the backbone torsion angle γ but also control the conformation of the furanose ring which is central for duplex structure and stability. From X-ray analysis of monomers (Table 2) we find that planarizing the C5’–C6’-bond (tc-nucleosides) leads to a *trans* orientation of torsion angle γ and some variability between S- and N-type furanose conformation, compared to the ring-saturated bc-nucleosides which have a stronger preference for S-type furanose conformation and also maintain the preference for the trans orientation of torsion angle γ. On the other hand, planarizing the C6’–C7’-bond, as in **8β** and **11β**, leads to a synclinal torsion angle γ and a consistent 2’-endo furanose conformation.

In order to correlate structural features of the four monomers under discussions with thermal affinity of correspondingly modified oligonucleotides we have summarized the Δ*T*_m_/modification data for RNA and DNA binding of the four modifications within the same sequence context for which data was available (Table 3). From the data it becomes evident that there is no clear correlation between torsion angle γ and affinity. For example bc^en^-T, having γ in the for duplexes natural *gauche* orientation, is more destabilizing than bc-T in which it is clearly in the unnatural *trans* orientation. However, there seems to be some correlation between the furanose pucker and affinity. There is a trend that nucleosides preferring an S-type sugar conformation (bc-T, bc^en^-T, iso-tc-T) prefer a DNA over an RNA complement. In the only nucleoside that shows some 3’-endo (N-type) character (tc-T) this is inverted. Probably the clearest correlation can be made regarding the effect of the cyclopropane ring in iso-tc-T. Compared to bc^en^-T, it becomes clear that the additional CH_2_ group destabilizes duplexes in an otherwise isostructural scaffold. This is most likely due to unfavorable steric interactions with the 3’-phosphate group. This negative effect is not unexpectedly most pronounced with RNA as a complement (A-type helical structure).

**Table 3:** Δ*T*_m_/modification data for four different bi/tricyclo modifications in one sequence context.

d(GGATGTTCtCGA) t =	Δ*T*_m_/modification vs DNA [°C]	Δ*T*_m_/modification vs RNA [°C]	furanose pucker	torsion angle γ

bc-T^a^	+1.5	−0.5	1’-exo	trans
bc^en^-T	−2.0	−2.2	2’-endo	gauche
tc-T	−1.6	−1.2	2’-endo/ 4’-endo	trans trans
iso-tc-T	−2.5	−4.8	2’-endo	gauche

^a^Ref. [27].

It has to be clearly noted here that an analysis based on single incorporations of bc- or tc-modifications does not necessarily reflect the effect of the same residues in fully modified oligonucleotides. For example, a bc-T residue stabilizes a duplex with complementary DNA in the above sequence context. However, a fully modified bc-oligonucleotide has no stabilizing effect upon binding to a DNA or RNA complement [20]. Along the same lines, a tc-T residue in the above sequence context destabilizes duplexes with both a DNA and an RNA complement. On the other hand fully modified tc-oligonucleotides stabilize duplexes with DNA and RNA by 1–3 °C per modification [37]. It thus appears that every modification of the DNA or RNA backbone with a bc- or tc-residue is associated with an energetic penalty which most likely arises from the local structural perturbation of the backbone at the site of modification. The more homogeneous the backbone becomes, the more dominant is the energetic benefit (or penalty) of the modification.

## Conclusion

We have synthesized the two novel, thymine containing bc-/tc-nucleosides **8** and **11β** and incorporated them into oligodeoxynucleotides. Analysis of the monomers by X-ray spectroscopy clearly show a high degree of similarity in the conformation of the underlying bicyclic scaffold of these two nucleosides. Thermal melting analysis of duplexes shows a destabilization with both DNA and RNA as complements. The destabilization is more expressed with the iso-tc-T unit and is due to steric interactions of the extra-CH_2_ group of the cyclopropane ring with the adjacent 3’-phosphate unit. A structure/affinity analysis including the known bc-T and tc-T nucleosides suggests that it is less the structural variety of torsion angle γ but more the furanose pucker (2’-endo vs 3’-endo) that governs affinity. Furthermore, from the accumulated set of *T*_m_ data available it becomes clear that Δ*T*_m_/modification data from oligonucleotides with single incorporations of members of the bc/tc-DNA family in general do not reflect the affinity profile of the corresponding fully modified oligonucleotides.

## Supporting Information

Experimental procedures and analytical data, including copies of ^1^H, ^13^C and ^31^P NMR spectra (where appropriate) for all new compounds as well as details for oligonucleotide synthesis and thermal melting experiments.

File 1 Experimental part.

## References

[1] Bennett C F, Swayze E E (2010). Annu Rev Pharmacol Toxicol.

[2] Vacek M, Sazani P, Kole R (2003). Cell Mol Life Sci.

[3] Rigo F, Hua Y, Chun S J, Prakash T P, Krainer A R, Bennett C F (2012). Nat Chem Biol.

[4] Lu Q-L, Yokota T, Takeda S, Garcia L, Muntoni F, Partridge T (2011). Mol Ther.

[5] Wood M J A, Gait M J, Yin H (2010). Brain.

[6] Passini M A, Bu J, Richards A M, Kinnecom C, Sardi S P, Stanek L M, Hua Y, Rigo F, Matson J, Hung G (2011). Sci Transl Med.

[7] Wheeler T M, Leger A J, Pandey S K, MacLeod A R, Nakamori M, Cheng S H, Wentworth B M, Bennett C F, Thornton C A (2012). Nature.

[8] Stenvang J, Petri A, Lindow M, Obad S, Kauppinen S (2012). Silence.

[9] Spitzer S, Eckstein F (1988). Nucleic Acids Res.

[10] Stein C A, Tonkinson J L, Yakubov L (1991). Pharmacol Ther.

[11] Manoharan M (1999). Biochim Biophys Acta.

[12] Pallan P S, Greene E M, Jicman P A, Pandey R K, Manoharan M, Rozners E, Egli M (2011). Nucleic Acids Res.

[13] Kalota A, Karabon L, Swider C R, Viazovkina E, Elzagheid M, Damha M J, Gewirtz A M (2006). Nucleic Acids Res.

[14] Prakash T P, Kawasaki A M, Wancewicz E V, Shen L, Monia B P, Ross B S, Bhat B, Manoharan M (2008). J Med Chem.

[15] Nielsen P E, Egholm M, Berg R H, Buchardt O (1991). Science.

[16] Summerton J E (1999). Biochim Biophys Acta.

[17] Veedu R N, Wengel J (2010). Chem Biodiversity.

[18] Imanishi T, Obika S (2002). Chem Commun.

[19] Hendrix C, Rosemeyer H, Verheggen I, Van Aerschot A, Seela F, Herdewijn P (1997). Chem–Eur J.

[20] Bolli M, Trafelet H U, Leumann C (1996). Nucleic Acids Res.

[21] Renneberg D, Leumann C J (2002). J Am Chem Soc.

[22] Renneberg D, Bouliong E, Reber U, Schümperli D, Leumann C J (2002). Nucleic Acids Res.

[23] Murray S, Ittig D, Koller E, Berdeja A, Chappell A, Prakash T P, Norrbom M, Swayze E E, Leumann C J, Seth P P (2012). Nucleic Acids Res.

[24] Tarköy M, Leumann C (1993). Angew Chem, Int Ed Engl.

[25] Šilhár P, Leumann C J (2010). Bioorg Med Chem.

[26] Luisier S, Leumann C J (2010). Heterocycles.

[27] Luisier S, Leumann C J (2008). ChemBioChem.

[28] Lietard J, Leumann C J (2012). J Org Chem.

[29] Lietard J, Ittig D, Leumann C J (2011). Bioorg Med Chem.

[30] Dugovic B, Leumann C J (2014). J Org Chem.

[31] Vorbrüggen H, Krolikiewicz K, Bennua B (1981). Chem Ber.

[32] Vorbrüggen H, Bennua B (1981). Chem Ber.

[33] Tarköy M, Bolli M, Schweizer B, Leumann C (1993). Helv Chim Acta.

[34] Steffens R, Leumann C (1997). Helv Chim Acta.

[35] Scheuer-Larsen C, Dahl B M, Wengel J, Dahl O (1998). Tetrahedron Lett.

[36] Hayakawa Y, Wakabayashi S, Kato H, Noyori R (1990). J Am Chem Soc.

[37] Ittig D, Gerber A-B, Leumann C J (2011). Nucleic Acids Res.

[38] Ellemann Nielsen K M, Petersen M, Håkansson A E, Wengel J, Jacobsen J P (2002). Chem–Eur J.

