# Supplementary material for: Structure/affinity studies in the bicyclo-DNA series: Synthesis and properties of oligonucleotides containing bcen-T and iso-tricyclo-T nucleosides
Source: Beilstein J Org Chem. 2014 Aug 12;10:1840–7. doi: 10.3762/bjoc.10.194 (PMC4142851; doi:10.3762/bjoc.10.194)
Supplement: File 1 — Experimental part. [file Beilstein_J_Org_Chem-10-1840-s001.pdf]

Supporting Information

for

Structure/affinity studies in the bicyclo-DNA series: Synthesis and properties of oligonucleotides containing bc^{en}-T and iso-tricyclo-T nucleosides

Branislav Dugovic, Michael Wagner and Christian J. Leumann*

Address: Department of Chemistry and Biochemistry, University of Bern, Freiestrasse 3, CH-3012 Bern, Switzerland

Email: Christian J. Leumann - christian.leumann@dcb.unibe.ch

*Corresponding author

Experimental part

Pages S2–S27	Data on synthesis of compounds **1–13**<br>**Figures S1–S16:** ^{1}H, ^{13}C and ^{31}P NMR spectra of compounds **1–13**
Page S28	Crystal structure determination - General
Page S29	**Figure S17:** ORTEP view of compound **8**
Pages S29–S36	**Tables S1–S9:** Crystal structure data of compound **8**
Page S37	**Figure S18:** ORTEP view of compound **11β**
Pages S37–S43	**Tables S10–S18:** Crystal structure data of compound **11β**
Page S44	**Figure S19:** ORTEP view of compound **tc-T**
Pages S44–S51	**Tables S19–S22:** Crystal structure data of compound **tc-T**
Page S52	General procedures for synthesis, deprotection and purification of **ON1–7**
Page S53	**Table S23:** ESI-M and HPLC characterization of **ON1–7**
Page S54–S57	Scans of ESI^{-}-MS of oligonucleotides **ON1–4**

(2*S*,3a*R*,6*R*,6a*R*)-2-methoxy-3,3a,6,6a-tetrahydro-2*H*-cyclopenta[*b*]furan-3a,6-diol (2). The solution ketone **1** (1.15 g, 6.76 mmol) in MeOH (68 mL) and $CeCl_3.7H_2O$ (2.52 g, 6.76 mmol) was stirred at ambient temperature for 30 min. It was cooled down to -78°C and $NaBH_4$ (0.26 g, 6.76 mmol) was added during 20 min in three equal portions. Resulting mixture was stirred for 1.5 h at the same temperature. Then the cooling bath was removed, stirring continued for 30 min at RT and afterwards the solvent was evaporated. Residue was purified by CC (3 % MeOH in CH_2Cl_2) to provide 0.85 g (73%, colourless oil) of **2** and 0.11 g (9%, white solid) of **epi-2**.

*data for **2**:* R_f (6% MeOH in CH_2Cl_2) = 0.28; ^{1}H NMR (CD_3OD, 300 MHz): δ = 5.82 (ddd, *J* = 5.7, 1.7, 0.6 Hz, 1H), 5.74 (ddd, *J* = 5.7, 1.8, 0.8 Hz, 1H; H-C(4,5)), 5.11 (dd, *J* = 5.4, 2.3 Hz, 1H; H-C(2)), 4.80 (dt, *J* = 5.7, 1.8 Hz, 1H; H-C(6)), 4.24 (d, *J* = 5.5 Hz, 1H; H-C(6a)), 3.39 (s, 3H; OCH_3), 2.28 (dd, *J* = 13.8, 5.4 Hz, 1H; H-C(3)), 2.10 (dd, *J* = 13.8, 2.3 Hz, 1H; H-C(3)) ppm; ^{13}C NMR (CD_3OD, 75 MHz): δ = 137.2, 136.0 (2×d, C(4,5)), 108.7 (d, C(2)), 91.7 (s, C(3a)), 86.3 (d, C(6a)), 76.1 (d, C(6)), 55.4 (q, OCH_3), 47.7 (t, C(3)) ppm; ESI$^+$-HRMS: calcd for $C_8H_{12}NaO_4$: 195.0628 $[M+Na]^+$; found: 195.0629.

data for **epi-2**: R_f (6% MeOH in CH_2Cl_2) = 0.19; ^{1}H NMR (CD_3OD, 300 MHz): δ = 5.91 (dt, *J* = 5.6, 0.9 Hz, 1H), 5.72 (ddd, *J* = 5.6, 2.2, 1.1 Hz, 1H; H-C(4,5)), 5.04 (dd, *J* = 4.3, 2.1 Hz, 1H; H-C(2)), 4.41 (dt, *J* = 2.0, 0.9 Hz, 1H; H-C(6)), 4.21-4.20 (m, 1H; H-C(6a)), 3.36 (s, 3H; OCH_3), 2.16-2.01 (m, 2H; H-C(3)) ppm; ^{13}C NMR (CD_3OD, 75 MHz): δ = 139.4, 133.5 (2×d, C(4,5)), 108.3 (d, C(2)), 97.2 (d, C(6a)), 91.3 (s, C(3a)), 81.2 (d, C(6)), 55.1 (q, OCH_3), 46.1 (t, C(3)) ppm; ESI$^+$-HRMS: calcd for $C_8H_{12}NaO_4$: 195.0628 $[M+Na]^+$; found: 195.0635.

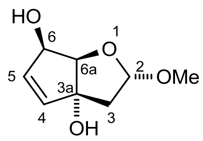

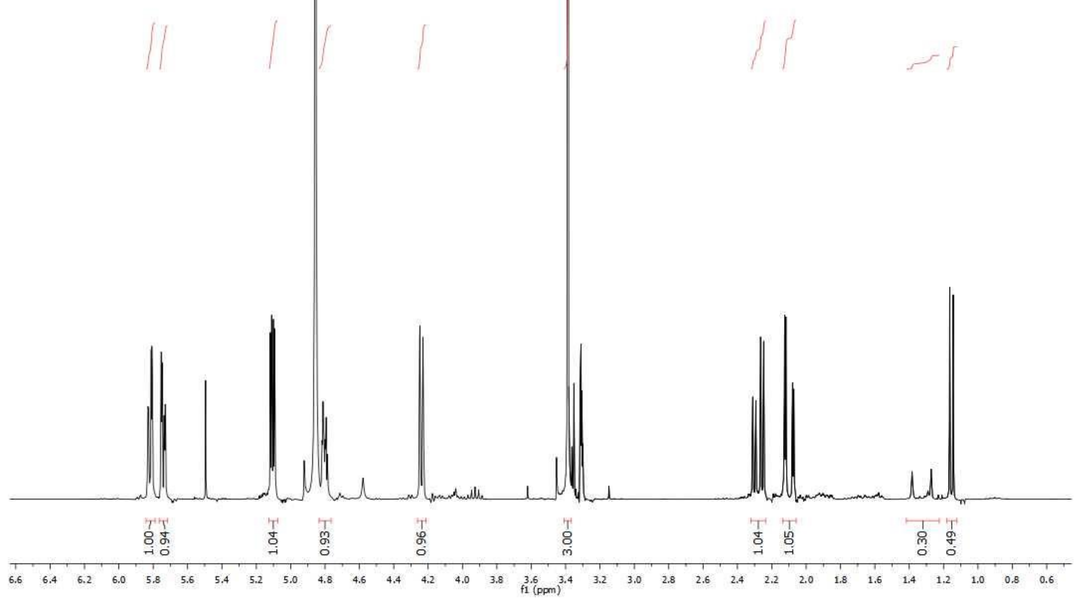

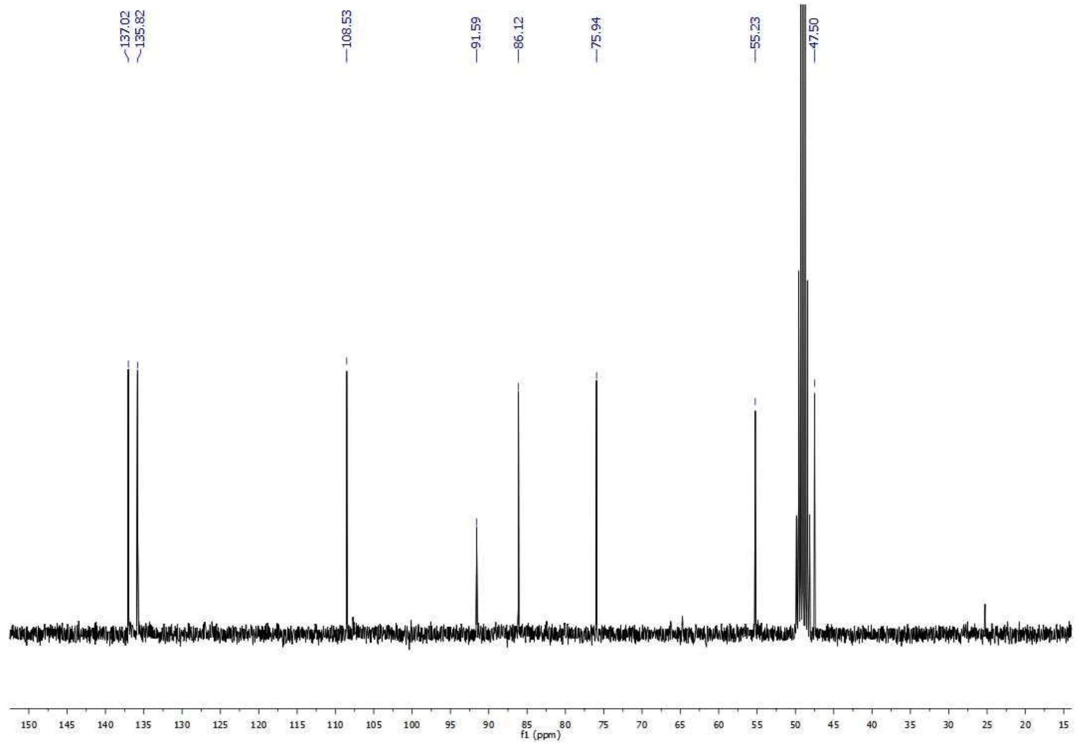

Figure S1: ^{1}H (CD_3OD, 300 MHz) and ^{13}C NMR (CD_3OD, 75 MHz) spectrum of **2**

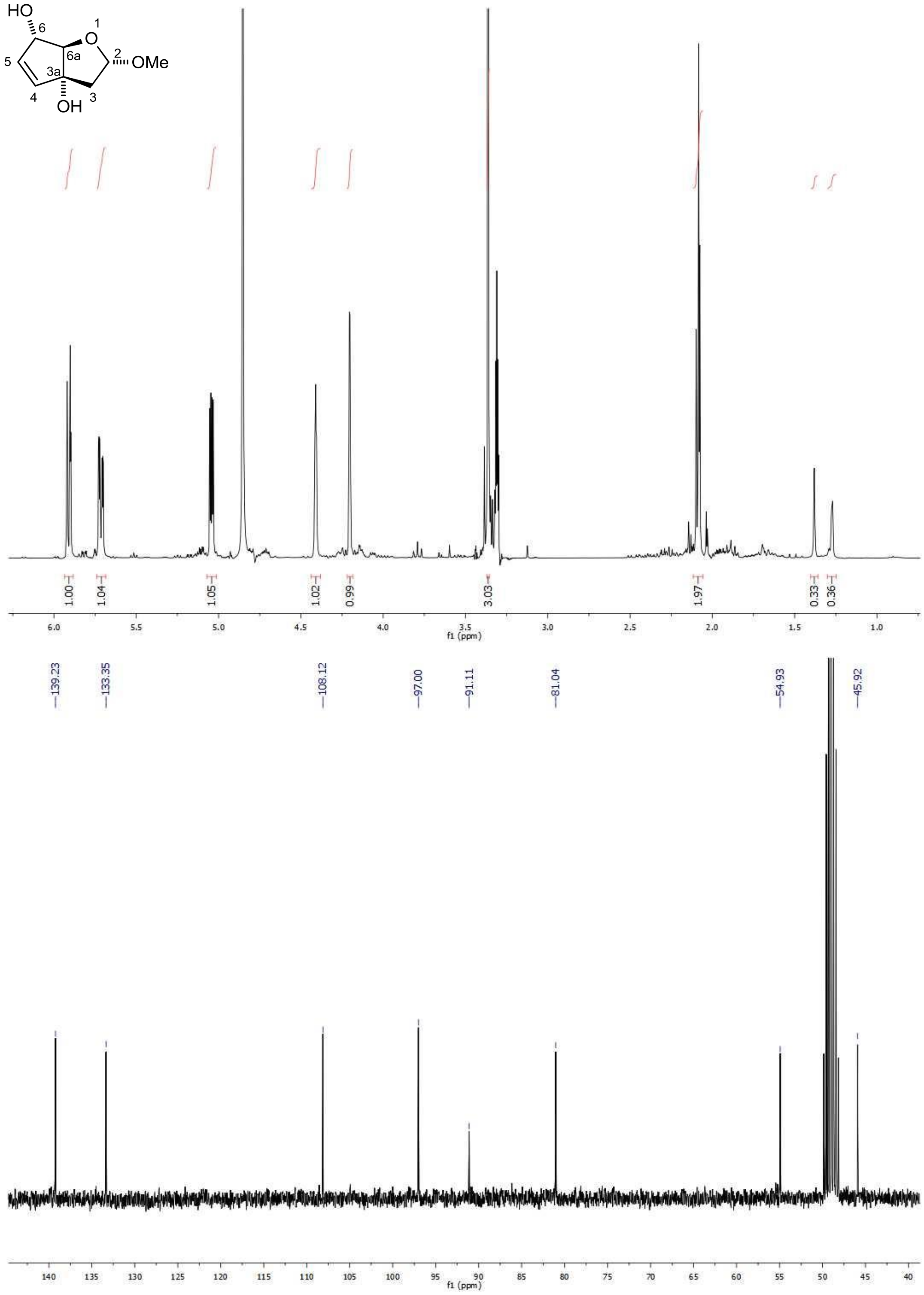

Figure S2: ^{1}H (CD_3OD, 300 MHz) and ^{13}C NMR (CD_3OD, 75 MHz) spectrum of **epi-2**

(2*S*,3a*R*,6*R*,6a*R*)-6-{(*tert*-Butyl)dimethylsilyl]oxy}-2-methoxy-3,3a,6,6a-tetrahydro-2*H*-cyclopenta[*b*]furan-3a-ol (3). To diol **2** (0.85 g, 4.9 mmol) dissolved in dry CH_2Cl_2 (50 mL) were successively added imidazole (1 g, 14.8 mmol) and TBDMSCl (1.12 g, 7.4 mmol). The reaction mixture

was stirred under an argon atmosphere at ambient temperature overnight. After addition of 1N HCl (30 mL), the aqueous phase was extracted with CH_2Cl_2 (3×) and the organic layer was dried with $MgSO_4$ and evaporated under reduced pressure. The yellowish oil was purified by CC (EtOAc/Hexane 1:1) to afford **3** (1.12 g, 79%) as a yellowish oil.

*data for **3:*** R_f (3.5% MeOH in CH_2Cl_2) = 0.53; ^{1}H NMR (400 MHz, $CDCl_3$) δ = 5.87 (dd, 1H, *J* = 5.8, 0.9 Hz), 5.70 (dd, 1H, *J* = 5.9, 2.1 Hz, H-C(4,5)), 5.16 (dd, 1H, *J* = 4.8, 0.9 Hz, H-C(2)), 4.73 (ddd, 1H, *J* = 5.7, 2.2, 0.9 Hz, H-C(6)), 4.23 (d, 1H, *J* = 5.7 Hz, H-C(6a)), 3.37 (s, 3H, OCH_3), 2.84 (brs, 1H, OH), 2.17 (dd, 1H, *J* = 13.5, 4.8 Hz), 2.06 (dd, 1H, *J* = 13.5, 0.9 Hz, H-C(3)), 0.87 (s, 9H, $SiC(CH_3)_3$), 0.07 (s, 6H, $Si(CH_3)_2$) ppm. ^{13}C NMR (75 MHz, $CDCl_3$) δ = 135.6 (d), 134.8 (d, C(4,5)), 108.9 (d, C(2)), 91.1 (s, C(3a)), 88.1 (d, C(6a)), 74.7 (d, C(6)), 54.9 (q, OCH_3), 46.9 (t, C(3)), , 26.1 (q, $SiC(CH_3)_3$), 18.6 (s, $SiC(CH_3)_3$), -4.4, -4.8 (2×$Si(CH_3)_2$) ppm. ESI-HRMS: calc. for $C_{14}H_{26}NaO_4Si$ 309.1498 $[M+Na]^+$, found 309.1496.

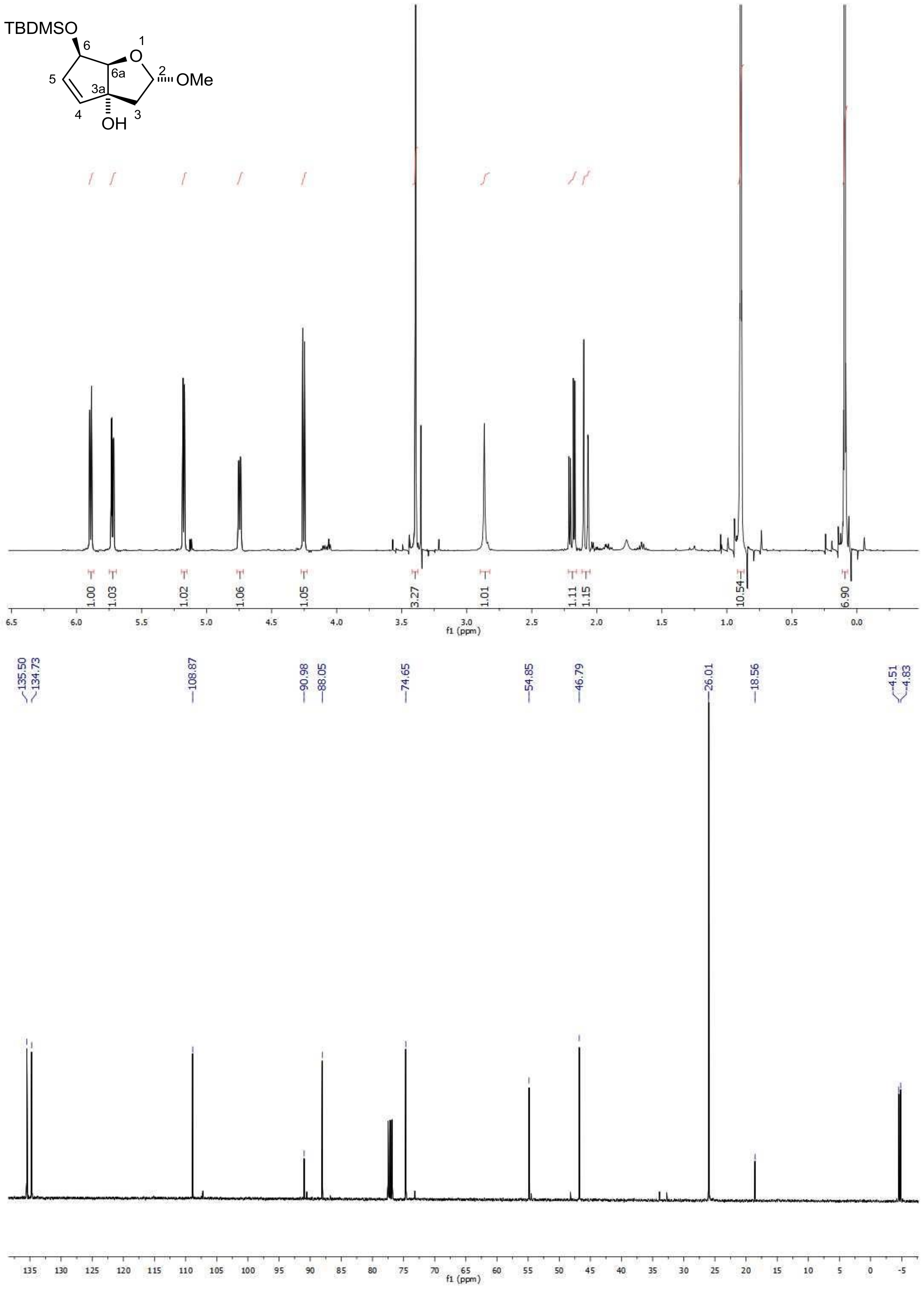

Figure S3: ^{1}H ($CDCl_3$, 400 MHz) and ^{13}C NMR ($CDCl_3$, 75 MHz) spectrum of **3**

(2*S*,3a*R*,3b*R*,4a*S*,5*R*,5a*R*)-5-{(*tert*-Butyldimethylsilyl)oxy}-2-methoxy-octahydro-cyclopropa[3,4]cyclopenta[1,2-*b*]furan-3a-ol (4). To alcohol **3** (1.12 g, 3.9 mmol) dissolved in dry CH_2Cl_2 (39 mL) under an argon atmosphere a solution of Et_2Zn 1M in hexane (23.4 mL, 23.4 mmol) was added

dropwise at 0 °C and mixture was stirred at this temperature for 15 min. Afterwards, CH_2I_2 (3.1 mL, 39.0 mmol) was added, the reaction mixture was allowed to warm to ambient temperature and it was stirred overnight. The reaction was quenched by the addition of NH_4Cl. After separation of the phases the organic layer was washed with 1N $Na_2S_2O_3$. The combined aqueous layers were extracted with EtOAc and dried with $MgSO_4$. The solvents were evaporated under reduced pressure. The yellowish oil was purified by CC (EtOAc/Hexane 3:7) to afford **4** (1.01 g, 86%) as colourless solid.

*data for **4:*** R_f (EtOAc/Hexane 3:7) = 0.38; ^{1}H NMR (400 MHz, $CDCl_3$) δ = 5.07 (d, 1H, *J* = 4.9 Hz, H-C(1)), 4.06 (d, 1H, *J* = 5.2, H-C(5)), 3.92 (d, 1H, *J* = 5.1, H-C(4)), 3.34 (s, 3H, OCH_3), 3.23 (brs 1H, OH), 2.30 (dd, 1H, *J* = 13.1, 4.9, H-C(2)), 2.03 (d, 1H, *J* = 13.1, H-C(2)), 1.65 (ddd, 1H, *J* = 7.9, 5.9, 3.7, H-C(7)), 1.38 (ddd, 1H, *J* = 8.7, 5.8, 4.5, H-C(6)), 0.87 (s, 9H, $C(CH_3)_3$), 0.63-0.57 (m, 1H, H-C(8)), 0.49-0.46 (m, 1H, H-C(8)), 0.05, 0.03(2×s, 2×3H, $Si(CH_3)_2$) ppm. ^{13}C NMR (75 MHz, $CDCl_3$) δ = 108.9 (d, C(1)), 90.0 (d, C(4)), 88.4 (s, C(3)), 71.9 (d, C(5)), 55.0 (q, OCH_3), 45.2 (t, C(2)), 26.1 (q, $SiC(CH_3)_3$), 23.9 (d, C(6)), 22.8 (d, C(7)), 18.4 (s, $SiC(CH_3)_3$), 7.8 (t, C(8)), -4.4, -5.0 (q, $Si(CH_3)_2$) ppm. ESI-HRMS: calc. for $C_{15}H_{28}NaO_4Si$ 323.1655 $[M+Na]^+$, found 323.1649.

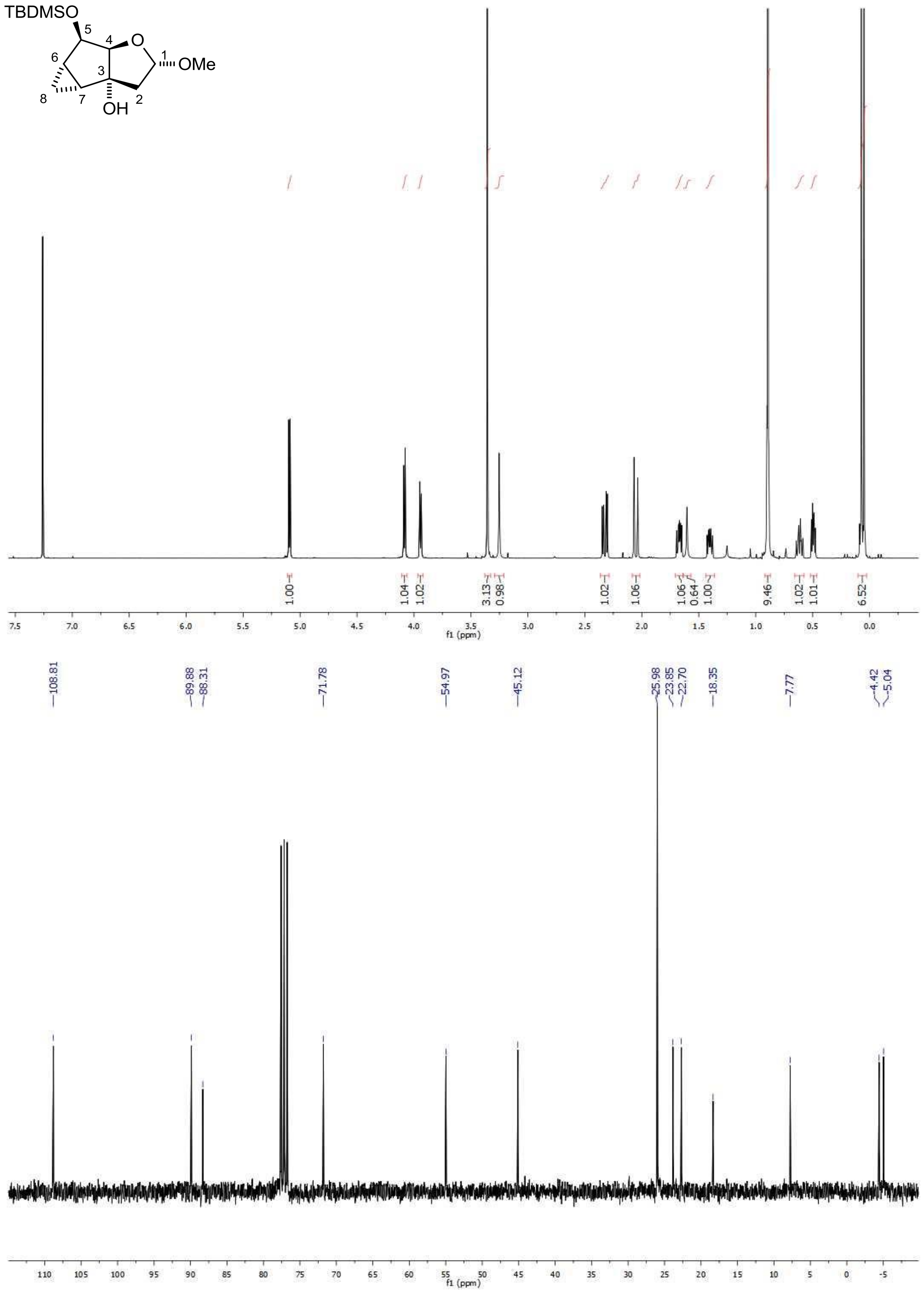

Figure S4: ^{1}H ($CDCl_3$, 400 MHz) and ^{13}C NMR ($CDCl_3$, 75 MHz) spectrum of **4**

(2*S*,3a*R*,3b*R*,4a*S*,5*R*,5a*R*)-5-{(*tert*-Butyldimethylsilyl)oxy}-2-methoxy octahydro cyclopropa[3,4]cyclopenta[1,2-*b*]furan-3a-yl pivalate (5). To mixture of the alcohol **4** (0.37 g, 1.23 mmol) and DMAP (0.90 g, 7.39 mmol) in pyridine (1 mL) and 1,2-dichloroethane (15 mL) cooled in an ice

bath to 0 °C was added PivCl (0.9 mL, 7.39 mmol). After 10 min the ice bath was removed and the reaction mixture was heated at 70 °C overnight. The mixture was poured into sat. aq. $NaHCO_3$ and extracted with CH_2Cl_2 (3×). The organic layer was dried with $MgSO_4$, solvents were removed *in vacuo*. Purification by CC (EtOAc/Hexane 1:9) afforded title compound **19** (0.5 g, quant.) as a colourless oil.

*data for **5**:* R_f (EtOAc/Hex 1:1) = 0.8; ^1H NMR (400 MHz, $CDCl_3$) δ = 5.14 (dd, 1H, *J* = 5.8, 1.0 Hz, H-C(1)), 4.19 (d, 1H, *J* = 5.2 Hz, H-C(5)), 4.03 (d, 1H, *J* = 5.2 Hz, H-C(4)), 3.27 (s, 3H, OCH_3), 2.47 (d, 1H, *J* = 14.6 Hz, H-C(2)), 2.36 (dd, 1H, *J* = 14.6, 5.9 Hz, H-C(2)), 2.26 (ddd, 1H, *J* = 8.1, 6.0, 3.7 Hz, H-C(6/7)), 1.45 (ddd, 1H, *J* = 8.7, 5.8, 4.6 Hz, H-C(6/7)), 1.16 (s, 9H, $COC(CH_3)_3$), 0.89 (s, 9H, $SiC(CH_3)_3$), 0.56-0.50 (m, 1H, H-C(8)), 0.26 (ddd, 1H, *J* = 5.9, 4.5, 3.9 Hz, H-C(8)), 0.07, 0.06 (s, 2×3H, $Si(CH_3)_2$), ppm. ^{13}C NMR (75 MHz, $CDCl_3$) δ = 178.5 (s, $COC(CH_3)_3$), 109.1 (d, C(1)), 94.3 (s, C(3)), 89.1 (d, C(4)), 71.2 (d, C(5)), 54.9 (q, OCH_3), 42.4 (t, C(2)), 39.1 (s, $COC(CH_3)_3$), 27.2 (q, $COC(CH_3)_3$), 26.1 (q, $SiC(CH_3)_3$), 25.5 (d,C(6/7)), 22.9 (d, C(6/7)), 18.5 (s, $SiC(CH_3)_3$), 7.9 (t, C(8)), -4.3, -5.0 (q, $Si(CH_3)_2$) ppm. ESI-HRMS: calc. for $C_{20}H_{36}NaO_5Si$ 40 7.2230 $[M+Na]^+$, found 407.2224.

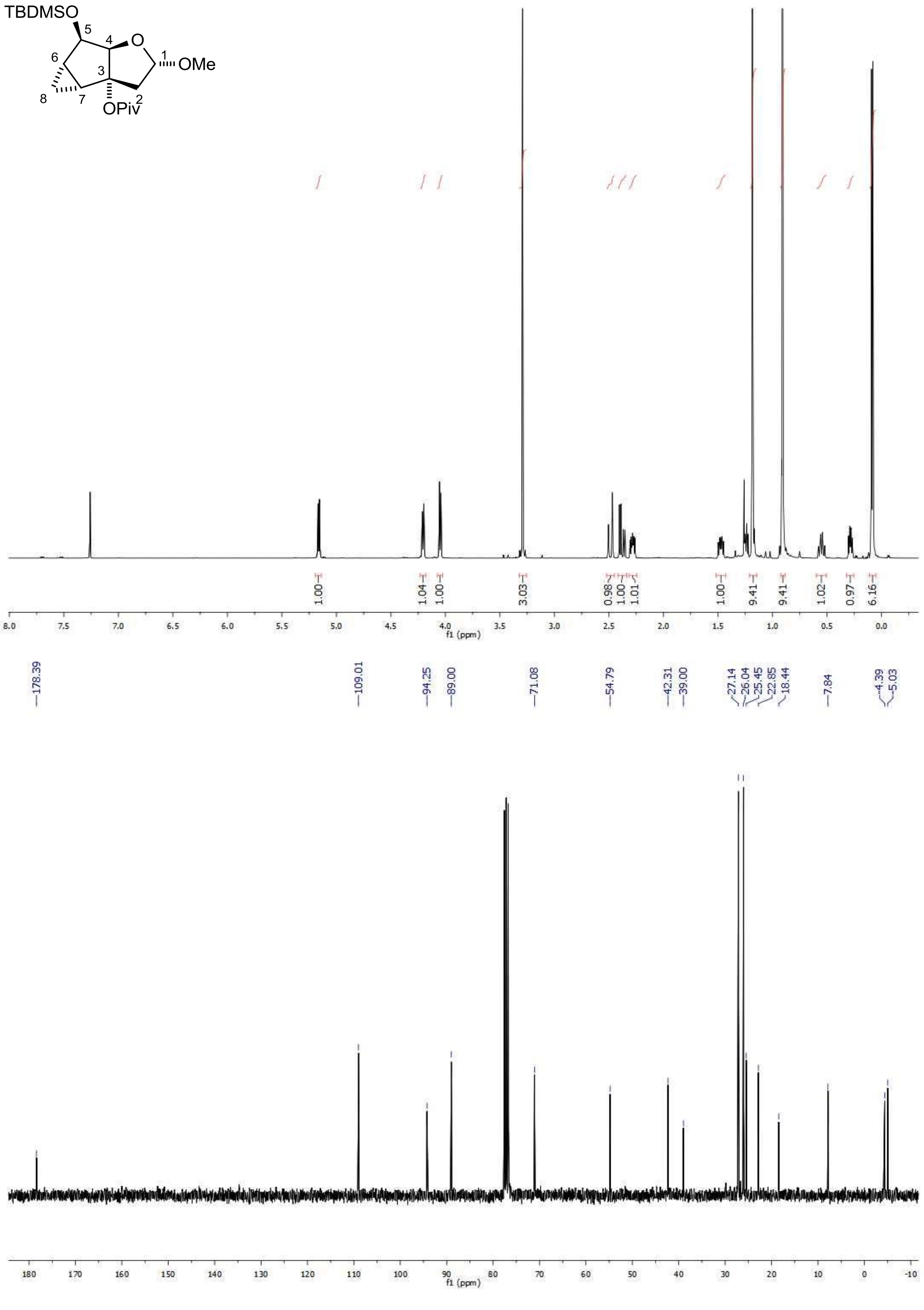

Figure S5: ^{1}H ($CDCl_3$, 400 MHz) and ^{13}C NMR ($CDCl_3$, 75 MHz) spectrum of **5**

(2*S*,3a*R*,3b*R*,4a*S*,5*R*,5a*R*)-5-Hydroxy-2-methoxy octahydro cyclopropa[3,4]cyclopenta[1,2-*b*]furan-3a-yl pivalate (6). The compound **5** (0.5 g, max 1.23 mmol) was dissolved in dry THF (15 mL). To the solution was added 1M TBAF in THF (1.85 mL, 1.85 mmol) and it was stirred at RT overnight. The reaction was

quenched by adding sat. aq. $NaHCO_3$ and then extracted with EtOAc (3×) and dried over $MgSO_4$. The compound was purified by CC (EtOAc/Hex 1:1) to afford **6** (314 mg, 95 %) as a colourless solid.

*data for **6**:* R_f (EtOAc/Hex 1:1) = 0.51; ^{1}H NMR (400 MHz, $CDCl_3$) δ = 5.20 (d, 1H, *J* = 5.4 Hz, H-C(1)), 4.28 (d, 1H, *J* = 5.3 Hz, H-C(4)), 4.06 (d, 1H, *J* = 5.3 Hz, H-C(5)), 3.30 (s, 3H, OCH_3), 2.61 (d, 1H, *J* = 14.7 Hz, H-C(2)), 2.54 (d, 1H, *J* = 0.7, OH), 2.30-2.22 (m, 2H, H-C(2), H-C(6/7)), 1.69 (ddd, 1H, *J* = 9.0, 5.1, 5.1, H-C(6/7)), 1.17 (s, 9H, $C(CH_3)_3$), 0.63 (ddd, 1H, *J* = 8.8, 5.8, 5.8 Hz, H-C(8)), 0.26 (ddd, 1H, *J* = 5.8, 4.2, 4.2 Hz, H-C(8)) ppm. ^{13}C NMR (75 MHz, $CDCl_3$) δ = 178.2 (s, $COC(CH_3)_3$), 149.1 (d,C(1)), 95.0 (s, C(3)), 89.1 (d, C(4)), 69.8 (d, C(5)), 55.1 (q, OCH_3), 42.7 (t, C(2)), 39.0 (s, $COC(CH_3)_3$), 27.2 (q, $COC(CH_3)_3$), 25.4 (d, C(6/7)), 24.1 (d, C(6/7)), 7.5 (t, C(8)) ppm. ESI-HRMS: calc. for $C_{14}H_{22}NaO_5$ 293.1365 $[M+Na]^+$, found 293.1359.

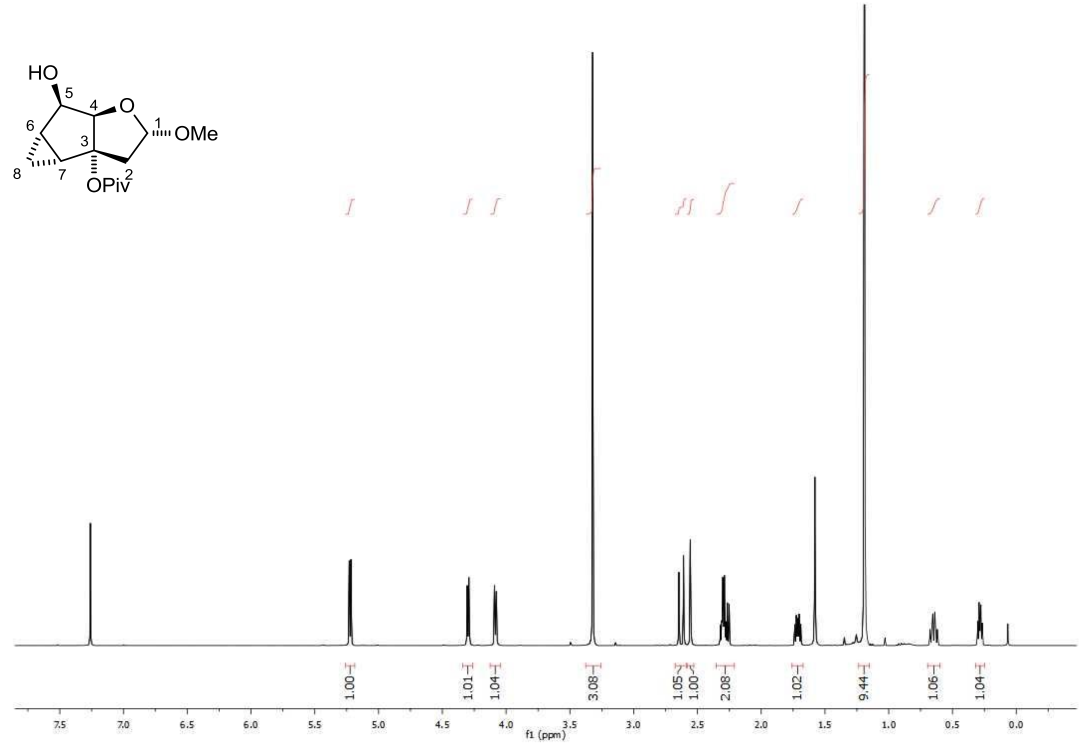

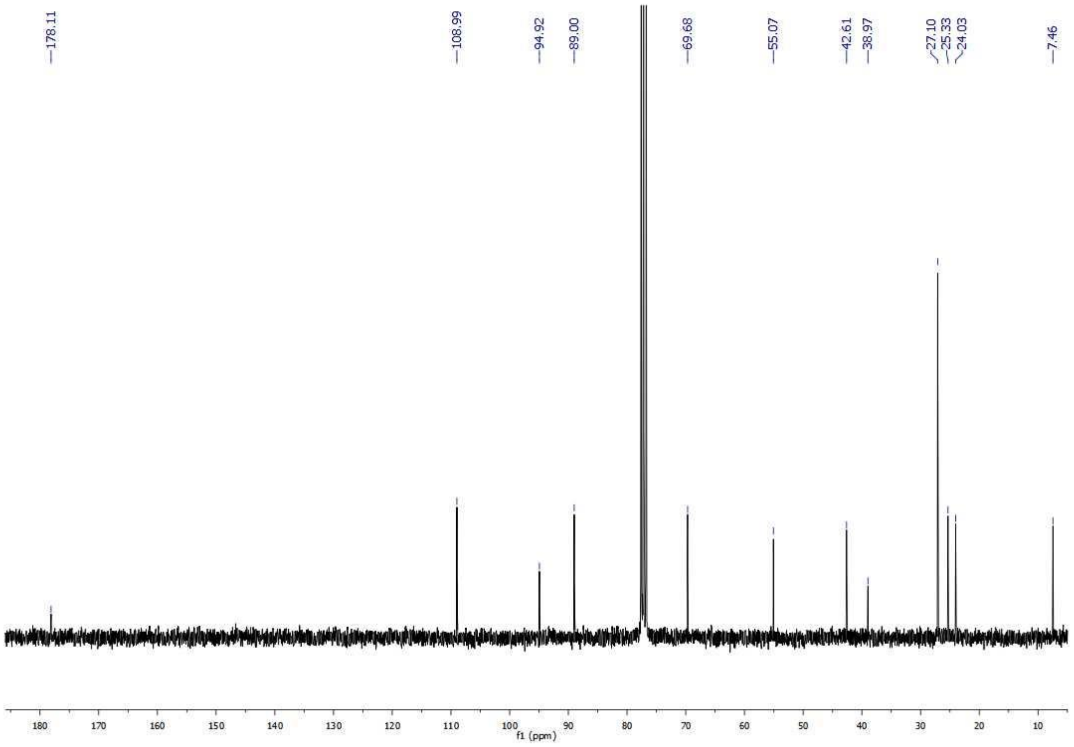

Figure S6: ^{1}H ($CDCl_3$, 400 MHz) and ^{13}C NMR ($CDCl_3$, 75 MHz) spectrum of **5**

(5’*R*)-1-(3’-Pivaloyl-2’-deoxy-3’,5’-ethano-6’,7’-methano-α-and-β-D-ribofuranosyl)thymine (7α and 7β). The alcohol **6** (0.31 g, 1.15 mmol) and thymine (0.29 g, 2.29 mmol) suspended in dry acetonitrile (12 mL) were treated with BSA (1.4 mL, 5.73 mmol) at 0 °C. After a clear solution had been formed (1 h) the $SnCl_4$ (0.54 mL, 4.59 mmol) was added at 0 °C and the reaction was stirred for 17 h while the temperature slowly raised up to RT. The reaction was diluted with EtOAc and poured into satd. aq. $NaHCO_3/H_2O$ (2:1, 50 mL). The aqueous phase was extracted with EtOAc (4×) and dried over $MgSO_4$. Solvents were removed *in vacuo*, the rest was taken up in CH_2Cl_2, filtered through Celite and concentrated. Purification by CC (2 ⟶ 5% MeOH in CH_2Cl_2) provided 235 mg (56%) of **7β** and 93 mg (22%) of **7α** (contamined by 8% of **7β**) as white foams.

*data for **7α**:* R_f (5 % MeOH in CH_2Cl_2) = 0.29; ^{1}H NMR (300 MHz, CD_3OD) δ = 9.47 (brs, 1H, NH), 7.21 (d, 1H, *J* = 1.1 Hz, H-C(6)), 6.44 (dd, 1H, *J* = 7.9, 1.8 Hz, H-C(1‘)), 4.46 (d, 1H, *J* = 4.9 Hz, H-C(4‘)), 4.22 (d, 1H, *J* = 5.0 Hz, H-C(5‘)), 3.28 (brs, 1H, OH), 2.95 (dd, 1H, *J* = 15.9, 8.0 Hz, H-C(2‘)), 2.59 (dd, 1H, *J* = 15.8, 0.8 Hz, H-C(2‘)), 2.38 (ddd, 1H, *J* = 8.1, 5.9, 3.7 Hz, H-C(7‘)), 1.86 (d, 3H, *J* = 0.7 Hz, CH_3), 1.71 (ddd, 1H, *J* = 8.9, 5.2, 5.2 Hz, H-C(6‘)), 1.15 (s, 9H, $COC(CH_3)_3$), 0.72-0.66, 0.34-0.29 (2×m, 2×1H, H-C(8‘)) ppm; ^{13}C NMR (75 MHz, $CDCl_3$) δ = 178.0 (s, $COC(CH_3)_3$), 164.1 (s, C(4)), 150.4 (d, C(2)), 135.5 (d, C(6)), 110.6 (d, C(5)), 94.8 (s, C(3‘)), 91.0, 89.9 (2×d, C(5‘), C(1‘)), 70.4 (d, C(4‘)), 43.5 (t, C(2‘)), 39.1 (s, $COC(CH_3)_3$), 27.1 (q, $COC(CH_3)_3$), 23.9, 23.3 (2×d, C(6‘), C(7‘)), 12.7 (q, CH_3), 7.8 (t, C(8‘)) ppm; ESI-HRMS: calc. for $C_{18}H_{25}N_2O_6$ 365.1713 $[M+Na]^+$, found 365.1719.

*data for **7β**:* R_f (5 % MeOH in CH_2Cl_2) = 0.34. ^{1}H NMR (300 MHz, $CDCl_3$) δ = 9.13 (brs, 1H, NH),7.74 (d, 1H, *J* = 0.7 Hz, H-C(6)), 6.38 (dd, 1H, *J* = 10.8, 4.8 Hz, H-C(1’)), 4.23 (dd, 1H, *J* = 4.9, 2..3 Hz, H-C(5’)), 4.10 (d, 1H, *J* = 5.1 Hz, H-C(4’)), 3.75-3.73 (m, 1H, OH), 2.77 (dd, 1H, *J* = 13.9, 4.8 Hz, H-C(2’)), 2.45-2.34 (m, 2H, H-

C(2’), H-C(6’)), 1.81 (s, 3H, CH_3), 1.76-1.70 (m, 1H, H-C(7’)), 1.18 (s, 9H, $COC(CH_3)_3$), 0.70-0.63, 0.30-0.26 (2×m, 2×1H, H-C(8’)) ppm; ^{13}C NMR (75 MHz, $CDCl_3$) δ = 178.2 (s, $COC(CH_3)_3$), 164.0 (s, C(4)), 150.6 (s, C(2)), 136.6 (d, C(6)), 111.1 (s, C(5)), 94.7 (d, C(3‘)), 87.9 (d, C(4‘)), 86.6 (d, C(1’)), 69.5 (d, C(5’)), 39.4 (t, C(2’)), 39.1 (s, $COC(CH_3)_3$), 27.1 (q, $COC(CH_3)_3$), 24.7 (d, C(7’)), 22.3 (d, C(6’)), 12.4 (q, CH_3), 6.9 (t, C(8’)) ppm. ESI-HRMS: calc. for $C_{18}H_{24}N_2NaO_6$ 387.1532 $[M+Na]^+$, found 387.1510

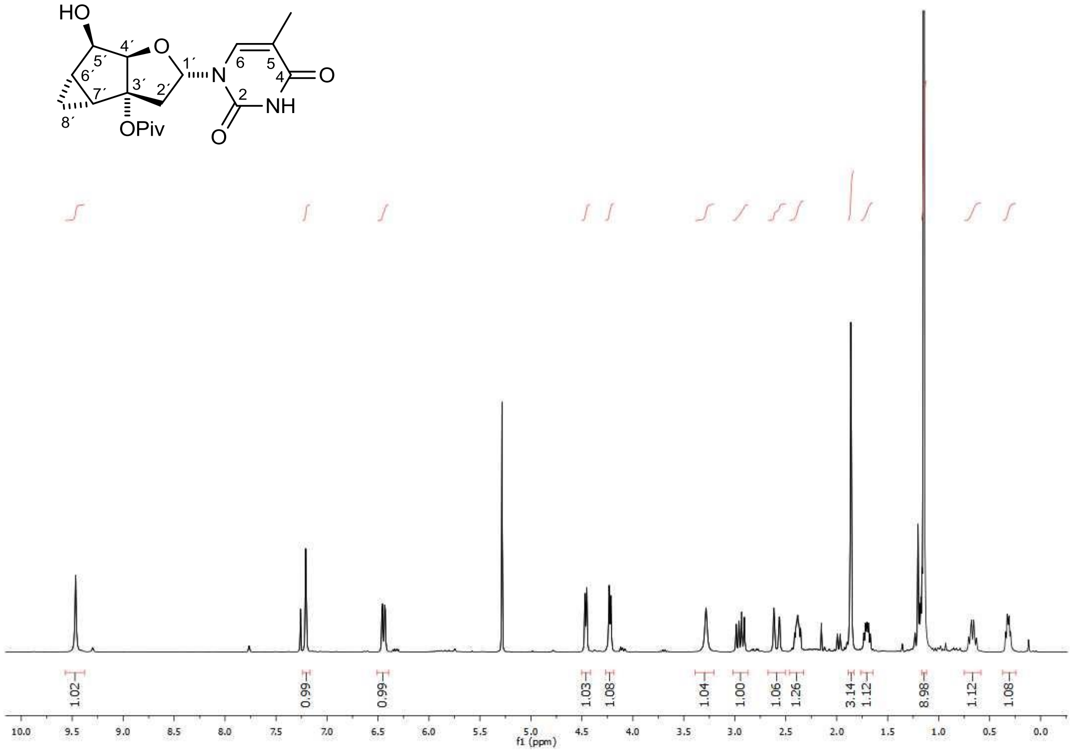

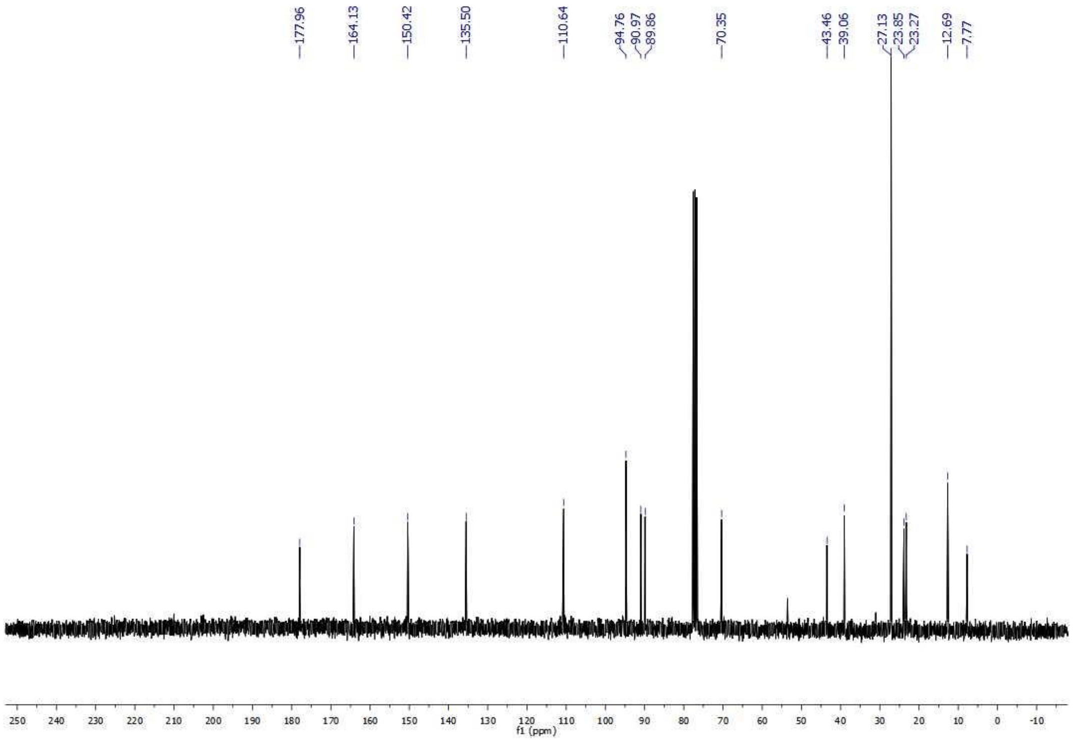

Figure S7: ^{1}H ($CDCl_3$, 400 MHz) and ^{13}C NMR (CD_3OD, 75 MHz) spectrum of **7α**

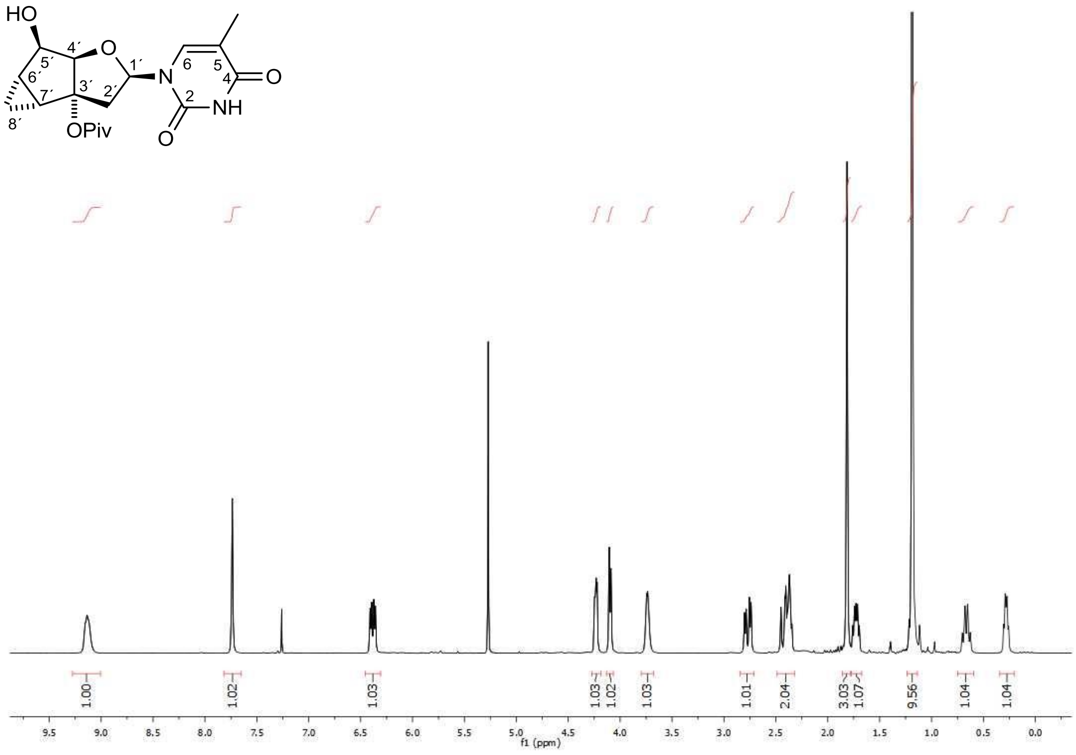

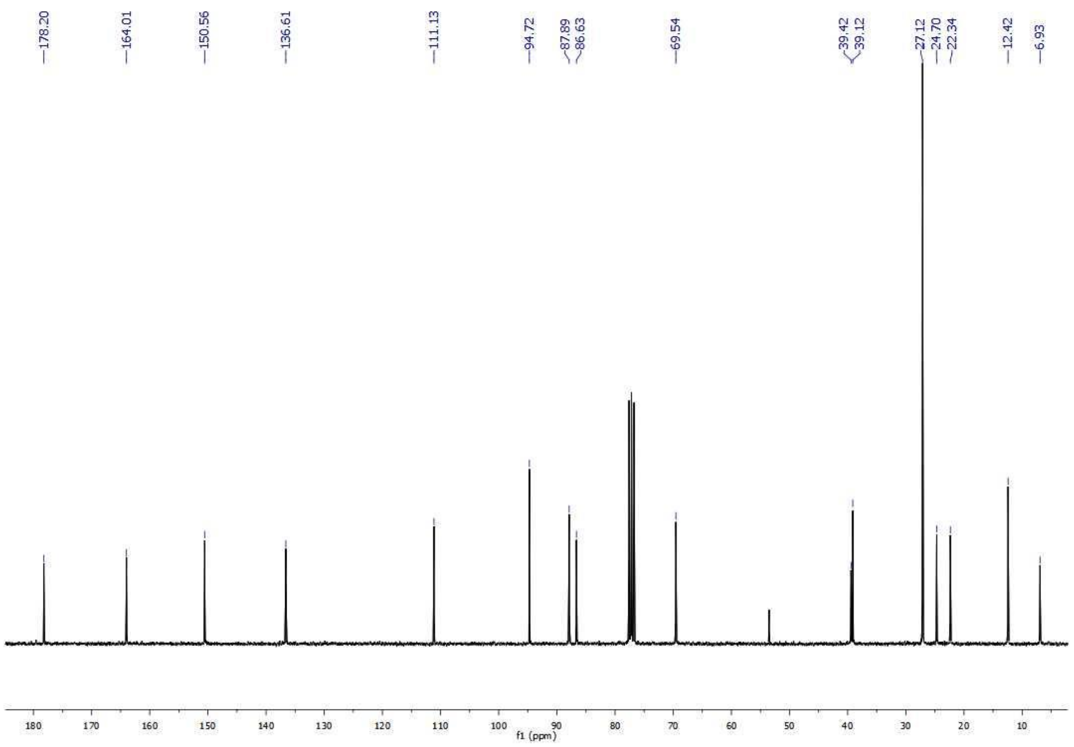

Figure S8: ^{1}H ($CDCl_3$, 400 MHz) and ^{13}C NMR ($CDCl_3$, 75 MHz) spectrum of **7β**

(5'*R*)-(2'-Deoxy-3',5'-ethano-6',7'-methano-β-D-ribofuranosyl)thymine (8). The solution of **7β** (0.235 g, 0.64 mmol) in dioxane (3 mL) was treated with Bu_4NOH (4.3 mL, 6.45 mmol) at ambient temperature for 16 h. The mixture was diluted with 10 mL of EtOAc/EtOH (9:1) silica-gel was added and all volatiles were removed in vacuo. The product was isolated by CC (EtOAc/EtOH 9:1) to afford title compound **8 (**171 mg, 94%) as a white solid.

*Data for **8***: R_f = 0.47 (EtOAc/EtOH 9:1); ^{1}H NMR (CD_3OD, 400 MHz) δ 8.32 (*q*, 1H, *J* = 1.2 Hz H-C(6)), 6.45 (*dd*, *J* = 10.1, 4.9 Hz, 1H, H-C(1')), 4.17 (*d*, *J* = 5.4 Hz, 1H, H-C(5')), 3.85 (*d*, *J* = 5.3 Hz, 1H, H-C(4')), 2.32 (*dd*, *J* = 12.6, 4.9 Hz, 1H), 2.24 (*dd*, *J* = 12.6, 10.2 Hz, 1H, H-C(2')), 1.88 (*d*, *J* = 1.1 Hz, 3H, CH_3-C(5)), 1.74 ((*ddd*, *J* = 8.1, 5.7, 3.8 Hz, H-C(7')), 1.59 ((*ddd*, *J* = 8.9, 5.6, 4.4 Hz H-C(6')), 0.69-0.63 (m, 1H, H-C(8')), 0.46 (dt, *J* = 5.8, 4.1 Hz, 1H, H-C(8')) ppm; ^{13}C NMR (CD_3OD, 75 MHz) δ166.4 (*s*, C(4)), 152.5 (*s*, C(2)), 139.2 (*d*, C(6)), 111.2 (*s*, C(5)), 90.3 (*d*, C(4')), 89.1 (*s*, C(3')), 88.5 (*d*, C(1')), 71.7 (*d*, C(5')), 44.7 (*t*, C(2')), 24.7, 24.6 (2×*d*, C(6'), C(7')), 12.5 (*q*, CH_3-C(5)), 7.5 (*t*, C(9')) ppm; ESI^+-HRMS: m/z calcd for $C_{13}H_{17}N_2O_5$ $[M+H]^+$ 281.1137, found 281.1138.

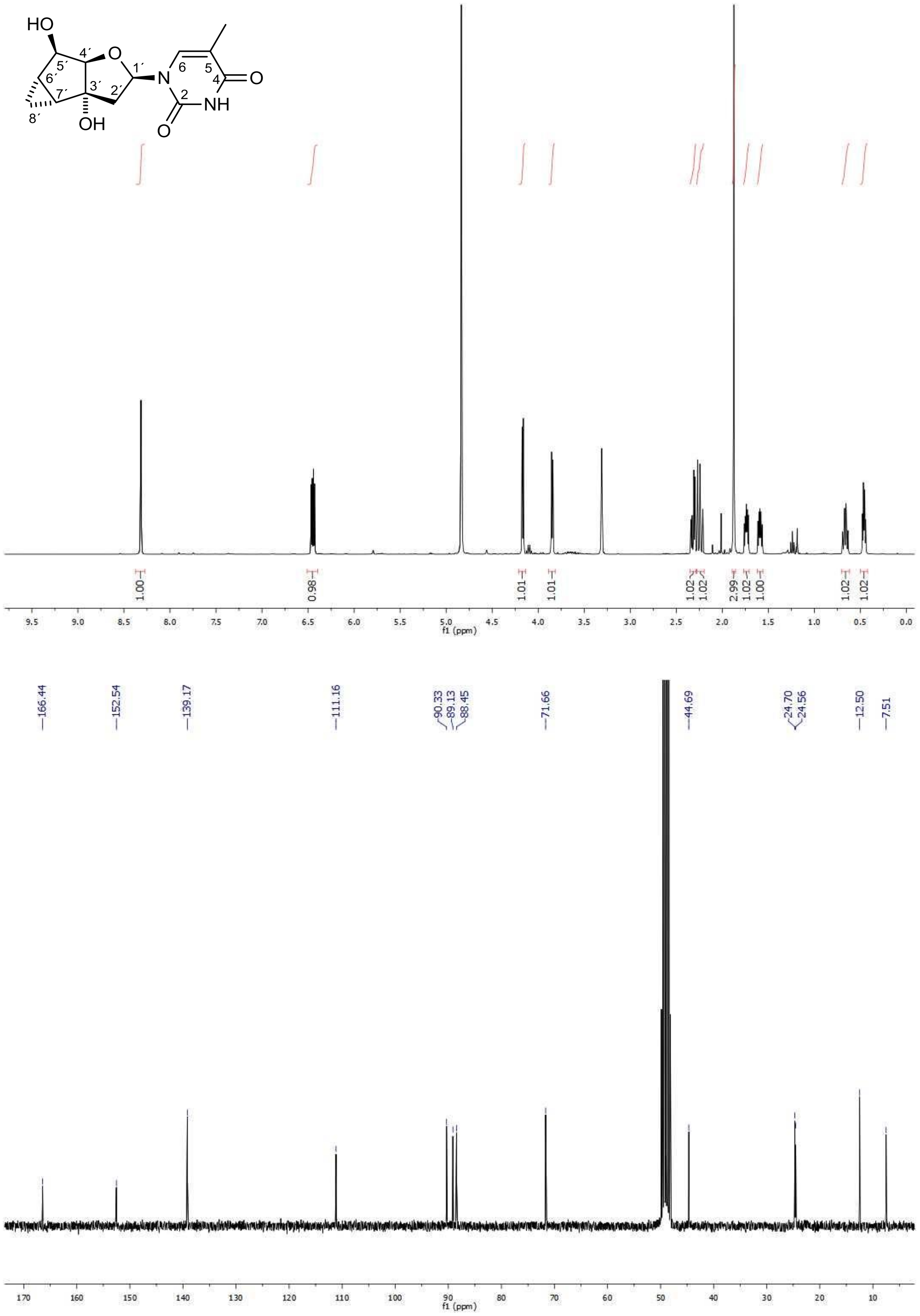

Figure S9: ^{1}H (CD_3OD, 400 MHz) and ^{13}C NMR (CD_3OD, 75 MHz) spectrum of **8**

(5’*R*)-(5´-*O*-((4,4’-Dimethoxytriphenyl)methyl)-2´-deoxy-3´,5´-ethano-6’,7’-methano-β-D-ribofuranosyl)thymine (9). To a suspension of **8** (153 mg, 0.55 mmol) in mixture of CH_2Cl_2 (8 mL) dry

pyridine (720 µL) was added DMTrCl (590 mg, 1.74 mmol) in portions over 24 h and the reaction was stirred for additional 12 h at ambient temperature. The mixture was diluted with sat. aq. $NaHCO_3$, extracted with EtOAc and extracts dried over $MgSO_4$. The title compound **9** (312 mg, 98%) was isolated by CC (CH_2Cl_2/MeOH 95:5) as a white foam.

*Data for **9***: R_f = 0.62 (EtOAc/EtOH 9:1); ^{1}H NMR ($CDCl_3$, 300 MHz) δ 9.17 (*sbr*, 1H, NH), 7.97 (*d*, *J* = 1.1 Hz, 1H, H-C(6)), 7.50-7.46 (*m*, 2H), 7.40 – 7.27 (*m*, 7H), 6.87- 6.81 (*m*, 4H, H-DMTr), 6.50 (*dd*, *J* = 10.0, 4.2 Hz, 1H, H-C(1’)), 4.27 (*d*, *J* = 6.1 Hz,1H), 4.02 (*d*, *J* = 6.2 Hz,1H, H-C(5’,4’)), 3.80, 3.79 (2×*s*, 6H, H-C(OCH_3)), 3.05 (*s*, 1H, OH), 2.60 (*dd*, *J* = 12.3, 4.2 Hz, 1H), 2.34 (*dd*, *J* = 12.2, 10.1 Hz, 1H, H-C(2’)), 1.61- 1.55 (*m*, 1H, H-C(7’)), 0.81 (*d*, *J* = 0.8 Hz, 3H, CH_3-C(5)), 0.38-0.17 (*m*, 3H, H-C(6’,8’)); ^{13}C NMR ($CDCl_3$, 75 MHz) δ 164.1 (*s*, C(4)), 159.1 (*s*, DMTr), 150.7 (*s*, C(2)), 144.6 (*s*, DMTr), 137.0 (*d*, C(6)), 136.1, 136.0 (2×*s*, DMTr), 130.9, 129.2, 128.1, 127.6, 113.3 (6×*d*, DMTr), 111.1 (*s*, C(5)), 89.2 (*d*, C(1’/5’)), 88.7, 88.1 (2×*s*, C(3’), C-DMTr), 87.9 (*d*, C(1’/5’)), 73.2 (*d*, C(4’)), 55.4 (*q*, OCH_3), 44.0 (*t*, C(2’)), 24.0, 23.3 (2×*d*, C(6’), C(7’)), 10.8 (*q*, CH_3-C(5)), 7.4 (*t*, C(9’)) ppm; ESI$^+$-HRMS: m/z calcd for $C_{34}H_{34}N_2NaO_7$ [*M*+Na]$^+$ 605.2264, found 605.2268.

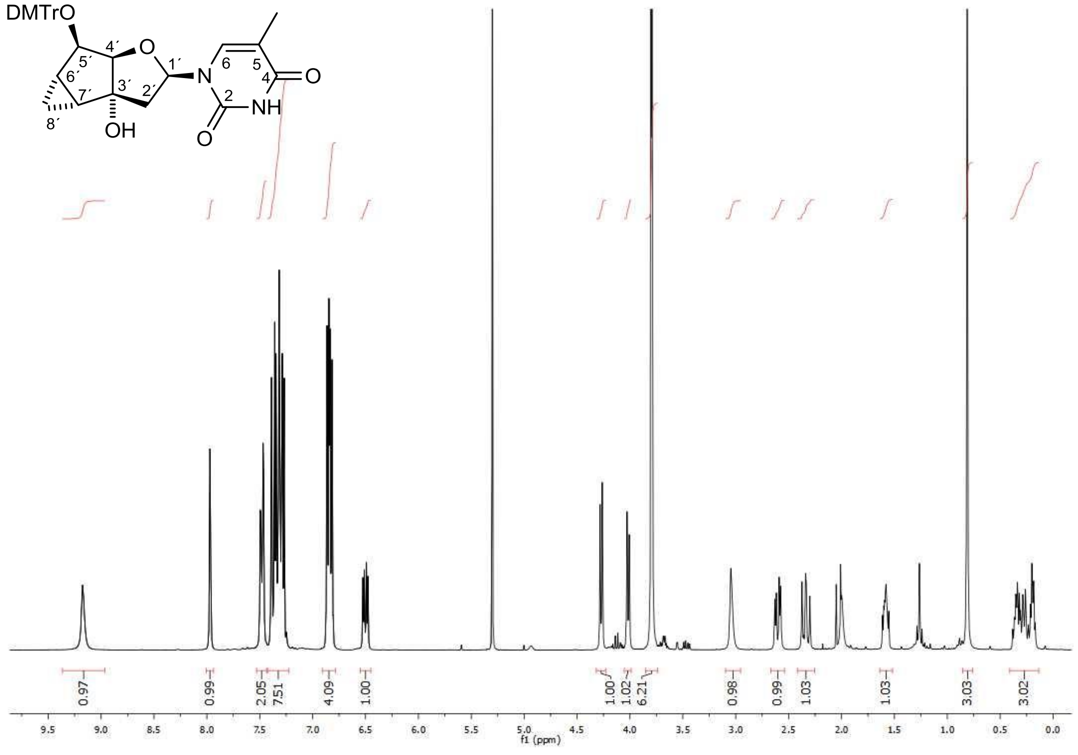

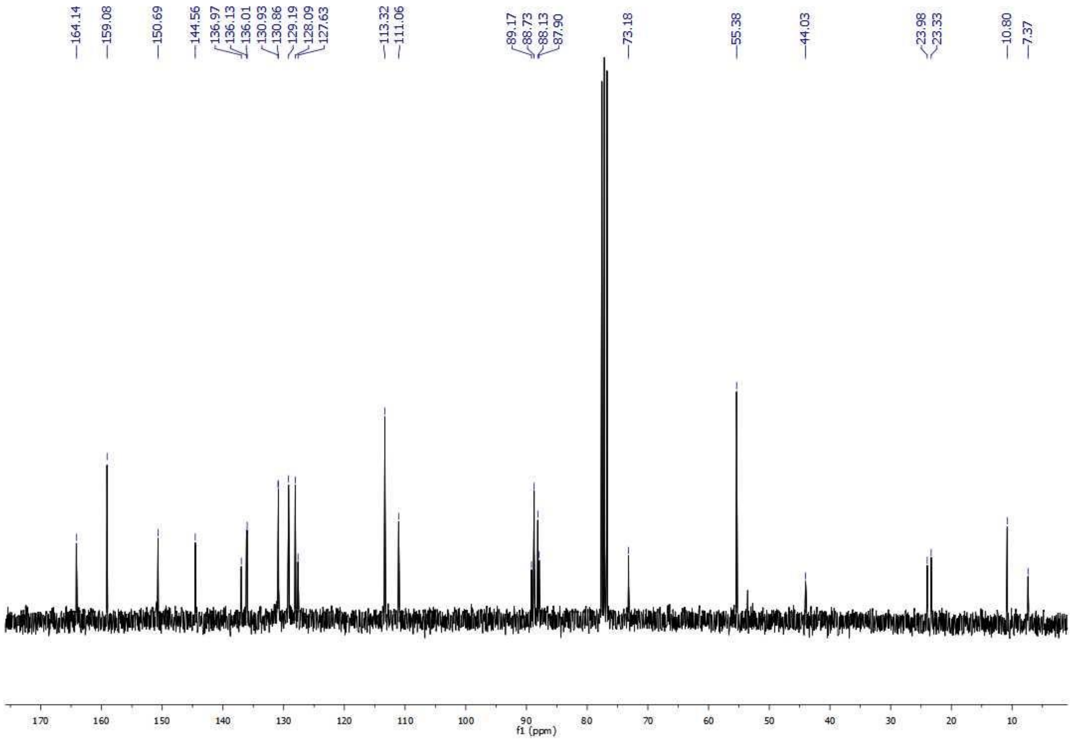

Figure S10: ^{1}H ($CDCl_3$, 300 MHz) and ^{13}C NMR ($CDCl_3$, 75 MHz) spectrum of **9**

(5'*R*)-(5´-*O*-((4,4'-Dimethoxytriphenyl)methyl)-3'-*O*-((2-cyanoethoxy)(diisopropylamino)phosphanyl)-2´-deoxy-3´,5´-ethano-6',7'-methano-β-D-ribofuranosyl)thymine (10).

To a solution of **9** (312 mg, 0.54 mmol) and *N*-ethyldiisopropylamine (0.55 mL, 3.21 mmol) in dry THF (5 mL) was added 2-cyanoethyl *N*,*N*-diisopropylchlorophosphoramidite (0.36 mL, 1.61 mmol) at rt. After the solution had been stirred for 4 h, the mixture was poured into sat. aq. $NaHCO_3$, extracted with EtOAc and extracts were dried over $MgSO_4$. After filtration and evaporation, the residual oil was purified by CC (EtOAc/Hexane 7:3) to give the title compound **10** (373 mg, 89%) as a white foam.

*Data for **10***: R_f = 0.66, 0.51 (EtOAc/Hex 7:3); ^{1}H NMR ($CDCl_3$, 400 MHz) δ 8.99 (*sbr*, 1H, NH), 7.96-7.94 (*m*, 1H, H-C(6)), 7.50-7.47 (*m*, 2H), 7.40-7.26 (*m*, 7H), 6.87-6.82 (*m*, 4H, H-DMTr), 6.48 (*dd*, J = 10.2, 4.0 Hz, 1H, H-C(1')), 4.27-4.25 (*m*, 1H), 4.17-4.10 (*m*, 1H, H-C(4'), H-C(5')), 3.93-3.58 (*m*, 10H, CH-^{i}Pr, OCH_3, OCH_2), 2.92-2.58 (*m*, 3H, H-C(2'), CH_2CN), 2.36-2.26 (*m*, 1H, H-C(2')), 1.81-1.73 (*m*, 1H, H-C(6'/7')), 1.27-1.16 (*m*, 12H, CH_3-^{i}Pr), 0.77 (*s*, 3H, CH_3-C(5)), 0.45-0.31 (*m*, 2H), 0.27-0.19 (*m*, 1H, H-C(6'), H-C(7'), H-C(8')); ^{13}C NMR ($CDCl_3$, 75 MHz) δ 164.1 (*s*, C(4)), 159.1 (*s*, DMTr), 150.7, 150.4 (2×*s*, C(2)), 144.5 (*s*, DMTr), 137.0, 136.9 (2×*d*, C(6)), 136.1, 136.0, 135.9 (4×*s*, DMTr), 131.0, 130.9, 129.2, 128.1, 127.6 (6×*d*, DMTr), 118.5, 117.8 (2×*s*, CN), 113.3 (*d*, DMTr), 111.0, 110.8 (2×*s*, C(5)), 93.3 (*dd*, $^{2}J(C,P)$ = 12 Hz, C(3`)), 92.8 (*dd*, $^{2}J(C,P)$ = 10 Hz, C(3`)), 89.3 (*dd*, $^{3}J(C,P)$ = 5 Hz, C(4')), 89.2 (*dd*, $^{3}J(C,P)$ = 4 Hz, C(4')), 88.2 (2×*s*, C-DMTr), 87.7, 87.6 (2×*d*, C(1')), 72.3, 72.1 (2×*d*, C(5')), 58.1, 57.9 (2×*td*, $^{2}J(C,P)$ = 18 Hz, OCH_2), 55.4 (*q*, OCH_3), 43.6, 43.3 (2×*dd*, $^{2}J(C,P)$ = 13 Hz ,CH-^{i}Pr), 24.8-24.4, 22.6-22.2(2×m, CH_3-^{i}Pr, C(6'),C(7')), 20.5 (2×*td*, $^{3}J(C,P)$ = 8 Hz, CH_2CN), 10.7, 10.6 (2×*q*, CH_3-C(5)), 8.7 (*t*, C(8')); ^{31}P NMR ($CDCl_3$, 122 MHz) δ 143.0, 142.3; ESI^{+}-HRMS: m/z calcd for $C_{43}H_{51}N_4NaO_8P$ [*M*+Na]$^{+}$ 805.3342, found 805.3354.

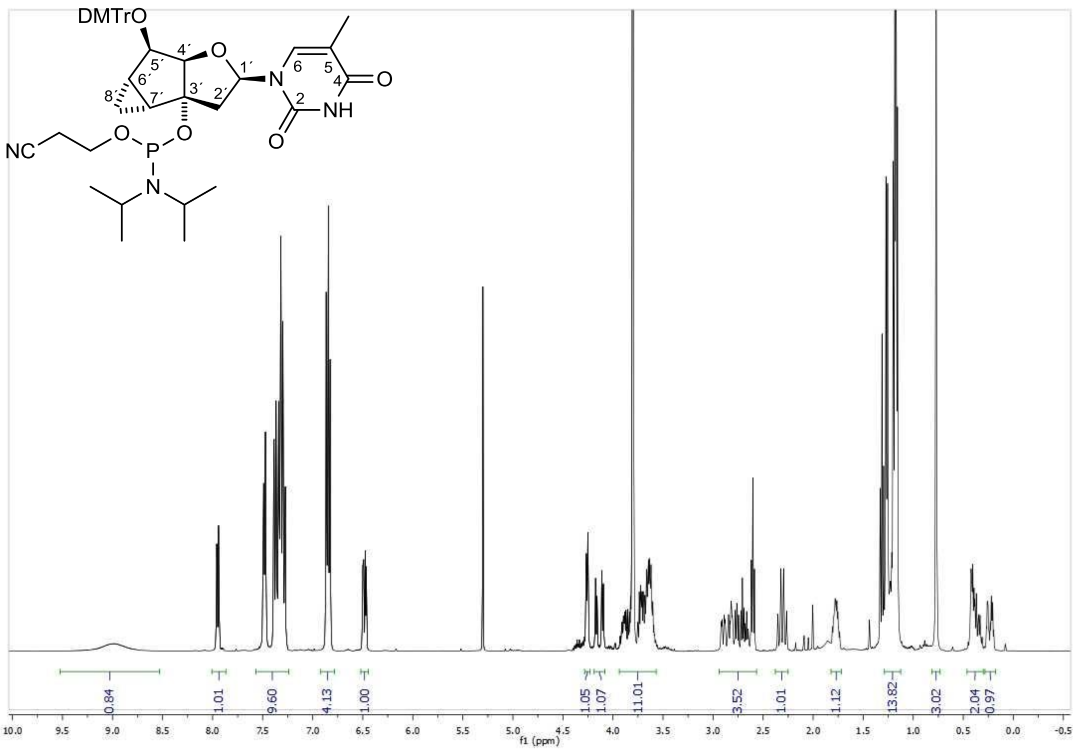
DMTrO
NC
P
N
NH
O
4´
5´
6´
7´
8´
3´
2´
1´
6
5
4
2
0.84
1.01
9.60
4.13
1.00
1.05
1.07
11.01
3.52
1.01
1.12
13.82
3.02
2.04
0.97
f1 (ppm)

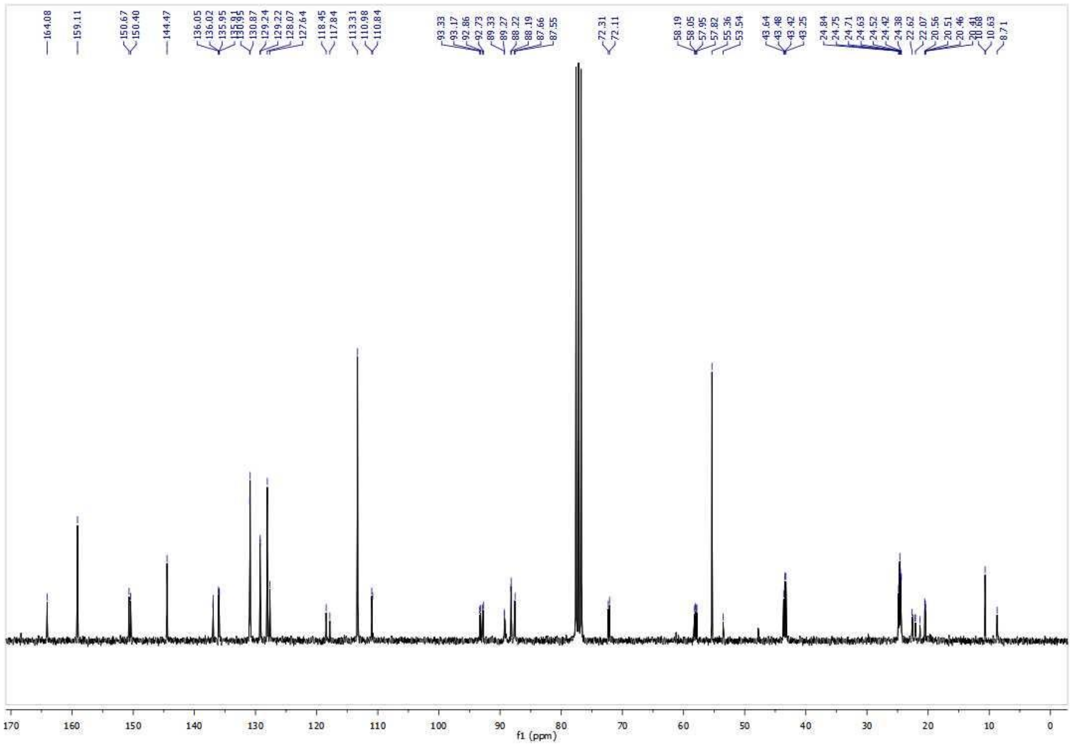
164.08
159.11
150.67
150.40
144.47
136.05
136.02
135.95
130.87
129.24
129.22
128.07
127.64
118.45
117.84
113.31
110.98
110.84
93.33
93.17
92.86
92.73
89.33
89.27
88.22
88.19
87.66
87.55
72.31
72.11
58.19
58.05
57.95
57.92
55.36
53.54
43.64
43.48
43.42
43.25
24.84
24.75
24.71
24.63
24.52
24.42
24.38
22.62
22.07
20.56
20.51
20.46
10.63
8.71
f1 (ppm)

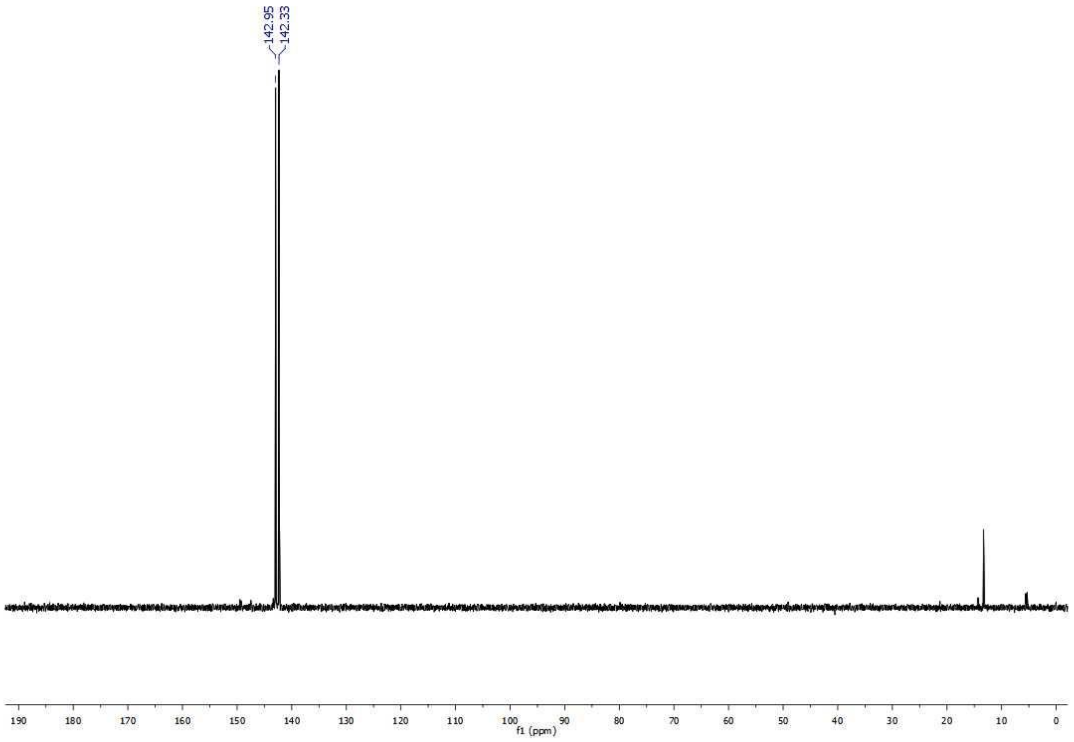

Figure S11: ^{1}H ($CDCl_3$, 400 MHz), ^{13}C ($CDCl_3$, 75 MHz) and ^{31}P NMR ($CDCl_3$, 122 MHz) spectrum of **10**

(2´-Deoxy-3´,5´-etheno-α-and-β-D-ribofuranosyl)thymine (11 α,β). Into stirred ice-cooled suspension of thymine (0.494 g, 3.92 mmol) and **2** (0.270 g, 1.57 mmol) in MeCN (16 mL) under the atmosphere of Ar were added BSA (2.3 mL, 9.41 mmol) and TMSCl (0.05 mL, 0.63 mmol) successively and the mixture was stirred until clear solution appeared (40 min). Then the TMSOTf (1.42 mL, 7.84 mmol) was added drop wise at 0 °C, cooling bath was removed and the reaction mixture was stirred at ambient temperature for 2.5 h. The reaction was quenched by addition of sat. aq. $NaHCO_3$ (5.5 mL), silica gel was added and all liquids were evaporated. The mixture was chromatographed to afford 0.906 g of crude product containing inseparable mixture of **11 α,β** which was used without further treatment for the subsequent step. Compounds **11 α and 11β** (white solids) for analyses were obtained by preparative TLC (8% MeOH in CH_2Cl_2).

*data for **11β**:* R_f (8% MeOH in CH_2Cl_2) = 0.16; ^{1}H NMR (CD_3OD, 300 MHz): δ = 7.93 (q, *J* = 1.2 Hz, 1H; H-C(6)), 6.43 (dd, *J* = 8.0, 6.1 Hz, 1H; H-C(1`)), 5.93 (dd, *J* = 5.8, 1.1 Hz, 1H), 5.86 (dd, *J* = 5.8, 2.0 Hz, 1H; H-C(6`,7`)), 4.80 (ddd, *J* = 5.7, 2.0, 1.1 Hz, 1H; H-C(5`)), 4.21 (d, *J* = 5.7 Hz, 1H; H-C(4`)), 2.48 (dd, *J* = 13.6, 6.1 Hz, 1H; H-C(2`)), 2.13 (dd, *J* = 13.6, 8.0 Hz, 1H; H-C(2`)), 1.86 (d, *J* = 1.2 Hz, 1H; $CH_3$) ppm; $^{13}C$ NMR ($CD_3OD$, 75 MHz): δ = 166.6 (s, C(4)), 152.5 (s, C(2)), 138.8 (d, C(6)), 137.7, 135.5 (2×d, C(6`, 7`)), 111.4 (s C(5)), 91.3 (s, C(3`)), 89.3, 89.2 (2×d, C(1`, 5`)), 75.2 (d, C(4`)), 46.3 (t, C(2`)), 12.6 (q, CH_3) ppm.

*data for **11α**:* R_f (8% MeOH in CH_2Cl_2) = 0.16; ^{1}H NMR (CD_3OD, 300 MHz): δ = 7.74 (q, *J* = 1.2 Hz, 1H; H-C(6)), 6.22 (t, *J* = 6.6 Hz, 1H; H-C(1`)), 5.95 (dd, *J* = 5.9, 0.9 Hz, 1H), 5.91 (ddd, *J* = 5.9, 1.7, 0.5 Hz, 1H; H-C(6`,7`)), 4.77 (ddd, *J* = 5.3, 1.6, 1.2 Hz, 1H; H-C(5`)), 4.44 (d, *J* = 5.3 Hz, 1H; H-C(4`)), 2.57 (dd, *J* = 13.7, 6.6

Hz, 1H; H-C(2`)), 2.26 (dd, *J* = 13.7, 6.5 Hz, 1H; H-C(2`)), 1.91 (d, *J* = 1.2 Hz, 3H, H-CH_3) ppm; ^{13}C NMR (CD_3OD, 75 MHz): δ = 166. 6 (s, C(4)), 152.5 (s, C(2)), 138.3 (d, C(6)), 137.2, 136.8 (2×d, C(6`, 7`)), 111.9 (s, C(5)), 90.7 (s, C(3`)), 89.1 (2×d, C(1`, 4`)), 76.2 (d, C(5`)), 45.7 (t, C(2`)), 12.6 (q, CH_3) ppm.

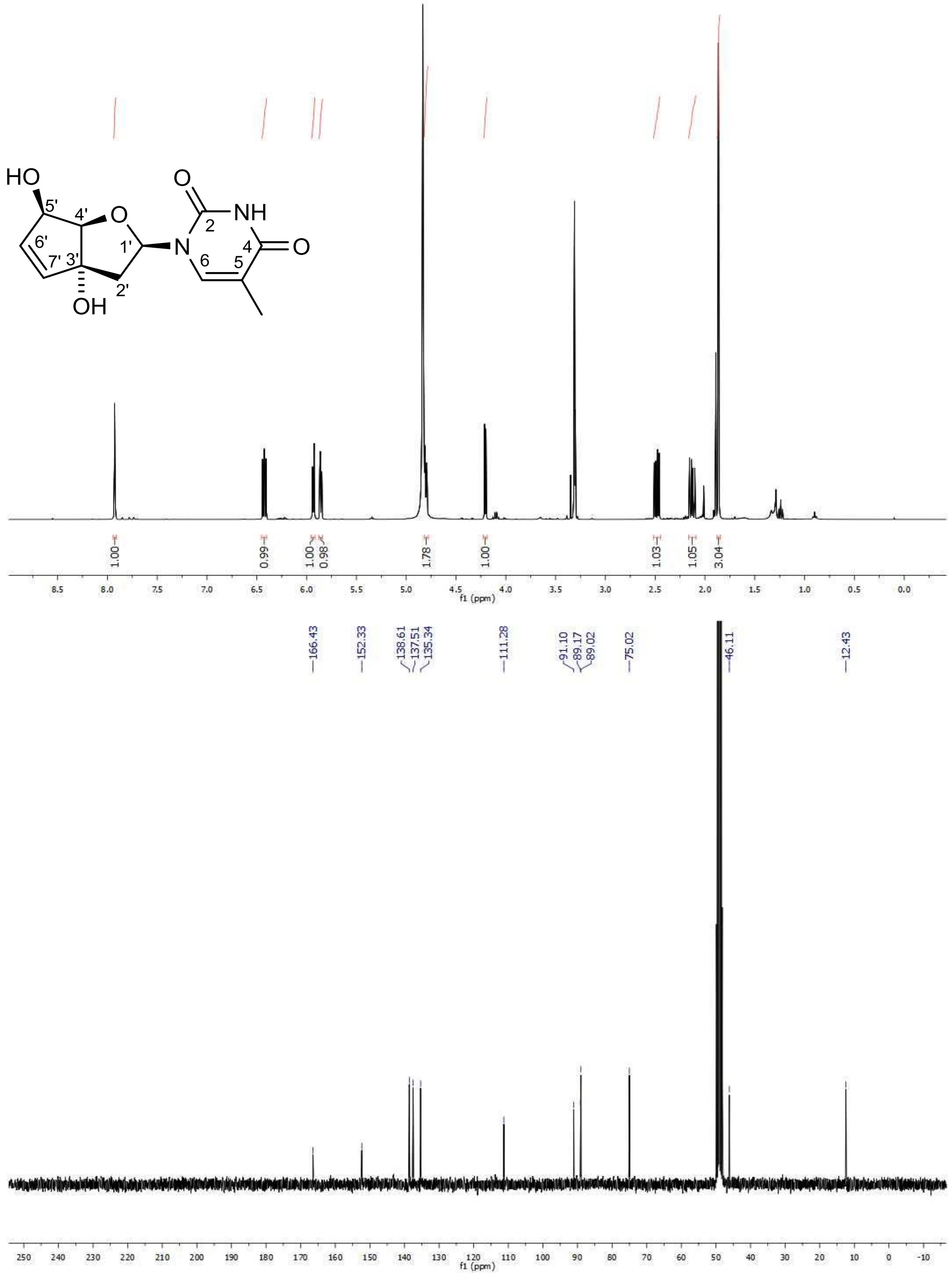

Figure S12: ^{1}H (CD_3OD, 300 MHz), ^{13}C NMR (CD_3OD, 75 MHz) spectrum of **11β**

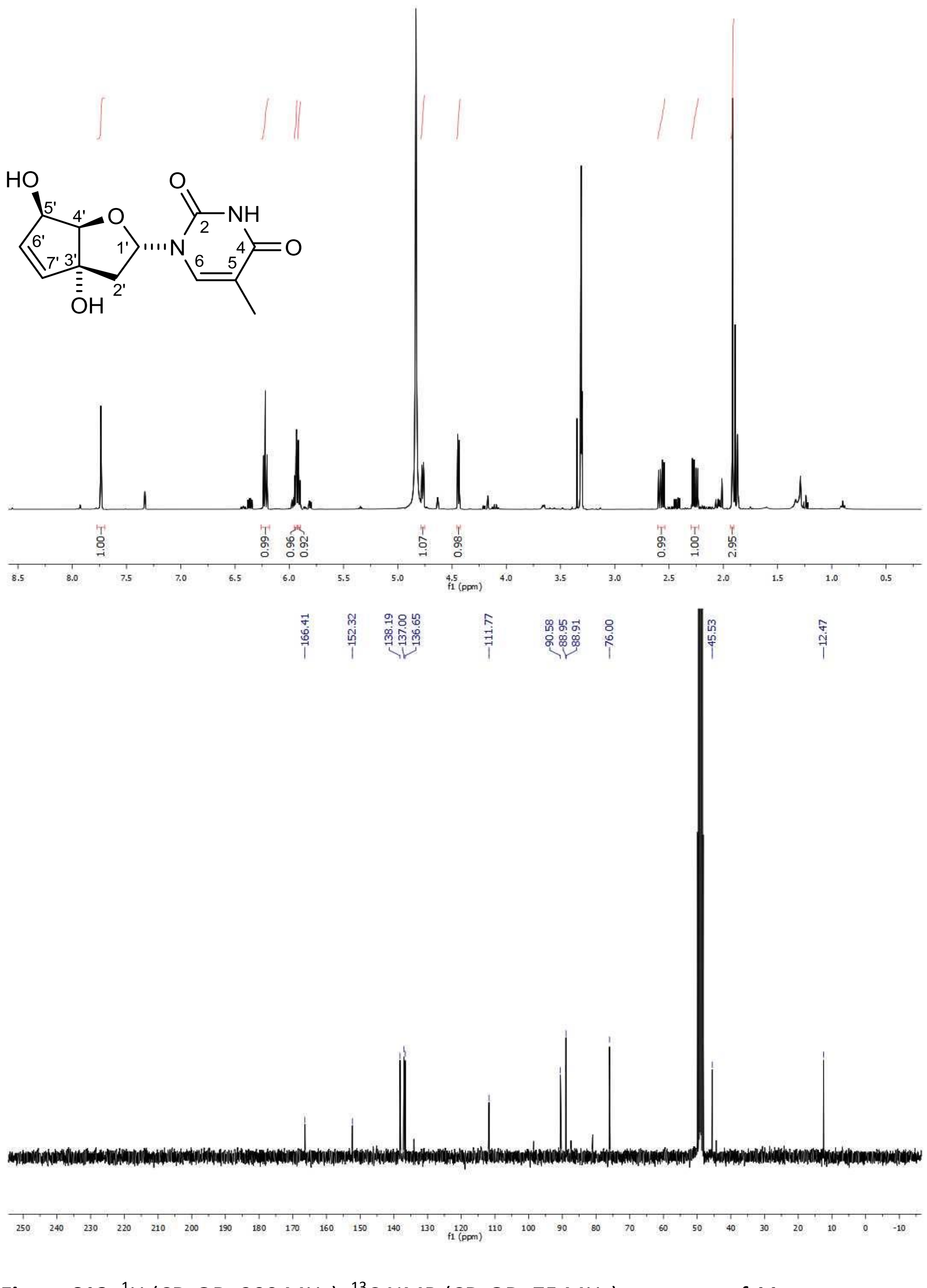

Figure S13: ^{1}H (CD_3OD, 300 MHz), ^{13}C NMR (CD_3OD, 75 MHz) spectrum of **11α**

[5´-*O*-((4,4´-Dimethoxytriphenyl)methyl)-2´-deoxy-3´,5´-etheno-α-and-β-D-ribofuranosyl]thymine (12α,β). The crude mixture of **11α,β** (0.906 g, containing max. 1.57 mmol of **11α,β**) was dissolved in dry pyridine (15 mL) and DMTCl (1.06 g, 3.14 mmol) was added in one portion. The solution was stirred overnight at RT under atmosphere of Ar. It was poured into sat. aq. $NaHCO_3$ and extracted with EtOAc. Extracts were dried with $MgSO_4$ and after evaporation chromatographed (silica gel, 3.5% MeOH in CH_2Cl_2) to provide 0.262 g of **12α** (29%, pale yellow foam) and 0.307 g of **12β** (34%, pale yellow foam).

*data for **12β**:* R_f (8% MeOH in CH_2Cl_2) = 0.33; ^{1}H NMR ($CDCl_3$, 400 MHz): δ = 8.95 (s, 1H; NH), 7.96 (q, *J* = 1.2 Hz, 1H; H-C(6)), 7.54-7.48 (m, 2H), 7.46-7.36 (m, 4H), 7.31-7.17 (m, 3H), 6.87-6.78 (m, 4H; Ph), 6.18 (dd, *J* = 6.6, 4.0 Hz, 1H; H-C(1`)), 5.53 (dd, *J* = 5.7, 1.6 Hz, 1H; H-C(7`)), 4.80 (dt, *J* = 5.3, 1.6 Hz, 1H; H-C(5`)), 4.69 (ddd, *J* = 5.6, 1.6, 0.8 Hz, 1H; H-C(6`)), 4.16 (d, *J* = 5.3 Hz, 1H; H-C(4`)), 3.77 (2×s, 2×3H; OMe), 2.71 (dd, *J* = 14.1, 6.6 Hz, 1H; H-C(2`)), 2.47 (s, 1H; OH), 2.31 (dd, *J* = 14.1, 4.0 Hz, 1H; H-C(2`)), 1.66 (d, *J* = 1.2 Hz, 3H; H-$CH_3$) ppm; $^{13}$C NMR ($CDCl_3$, 100 MHz): δ = 164.3 (s, C(4)), 159.0 (s, C-Ph), 150.6 (s, C(2)), 145.3 (s, C-Ph), 137.3 (d, C(6)), 136.6 (d, C(6`)), 136.4 (2×s, C-Ph), 134.6 (d, C(7`)), 130.3, 128.3, 127.3, 113.6 (7×d, C-Ph), 109.6 (s C(5)), 89.5 (s, C(3`)), 88.6 (d, C(1`)), 88.2 (d, C(4`)), 88.1 (s, C(CPh_3)), 77.5 (d, C(5`)), 55.5 (q, $OCH_3$), 45.9 (t, C(2`)), 12.4 (q, CH_3) ppm; ESI$^+$-HRMS: calcd for $C_{33}H_{32}N_2NaO_7$: 591.2102 [*M*+Na]$^+$; found: 591.2121.

*data for **12α**:* R_f (8% MeOH in CH_2Cl_2) = 0.33; ^{1}H NMR ($CDCl_3$, 400 MHz): δ = 8.85 (s, 1H; NH), 7.51-7.48 (m, 2H), 7.43-7.34 (m, 4H), 7.32-7.22 (m, 3H; H-Ph, H-C(6)), 7.23-7.15 (m, 1H), 6.85-6.76 (m, 4H; Ph), 6.26 (dd, *J* = 7.3, 5.4 Hz, 1H; H-C(1`)), 5.70 (dd, *J* = 5.9, 1.0 Hz, 1H; H-C(7`)), 4.73 (dd, *J* = 5.9, 1.9 Hz, 1H; H-C(6`)), 4.62 (ddd, *J* = 5.8, 1.9, 1.2 Hz, 1H; H-C(5`)), 4.16 (d, *J* = 5.8 Hz, 1H; H-C(4`)), 3.77 (s, 6H; OMe), 3.02 (s, 1H; OH), 2.67 (dd, *J* = 14.1, 7.3 Hz, 1H; H-C(2`)), 2.30 (dd, *J* = 14.1, 5.4 Hz, 1H; H-C(2`)), 1.94 (d, *J* = 1.1 Hz, 3H; H-$CH_3$) ppm; $^{13}$C NMR ($CDCl_3$, 100 MHz): δ = 164.0 (s, C(4)), 158.9 (s, C-Ph), 150.5 (s, C(2)), 145.5 (s, C-Ph), 137.7 (d, C(6)), 137.0, 136.8 (2×s, C-Ph), 135.5, 135.4 (2×d, C(6`, 7`)), 130.5, 128.5, 128.1, 127.1, 113.4 (7×d, C-Ph), 111.1 (s C(5)), 90.3, 90.3 (s, d, C(1`, 3`)), 88.8 (d, C(4`)), 87.6 (s, CPh_3), 76.7 (d, C(5`)), 55.5 (q, $OCH_3$), 45.1 (t, C(2`)), 12.7 (q, CH_3) ppm; ESI$^+$-HRMS: calcd for $C_{33}H_{32}N_2NaO_7$: 591.2102 [*M*+Na]$^+$; found: 591.2122.

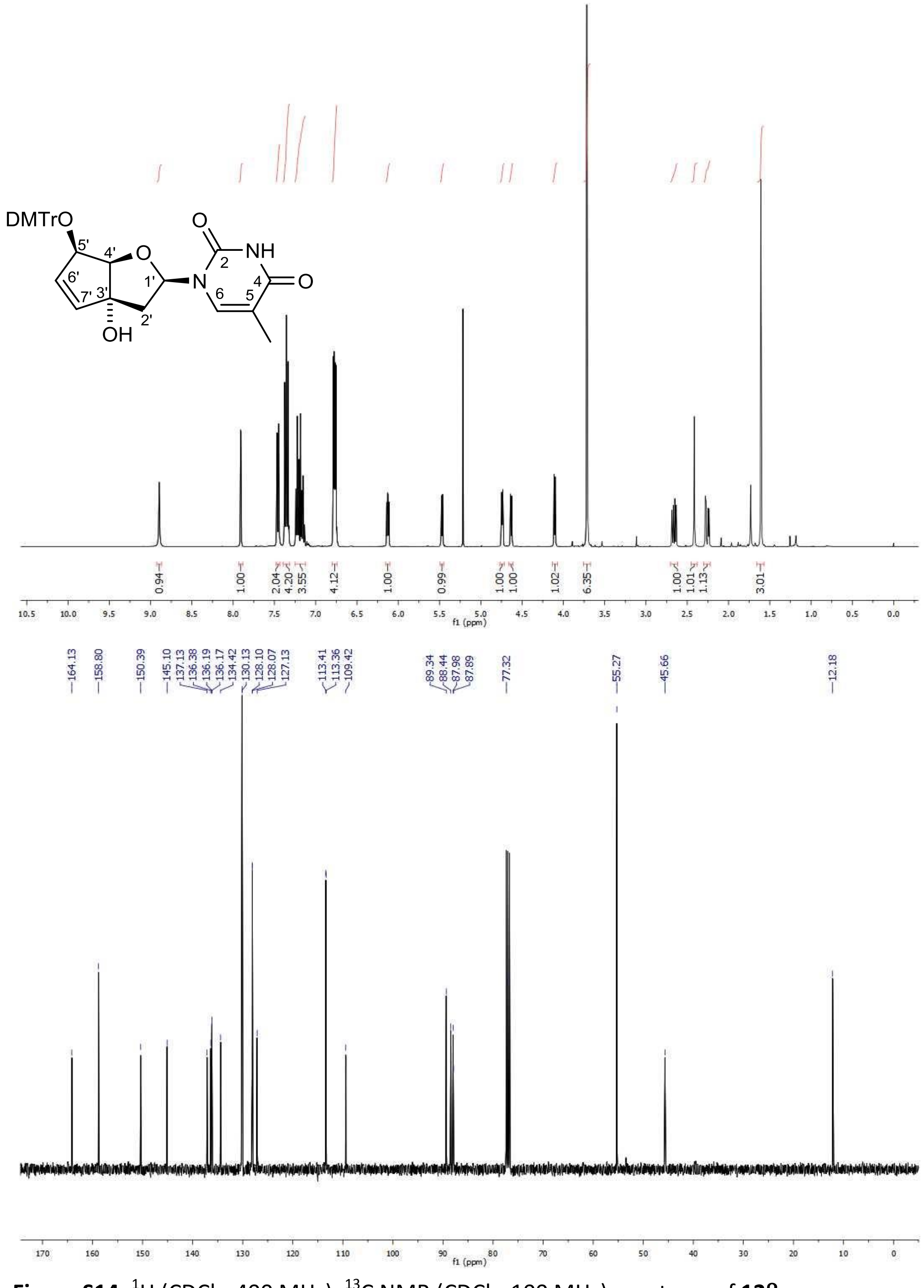

Figure S14: ^{1}H ($CDCl_3$, 400 MHz), ^{13}C NMR ($CDCl_3$, 100 MHz) spectrum of **12β**

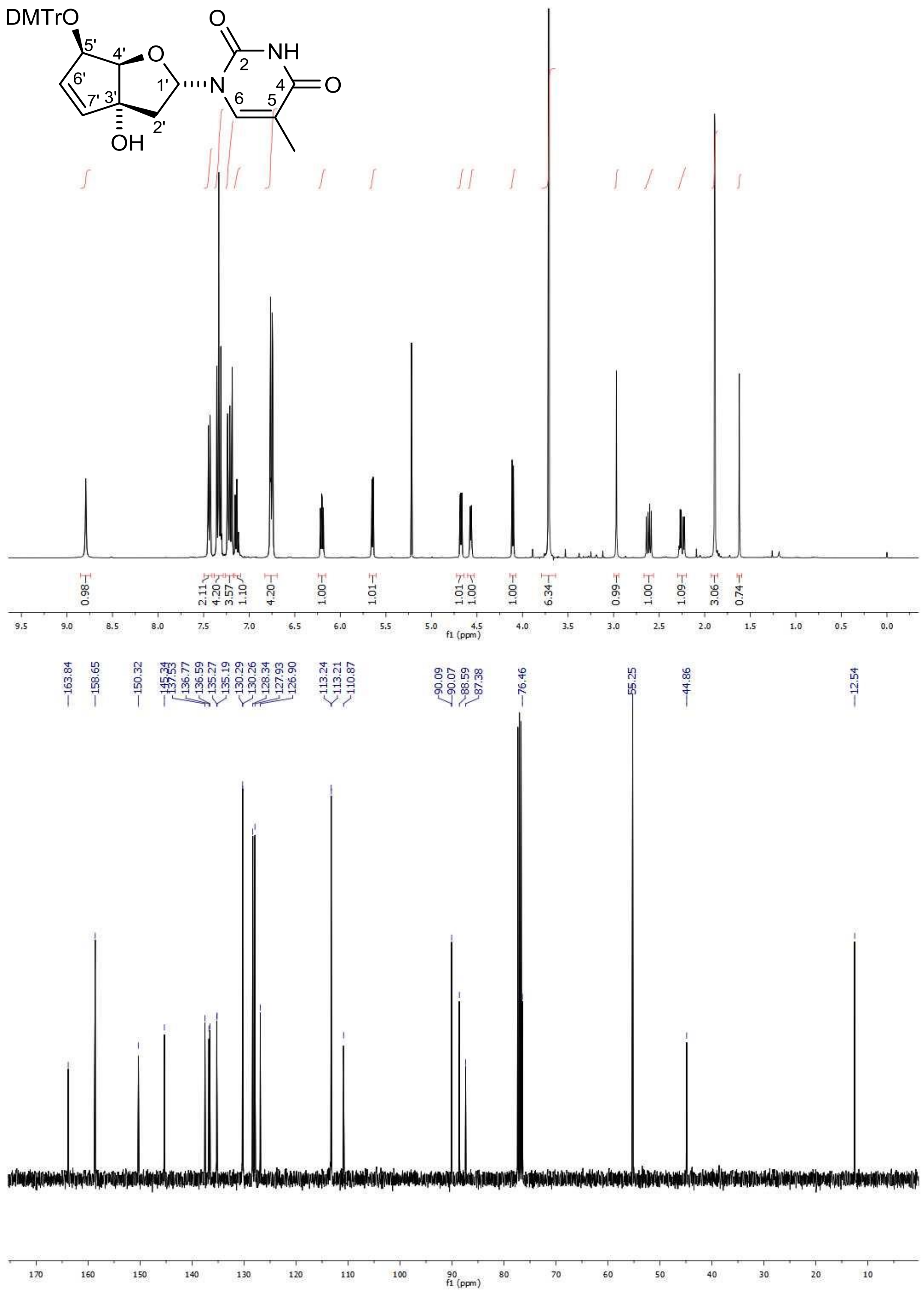

Figure S15: ^{1}H ($CDCl_3$, 400 MHz), ^{13}C NMR ($CDCl_3$, 100 MHz) spectrum of **12α**

{3´-*O*-[(2-Cyanoethoxy)(diisopropylamino)phosphanyl]-5´-*O*-(4,4´-dimethoxytriphenyl)methyl-2´-deoxy-3´,5´-etheno-β-D-ribofuranosyl}thymine (13). 2-Cyanoethyl *N*,*N*-diisopropylchlorophosphoramidite (CEPCl, 0.34 mL, 1.51 mmol) was added at RT into a solution of

nucleoside **12β** (0.286 g, 0.50 mmol) and DIPEA (0.52 mL, 3.02 mmol) in dry THF (5 mL). After stirring for 1 h under an Ar atmosphere, the mixture was poured into sat. aq. $NaHCO_3$ and extracted with Et_2O. Extracts were dried with $MgSO_4$ and after evaporation of solvents the remaining crude was purified by CC (EtOAc/hexane 7:3) to provide title compound **13** (0.355 g, 94%) as a white foam.

*data for **13**:* R_f (7:3 EtOAc/hexane) = 0.57, 0.43; ^{1}H NMR ($CDCl_3$, 400 MHz): δ = 8.83 (brs, 1H; NH), 7.98 (q, *J* = 1.2 Hz, 0.4H; H-C(6)), 7.91 (q, *J* = 1.2 Hz, 0.6H; H-C(6)), 7.56-7.47 (m, 2H), 7.46-7.34 (m, 4H), 7.36-7.16 (m, 3H), 6.89-6.77 (m, 4H; H-Ph), 6.18 (dd, *J* = 6.6, 4.3 Hz, 0.6H; H-C(1`)), 6.13 (dd, *J* = 6.7, 3.6 Hz, 0.4H; H-C(1`)), 5.71 (d, *J* = 5.7 Hz, 0.6H; H-C(7`)), 5.64 (dt, *J* = 5.5, 1.4 Hz, 0.4H; H-C(7`)), 4.78 (d, *J* = 5.9 Hz, 0.6H; H-C(6`)), 4.76 – 4.69 (m, 1.4H; H-C(6`, 5`)), 4.28 (d, *J* = 5.1 Hz, 0.4H; H-C(4`)), 4.23 (d, *J* = 5.3 Hz, 0.6H; H-C(4`)), 3.79, 3.78, 3.77 (4×s, 6H; OMe), 3.71-3.43 (m, 4H; H-C(*i*-Pr), $OCH_2$), 2.89-2.84 (m, 1H; H-C(2`)), 2.55-2.49 (m, 1H; -CH_2CN), 2.42-2.33 (m, 1H; H-C(2`)), 2.34-2.18 (m, 1H; CH_2CN), 1.69 (d, *J* = 1.2 Hz, 1.3H; H-CH_3), 1.66 (d, *J* = 1.2 Hz, 1.8H; H-CH_3), 1.11 – 1.05 (m, 9H; $CH(CH_3)_2$), 1.01 (d, *J* = 6.8 Hz, 3H; $CH(CH_3)_2$) ppm; ^{13}C NMR ($CDCl_3$, 100 MHz): δ = 164.3, 164.2 (2×s, C(4)), 159.0 (2×s, C-Ph), 150.5 (s, C(2)), 145.3 (2×s, C-Ph), 137.3, 137.1 (2×d, C(6)), 136.8 (d, C(7`)), 136.5 (s, C-Ph), 136.4 (d, C(6`)), 136.3 (s, C-Ph), 134.4 (dd, $^3J(C,P)$ = 7 Hz, C(7`)), 134.0 (dd, $^3J(C,P)$ = 8 Hz, C(7`)), 130.4, 130.3, 128.3, 128.2, 127.4, 127.3 (9×d, C-Ph), 117.7, 117.4 (2×s, CN), 113.7, 113.6, 113.5 (4×d, C-Ph), 109.7, 109.4 (2×s C(5)), 92.4 (d, $^2J(C,P)$ = 11 Hz, C(3`)), 92.5 d, $^2J(C,P)$ = 10 Hz, C(3`)), 88.5 (2×d, C(1`)), 88.1 (2×s, $C(CPh_3)$), 87.8, 87.7 (2×d, C(4`)), 77.6 (dd, $^4J(C,P)$ = 1 Hz, C(5`)), 77.3 (dd, $^4J(C,P)$ = 3 Hz, C(5`)), 58.2 (dt, $^2J(C,P)$ = 18 Hz, OCH_2), 58.0 (dt, $^2J(C,P)$ = 17 Hz, OCH_2), 55.5 (2×q, OCH_3), 46.2, 46.1 (2×dt, $^3J(C,P)$ = 6 Hz, C(2`)), 43.6, 43.5 (2×dd, $^2J(C,P)$ = 13 Hz, $CH(CH_3)_2$), 24.7, 24.5, 24.4 (3×dq, $^3J(C,P)$ = 6 Hz, $CH(CH_3)_2$), 20.5, 20.0, (2×dt, $^3J(C,P)$ = 7 Hz, -CH_2CN), 12.4 (q, CH_3) ppm; ^{31}P NMR (122 MHz, $CDCl_3$): δ = 143.60, 142.99 ppm; ESI$^+$-HRMS: calcd for $C_{42}H_{49}N_4NaO_8P$: 791.3180 $[M+Na]^+$; found: 791.3160.

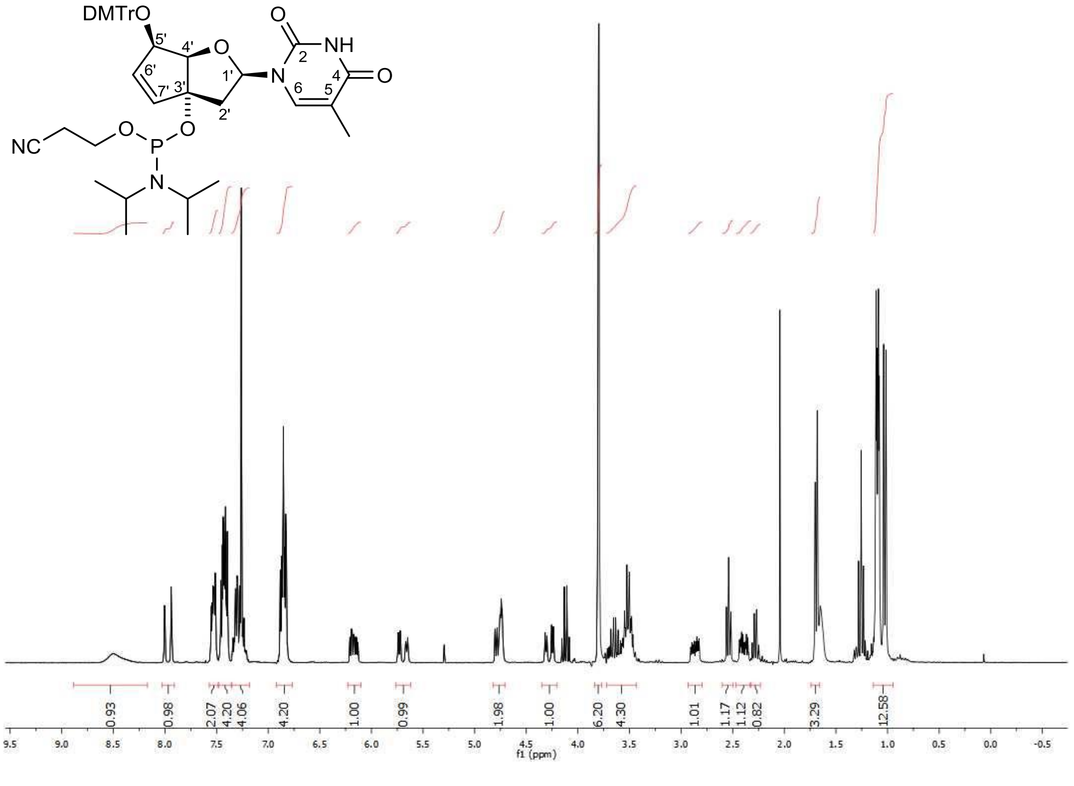

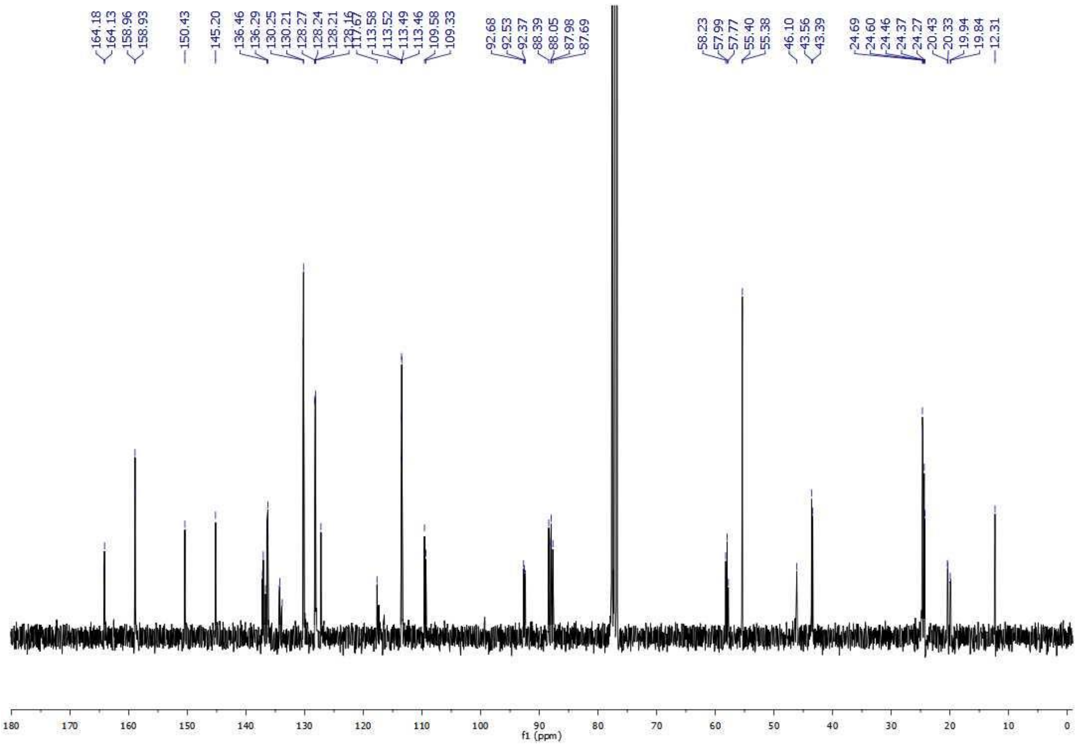

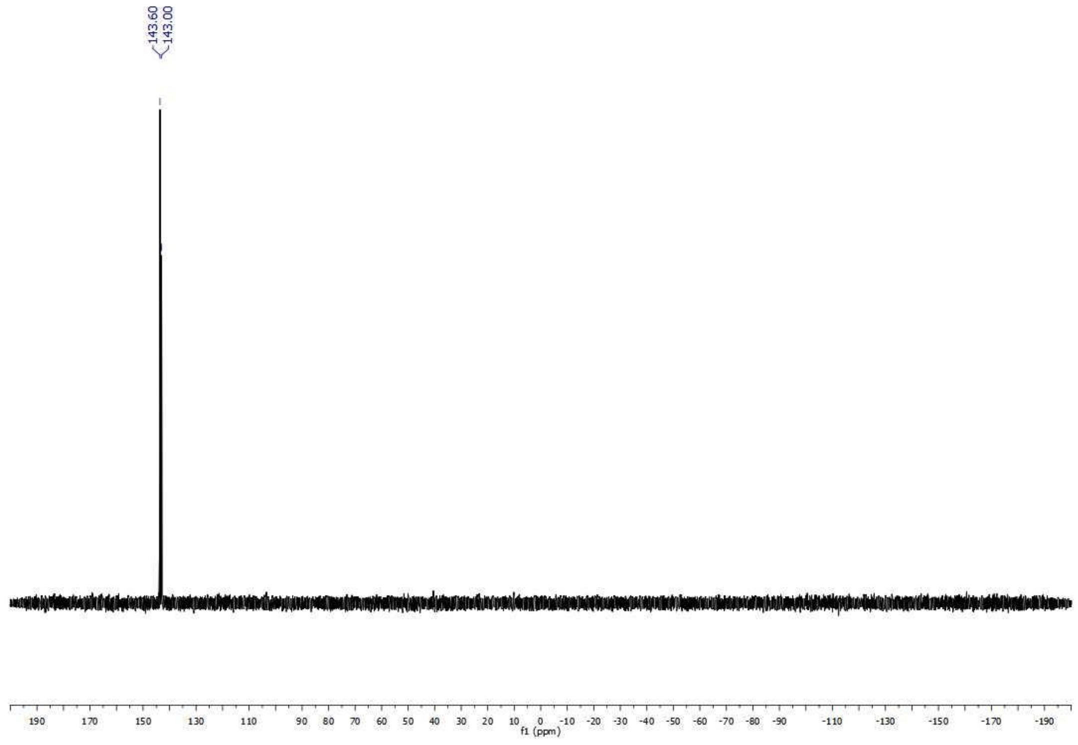

Figure S16: ^{1}H ($CDCl_3$, 400 MHz), ^{13}C ($CDCl_3$, 100 MHz) and ^{31}P NMR ($CDCl_3$, 122 MHz) spectrum of **13**

Crystal-Structure Determination. – A colorless crystals of $C_{13}H_{16}N_2O_5$ (**8**), $C_{12}H_{14}N_2O$ (**11β**) and $C_{13}H_{16}N_2O_5$ (**tc-T**) were mounted in air and used for X-ray structure determination at 173K. All measurements were made on an *Oxford Diffraction SuperNova* area-detector diffractometer using mirror optics monochromated Mo *K*α radiation (λ = 0.71073 Å) and Al filtered. The unit cell constants and an orientation matrix for data collection were obtained from a least-squares refinement of the setting angles of reflections in the ranges 2° < θ < 27.2° (**8**), 2° < θ < 30° (**11β**) and 1.9° < θ < 25.0° (**tc-T**). A total of 1088, frames were collected using ω scans, with 5+5 seconds exposure time, a rotation angle of 1.0° per frame for compound **8**, 1035 frames were collected using ω scans, with 10+10 seconds exposure time for compound **11β** and 1208 frames were collected using ω scans, with 30+30 seconds exposure time for compound **tc-T**, a rotation angle of 0.5° per frame, a crystal-detector distance of 65.0 mm, at T = 173(2) K.

Data reduction was performed using the *CrysAlisPro* program. The intensities were corrected for Lorentz and polarization effects, and an absorption correction based on the multi-scan method using SCALE3 ABSPACK in *CrysAlisPro* was applied. Data collection and refinement parameters are given bellow.

The structures were solved by direct methods using *SHELXS-97*, which revealed the positions of all non-hydrogen atoms of title compounds. The non-hydrogen atoms were refined anisotropically. All H-atoms were placed in geometrically calculated positions and refined using a riding model where each H-atom was assigned a fixed isotropic displacement parameter with a value equal to 1.2Ueq of its parent atom (1.5Ueq for the methyl groups).

Refinement of the structure was carried out on F^2 using full-matrix least-squares procedures, which minimized the function $\Sigma w(F_o^2 - F_c^2)^2$. The weighting scheme was based on counting statistics and included a factor to downweight the intense reflections.

All calculations were performed using the *SHELXL-97* program.

Compound 8: The data did not allow assignment of absolute configuration, which was assigned based on the knowledge of the parent compound. Friedel pairs were then merged before the refinement.

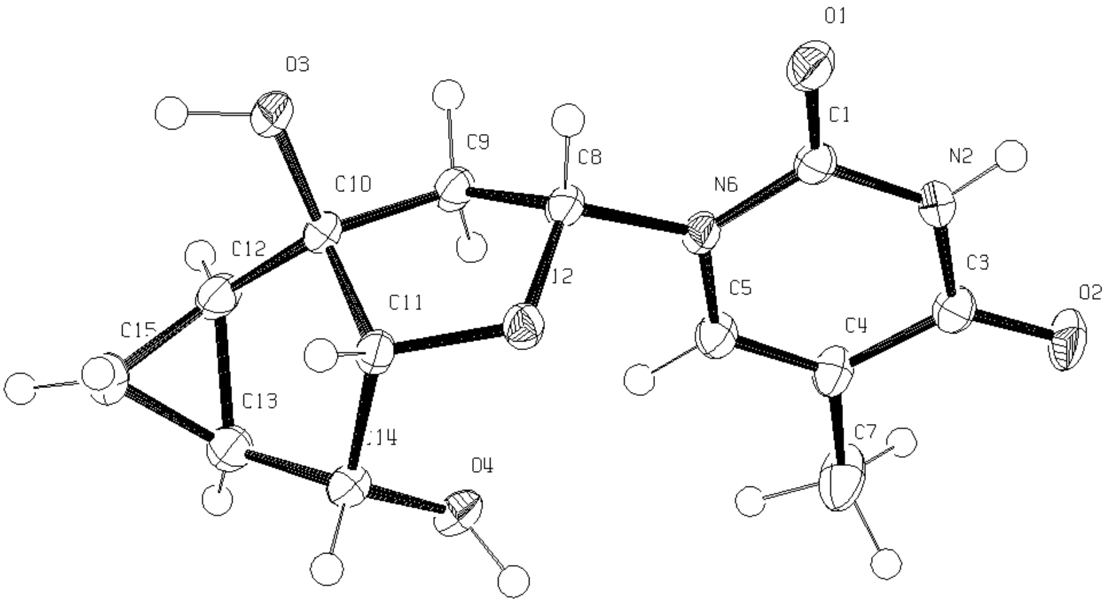

Figure S17: ORTEP view of compound **8** with labeling.

Table S1 - Crystal Data and Details of the Structure Determination for: **8** in P2(1)

Crystal Data

Formula			C13 H16 N2 O5
Formula Weight			280.28
Crystal System			Monoclinic
Space group		$P2_1$	(No. 4)
a, b, c [Angstrom]	9.7669(4)	6.8089(3)	9.9511(4)
alpha, beta, gamma [deg]	90	110.324(5)	90
V [Ang**3]			620.57(5)
Z			2
D(calc) [g/cm**3]			1.500
Mu(MoKa) [/mm]			0.116
F(000)			296
Crystal Size [mm]	0.40 x	0.15 x	0.08

Data Collection	
Temperature (K)	173
Radiation [Angstrom]	MoKa 0.71073
Theta Min-Max [Deg]	2.0, 26.7
Dataset	12:-12 ; 8:-8 ; 12:-12
Tot., Uniq. Data, R(int)	9679, 1394, 0.000
Observed data [I > 2.0 sigma(I)]	1324
**Refinement**	
Nref, Npar	1394, 196
R, wR2, S	3.44, 7.77, 1.06
R, wR2 (I > 2 sigma(I)]	3.17, 7.49
Max. and Av. Shift/Error	0.01, 0.001
Min. and Max. Resd. Dens. [e/Ang^3]	0.19,-0.22

Table S2 - Final Coordinates and Equivalent Isotropic Displacement Parameters of the non-Hydrogen atoms for: **8** in P2(1)

Atom	x	y	z	U(eq) [Ang^2]
O1	0.02923(18)	1.0259(3)	0.10145(17)	0.0251(5)
O2	-0.38533(17)	0.7029(3)	-0.14771(17)	0.0298(5)
O3	0.38044(17)	0.7586(3)	0.57982(17)	0.0254(5)
O4	0.10124(16)	0.2408(3)	0.35014(17)	0.0240(5)
O12	0.21261(16)	0.5775(3)	0.27657(15)	0.0194(5)
N2	-0.1807(2)	0.8644(3)	-0.01342(19)	0.0201(5)
N6	-0.01613(18)	0.7348(3)	0.19546(18)	0.0179(5)
C1	-0.0494(2)	0.8834(4)	0.0950(2)	0.0181(6)
C3	-0.2770(2)	0.7070(4)	-0.0382(2)	0.0205(6)
C4	-0.2377(2)	0.5585(4)	0.0727(2)	0.0226(7)
C5	-0.1095(2)	0.5764(4)	0.1803(2)	0.0207(6)
C7	-0.3343(3)	0.3827(4)	0.0580(3)	0.0394(9)
C8	0.1281(2)	0.7322(4)	0.3085(2)	0.0181(6)
C9	0.1281(2)	0.6888(4)	0.4587(2)	0.0200(6)
C10	0.2795(2)	0.5992(4)	0.5326(2)	0.0182(6)
C11	0.3017(2)	0.4838(4)	0.4074(2)	0.0180(6)
C12	0.2906(3)	0.4483(4)	0.6458(2)	0.0237(7)
C13	0.2822(3)	0.2468(4)	0.5795(3)	0.0251(7)
C14	0.2546(2)	0.2693(4)	0.4217(2)	0.0210(7)
C15	0.4236(3)	0.3191(4)	0.6830(3)	0.0315(8)

U(eq) = 1/3 of the trace of the orthogonalized U Tensor

Table S3 - Hydrogen Atom Positions and Isotropic Displacement Parameters for: **8** in P2(1)

Atom	x	y	z	U(iso) [Ang^2]
H2	-0.20727	0.96269	-0.07442	0.0241
H3	0.455(2)	0.724(5)	0.6675(15)	0.0300

H4	0.089(3)	0.183(4)	0.2600(15)	0.0300
H5	-0.08098	0.47489	0.25005	0.0249
H7A	-0.304(2)	0.296(3)	0.1547(14)	0.0300
H7B	-0.4445(12)	0.436(4)	0.0343(17)	0.0300
H7C	-0.331(2)	0.291(3)	-0.0293(13)	0.0300
H8	0.17767	0.86086	0.30919	0.0216
H9A	0.04990	0.59471	0.45606	0.0240
H9B	0.11542	0.81049	0.50759	0.0240
H11	0.40675	0.48769	0.41585	0.0216
H12	0.24294	0.47270	0.71840	0.0284
H13	0.22963	0.14033	0.61112	0.0301
H14	0.31305	0.17295	0.38809	0.0252
H15A	0.50087	0.35617	0.64450	0.0378
H15B	0.45956	0.25904	0.77956	0.0378

===

The Temperature Factor has the Form of Exp(-T) Where
T = 8*(Pi**2)*U*(Sin(Theta)/Lambda)**2 for Isotropic Atoms

Table S4 - (An)isotropic Displacement Parameters for: **8** in P2(1)

Atom	U(1,1) or U	U(2,2)	U(3,3)	U(2,3)	U(1,3)	U(1,2)
O1	0.0298(8)	0.0241(10)	0.0185(8)	0.0001(7)	0.0046(7)	-0.0083(8)
O2	0.0200(8)	0.0399(12)	0.0206(8)	0.0035(8)	-0.0042(6)	-0.0028(8)
O3	0.0239(8)	0.0203(10)	0.0227(8)	-0.0011(8)	-0.0037(7)	-0.0046(8)
O4	0.0224(8)	0.0252(10)	0.0218(8)	-0.0068(8)	0.0044(7)	-0.0058(8)
O12	0.0207(8)	0.0224(9)	0.0146(7)	-0.0005(7)	0.0056(6)	0.0047(7)
N2	0.0208(9)	0.0203(10)	0.0164(9)	0.0031(8)	0.0029(8)	0.0024(8)
N6	0.0158(8)	0.0188(10)	0.0157(9)	-0.0004(9)	0.0013(7)	-0.0023(9)
C1	0.0216(10)	0.0186(12)	0.0150(10)	-0.0014(10)	0.0074(8)	0.0000(10)
C3	0.0164(10)	0.0248(13)	0.0196(10)	-0.0011(10)	0.0055(8)	-0.0008(10)
C4	0.0192(10)	0.0280(14)	0.0184(11)	0.0006(10)	0.0037(9)	-0.0038(10)
C5	0.0223(11)	0.0217(12)	0.0178(10)	0.0017(10)	0.0066(9)	-0.0034(10)
C7	0.0358(14)	0.0434(19)	0.0283(13)	0.0076(14)	-0.0023(11)	-0.0208(15)
C8	0.0151(9)	0.0193(12)	0.0168(10)	-0.0027(10)	0.0018(8)	-0.0001(10)
C9	0.0193(10)	0.0237(13)	0.0158(10)	-0.0036(10)	0.0045(8)	0.0011(10)
C10	0.0152(10)	0.0182(12)	0.0176(11)	-0.0022(9)	0.0011(8)	-0.0024(9)
C11	0.0170(10)	0.0196(12)	0.0153(10)	-0.0001(9)	0.0029(8)	-0.0006(9)
C12	0.0290(12)	0.0233(14)	0.0163(11)	-0.0023(10)	0.0048(9)	-0.0042(10)
C13	0.0300(12)	0.0204(12)	0.0217(11)	0.0016(11)	0.0051(10)	-0.0024(12)
C14	0.0204(11)	0.0204(13)	0.0196(11)	-0.0033(10)	0.0037(9)	-0.0009(10)
C15	0.0351(14)	0.0251(15)	0.0233(12)	0.0025(11)	-0.0038(11)	0.0013(12)

===

The Temperature Factor has the Form of Exp(-T) Where
T = 8*(Pi**2)*U*(Sin(Theta)/Lambda)**2 for Isotropic Atoms
T = 2*(Pi**2)*Sumij(h(i)*h(j)*U(i,j)*Astar(i)*Astar(j)), for
Anisotropic Atoms. Astar(i) are Reciprocal Axial Lengths and
h(i) are the Reflection Indices.

Table S5 - Bond Distances (Angstrom) for: **8** in P2(1)

O1	-C1	1.225(3)	C10	-C12	1.501(3)
O2	-C3	1.228(3)	C11	-C14	1.553(4)
O3	-C10	1.431(3)	C12	-C13	1.512(4)
O4	-C14	1.431(3)	C12	-C15	1.505(4)
O12	-C8	1.440(3)	C13	-C14	1.506(3)
O12	-C11	1.440(3)	C13	-C15	1.491(4)
O3	-H3	0.952(17)	C5	-H5	0.9500
O4	-H4	0.948(18)	C7	-H7A	1.079(16)
N2	-C3	1.390(3)	C7	-H7B	1.081(16)
N2	-C1	1.365(3)	C7	-H7C	1.079(16)
N6	-C8	1.466(3)	C8	-H8	1.0000
N6	-C5	1.386(3)	C9	-H9A	0.9900
N6	-C1	1.380(3)	C9	-H9B	0.9900
N2	-H2	0.8800	C11	-H11	1.0000
C3	-C4	1.447(3)	C12	-H12	1.0000
C4	-C5	1.341(3)	C13	-H13	1.0000
C4	-C7	1.500(4)	C14	-H14	1.0000
C8	-C9	1.524(3)	C15	-H15A	0.9900
C9	-C10	1.531(3)	C15	-H15B	0.9900
C10	-C11	1.551(3)			

Table S6 - Bond Angles (Degrees)for: **8** in P2(1)

C8	-O12	-C11	109.72(15)	C11	-C10	-C12	105.2(2)
C10	-O3	-H3	109.5(19)	C10	-C11	-C14	105.93(17)
C14	-O4	-H4	107.1(18)	O12	-C11	-C10	107.03(19)
C1	-N2	-C3	127.2(2)	O12	-C11	-C14	113.24(17)
C1	-N6	-C8	119.12(19)	C10	-C12	-C15	114.6(2)
C1	-N6	-C5	120.75(18)	C13	-C12	-C15	59.22(19)
C5	-N6	-C8	119.64(19)	C10	-C12	-C13	108.36(19)
C1	-N2	-H2	116.00	C12	-C13	-C14	109.0(2)
C3	-N2	-H2	116.00	C12	-C13	-C15	60.14(18)
O1	-C1	-N2	121.1(2)	C14	-C13	-C15	118.3(2)
O1	-C1	-N6	123.87(19)	O4	-C14	-C13	106.41(19)
N2	-C1	-N6	115.0(2)	C11	-C14	-C13	103.88(19)
N2	-C3	-C4	114.81(18)	O4	-C14	-C11	111.98(19)
O2	-C3	-C4	125.4(2)	C12	-C15	-C13	60.64(19)
O2	-C3	-N2	119.8(2)	N6	-C5	-H5	118.00
C3	-C4	-C7	119.49(19)	C4	-C5	-H5	118.00
C5	-C4	-C7	122.1(2)	C4	-C7	-H7A	112.4(10)
C3	-C4	-C5	118.2(2)	C4	-C7	-H7B	107.4(14)
N6	-C5	-C4	123.7(2)	C4	-C7	-H7C	110.3(11)
O12	-C8	-N6	108.13(17)	H7A	-C7	-H7B	108.8(15)
O12	-C8	-C9	105.66(19)	H7A	-C7	-H7C	109.0(14)
N6	-C8	-C9	115.32(17)	H7B	-C7	-H7C	108.9(14)

C8	-C9	-C10	102.67(16)	O12	-C8	-H8	109.00
O3	-C10	-C11	112.05(17)	N6	-C8	-H8	109.00
O3	-C10	-C12	114.26(17)	C9	-C8	-H8	109.00
C9	-C10	-C11	101.38(15)	C8	-C9	-H9A	111.00
O3	-C10	-C9	107.2(2)	C8	-C9	-H9B	111.00
C9	-C10	-C12	116.1(2)	C10	-C9	-H9A	111.00
C10	-C9	-H9B	111.00	C15	-C13	-H13	118.00
H9A	-C9	-H9B	109.00	O4	-C14	-H14	111.00
O12	-C11	-H11	110.00	C11	-C14	-H14	111.00
C10	-C11	-H11	110.00	C13	-C14	-H14	111.00
C14	-C11	-H11	110.00	C12	-C15	-H15A	118.00
C10	-C12	-H12	120.00	C12	-C15	-H15B	118.00
C13	-C12	-H12	120.00	C13	-C15	-H15A	118.00
C15	-C12	-H12	120.00	C13	-C15	-H15B	118.00
C12	-C13	-H13	118.00	H15A	-C15	-H15B	115.00
C14	-C13	-H13	118.00				

Table S7 - Torsion Angles (Degrees)for: **8** in P2(1)

C8	-O12	-C11	-C14	112.0(2)
C11	-O12	-C8	-N6	-142.76(18)
C11	-O12	-C8	-C9	-18.8(2)
C8	-O12	-C11	-C10	-4.4(2)
C3	-N2	-C1	-O1	176.4(2)
C1	-N2	-C3	-C4	6.6(3)
C1	-N2	-C3	-O2	-173.5(2)
C3	-N2	-C1	-N6	-5.1(3)
C8	-N6	-C1	-N2	174.35(19)
C8	-N6	-C1	-O1	-7.3(3)
C5	-N6	-C1	-N2	2.5(3)
C1	-N6	-C8	-O12	-106.4(2)
C5	-N6	-C8	-O12	65.6(2)
C5	-N6	-C1	-O1	-179.2(2)
C1	-N6	-C5	-C4	-2.1(3)
C8	-N6	-C5	-C4	-174.0(2)
C1	-N6	-C8	-C9	135.7(2)
C5	-N6	-C8	-C9	-52.4(3)
N2	-C3	-C4	-C5	-5.5(3)
O2	-C3	-C4	-C7	-0.4(4)
N2	-C3	-C4	-C7	179.5(2)
O2	-C3	-C4	-C5	174.7(2)
C7	-C4	-C5	-N6	178.6(2)
C3	-C4	-C5	-N6	3.7(3)
N6	-C8	-C9	-C10	153.6(2)
O12	-C8	-C9	-C10	34.2(2)
C8	-C9	-C10	-C12	-148.6(2)
C8	-C9	-C10	-C11	-35.3(2)

C8	-C9	-C10	-O3	82.3(2)
O3	-C10	-C11	-C14	150.07(18)
O3	-C10	-C11	-O12	-88.8(2)
C12	-C10	-C11	-O12	146.45(19)
C9	-C10	-C11	-O12	25.2(2)
C9	-C10	-C11	-C14	-95.9(2)
O3	-C10	-C12	-C15	-72.8(3)
C9	-C10	-C12	-C13	97.9(3)
C9	-C10	-C12	-C15	161.7(2)
C11	-C10	-C12	-C13	-13.3(3)
C11	-C10	-C12	-C15	50.6(2)
C12	-C10	-C11	-C14	25.4(2)
O3	-C10	-C12	-C13	-136.6(2)
O12	-C11	-C14	-O4	-30.1(2)
O12	-C11	-C14	-C13	-144.54(19)
C10	-C11	-C14	-O4	86.88(19)
C10	-C11	-C14	-C13	-27.5(2)
C10	-C12	-C13	-C14	-4.2(3)
C10	-C12	-C13	-C15	108.2(3)
C15	-C12	-C13	-C14	-112.4(3)
C10	-C12	-C15	-C13	-97.5(2)
C12	-C13	-C14	-O4	-98.7(3)
C12	-C13	-C14	-C11	19.7(3)
C15	-C13	-C14	-O4	-164.3(2)
C15	-C13	-C14	-C11	-46.0(3)
C14	-C13	-C15	-C12	96.7(3)

Table S8 - Contact Distances(Angstrom) for: **8** in P2(1)

O1	.O4_a	2.748(2)	O3	.H11_f	2.5900
O1	.C5_b	3.181(3)	O4	.H9A	2.7500
O1	.C1_b	3.171(3)	O4	.H8_g	2.7600
O1	.N2_b	3.028(3)	O4	.H5	2.3400
O1	.N6_b	3.241(2)	O12	.H5	2.8700
O1	.C3_b	2.972(3)	O12	.H2_h	2.1400
O1	.C4_b	3.106(3)	N2	.O1_h	3.028(3)
O2	.O3_c	2.902(2)	N2	.O12_b	2.914(2)
O2	.C14_b	3.419(3)	N6	.O1_h	3.241(2)
O2	.C15_c	3.312(3)	C1	.O1_h	3.171(3)
O2	.C7_d	3.390(4)	C3	.O1_h	2.972(3)
O3	.O2_e	2.902(2)	C4	.O1_h	3.106(3)
O3	.O12	3.143(2)	C5	.C13_i	3.564(4)
O4	.C9	3.216(3)	C5	.O4	3.146(3)
O4	.O12	2.744(3)	C5	.O1_h	3.181(3)
O4	.O1_g	2.748(2)	C7	.O2_j	3.390(4)
O4	.C5	3.146(3)	C8	.O4	3.393(3)
O4	.C8	3.393(3)	C9	.O4	3.216(3)
O12	.N2_h	2.914(2)	C13	.C5_k	3.564(4)

O12	.O4	2.744(3)	C14	.O2_h	3.419(3)
O12	.O3	3.143(2)	C15	.O2_e	3.312(3)
O1	.H8	2.3600	C1	.H4_a	2.67(2)
O1	.H4_a	1.83(2)	C5	.H13_i	2.7500
O2	.H14_b	2.7300	C5	.H9A	2.6500
O2	.H7B	2.77(2)	C7	.H15B_c	2.9300
O2	.H3_c	1.960(16)	C9	.H5	2.7700
O2	.H7B_d	2.803(19)	C11	.H15A	2.6300
O3	.H8	2.8200	C12	.H7A_i	3.063(18)
C14	.H9A	3.0800	H7C	.H15B_c	2.2700
C14	.H8_g	2.9900	H8	.O1	2.3600
C15	.H3	2.78(3)	H8	.O3	2.8200
C15	.H7C_e	3.035(15)	H8	.O4_a	2.7600
C15	.H11	2.8500	H8	.C14_a	2.9900
H2	.O12_b	2.1400	H8	.H4_a	2.3500
H3	.O2_e	1.960(16)	H8	.H14_a	2.4800
H3	.C15	2.78(3)	H9A	.O4	2.7500
H3	.H15A	2.5700	H9A	.C5	2.6500
H3	.H11_f	2.5500	H9A	.C14	3.0800
H3	.H14_f	2.5400	H9A	.H5	2.1600
H4	.O1_g	1.83(2)	H9A	.H13_i	2.5900
H4	.C1_g	2.67(2)	H9B	.H13_a	2.5600
H4	.H5	2.5700	H11	.C15	2.8500
H4	.H8_g	2.3500	H11	.H15A	2.3200
H5	.O4	2.3400	H11	.O3_l	2.5900
H5	.O12	2.8700	H11	.H3_l	2.5500
H5	.C9	2.7700	H12	.H7A_i	2.5000
H5	.H4	2.5700	H13	.H9B_g	2.5600
H5	.H7A	2.3900	H13	.C5_k	2.7500
H5	.H9A	2.1600	H13	.H5_k	2.5800
H5	.H13_i	2.5800	H13	.H9A_k	2.5900
H7A	.H5	2.3900	H14	.H8_g	2.4800
H7A	.C12_k	3.063(18)	H14	.O2_h	2.7300
H7A	.H12_k	2.5000	H14	.H3_l	2.5400
H7B	.O2	2.77(2)	H15A	.C11	2.6300
H7B	.O2_j	2.803(19)	H15A	.H3	2.5700
H7C	.C15_c	3.035(15)	H15A	.H11	2.3200
H15B	.C7_e	2.9300	H15B	.H7C_e	2.2700

Table S9 - Hydrogen Bonds (Angstrom, Deg)for: **8** in P2(1)

N2	--	H2	.. O12	0.8800	2.1400	2.914(2)	146.00	2_555
O3	--	H3	.. O2	0.952(17)	1.960(16)	2.902(2)	170(3)	1_656
O4	--	H4	.. O1	0.948(18)	1.83(2)	2.748(2)	163(3)	1_545
C5	--	H5	.. O4	0.9500	2.3400	3.146(3)	143.00	.
C8	--	H8	.. O1	1.0000	2.3600	2.792(3)	105.00	.
C11	--	H11	.. O3	1.0000	2.5900	3.425(3)	141.00	2_646

```
 Translation of Symmetry Code to Equiv.Pos

a =[  1565.00 ]  = x,1+y,z
b =[  2555.00 ]  = -x,1/2+y,-z
c =[  1454.00 ]  = -1+x,y,-1+z
d =[  2455.00 ]  = -1-x,1/2+y,-z
e =[  1656.00 ]  = 1+x,y,1+z
f =[  2656.00 ]  = 1-x,1/2+y,1-z
g =[  1545.00 ]  = x,-1+y,z
h =[  2545.00 ]  = -x,-1/2+y,-z
i =[  2556.00 ]  = -x,1/2+y,1-z
j =[  2445.00 ]  = -1-x,-1/2+y,-z
k =[  2546.00 ]  = -x,-1/2+y,1-z
l =[  2646.00 ]  = 1-x,-1/2+y,1-z
```

Compound 11β: The crystal space group type is chiral, only one molecule crystallize in the unit cell. Absolute configuration cannot be unambiguously determined from X-ray data (Flack parameter -0.0(9)), therefore it is assigned based on the known stereochemistry of the reactant.

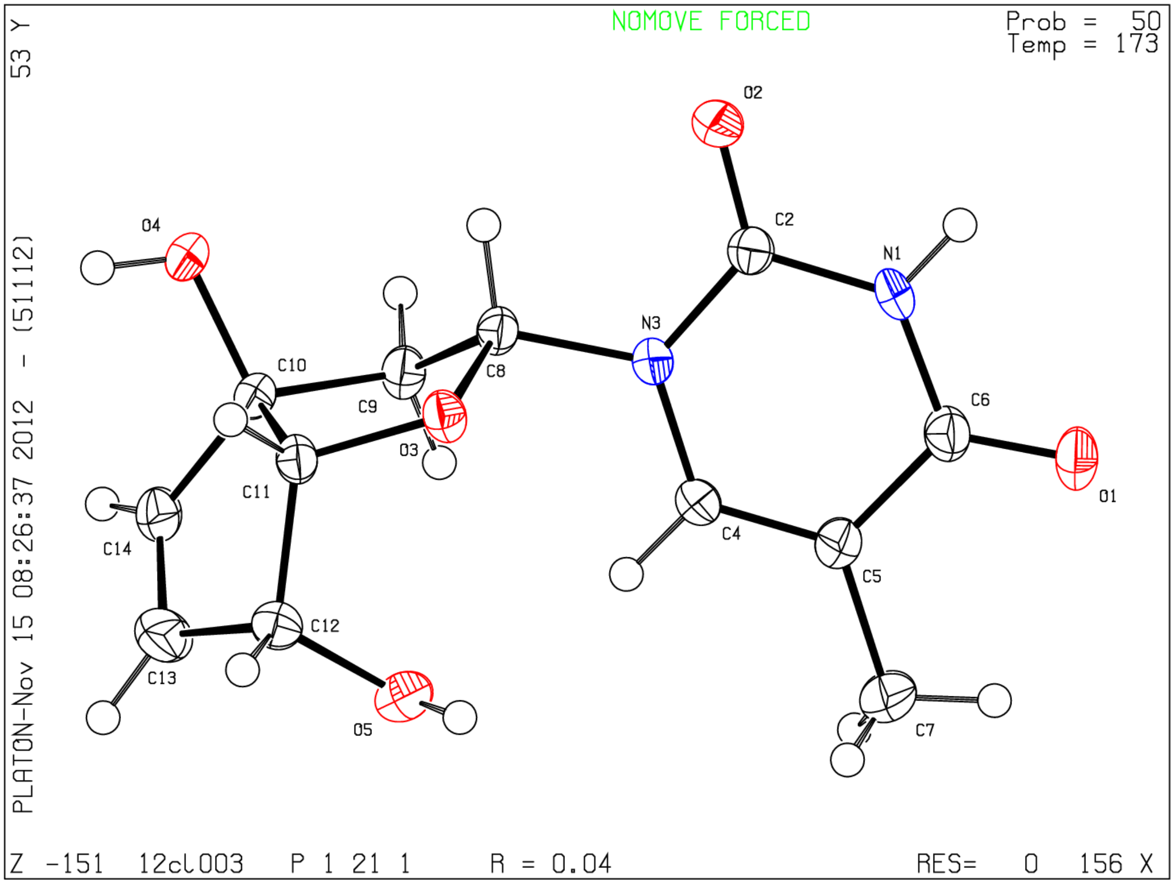

Figure S18: ORTEP view of compound **11β** with labeling.

Table S10 - Crystal Data and Details of the Structure Determination

for: **11β** P 1 21 1 R = 0.04

Crystal Data

Formula			C12 H14 N2 O5
Formula Weight			266.25
Crystal System			Monoclinic
Space group		P21	(No. 4)
a, b, c [Angstrom]	9.6135(2)	6.7389(1)	10.0548(2)
alpha, beta, gamma [deg]	90	111.785(3)	90
V [Ang**3]			604.87(2)
Z			2

D(calc) [g/cm**3]	1.462
Mu(MoKa) [/mm]	0.115
F(000)	280
Crystal Size [mm]	0.09 x 0.14 x 0.68
Data Collection	
Temperature (K)	173
Radiation [Angstrom]	MoKa 0.71069
Theta Min-Max [Deg]	2.2, 26.7
Dataset	-11: 12 ; -8: 8 ; -12: 11
Tot., Uniq. Data, R(int)	8853, 2358, 0.023
Observed data [I > 0.0 sigma(I)]	2256
Refinement	
Nref, Npar	2358, 174
R, wR2, S	0.0351, 0.0934, 1.05
w = 1/[\s^2^(Fo^2^)+(0.0483P)^2^+0.2494P] where P=(Fo^2^+2Fc^2^)/3	
Max. and Av. Shift/Error	0.00, 0.00
Flack x	-0.1(10)
Min. and Max. Resd. Dens. [e/Ang^3]	-0.27, 0.32

Table S11 - Final Coordinates and Equivalent Isotropic Displacement Parameters of the non-Hydrogen atoms for: **11β** P 1 21 1 R = 0.04

Atom	x	y	z	U(eq) [Ang^2]
O1	0.11514(17)	0.8680(3)	0.32482(16)	0.0380(5)
O2	0.51115(16)	1.2267(2)	0.60733(15)	0.0273(4)
O3	0.70955(14)	0.7573(2)	0.78469(13)	0.0206(4)
O4	0.86987(15)	0.9709(2)	1.08671(15)	0.0273(4)
O5	0.61283(15)	0.4162(2)	0.87397(16)	0.0275(4)
N1	0.30957(18)	1.0457(3)	0.47299(17)	0.0217(5)
N3	0.47746(17)	0.9170(2)	0.68563(16)	0.0197(4)
C2	0.4395(2)	1.0735(3)	0.5911(2)	0.0200(5)
C4	0.3878(2)	0.7510(3)	0.6632(2)	0.0215(5)
C5	0.2609(2)	0.7262(3)	0.5488(2)	0.0243(6)
C6	0.2199(2)	0.8793(3)	0.4396(2)	0.0243(6)
C7	0.1645(3)	0.5443(4)	0.5230(3)	0.0403(7)
C8	0.6226(2)	0.9199(3)	0.80435(19)	0.0194(5)
C9	0.6163(2)	0.8905(3)	0.95188(19)	0.0212(5)
C10	0.7715(2)	0.8058(3)	1.03685(19)	0.0198(5)
C11	0.8033(2)	0.6813(3)	0.92307(19)	0.0197(5)
C12	0.7673(2)	0.4646(3)	0.9482(2)	0.0233(5)
C13	0.7917(3)	0.4693(4)	1.1055(2)	0.0336(7)
C14	0.7835(2)	0.6533(3)	1.1513(2)	0.0253(6)

U(eq) = 1/3 of the trace of the orthogonalized U Tensor

Table S12 - Hydrogen Atom Positions and Isotropic Displacement

Parameters for: **11β** P 1 21 1 R = 0.04

Atom	x	y	z	U(iso) [Ang^2]
H1	0.28080	1.14530	0.41230	0.0260
H4	0.41760	0.64830	0.73280	0.0260
H4A	0.94880	0.93390	1.15280	0.0410
H5	0.59980	0.37680	0.79090	0.0410
H7A	0.07790	0.55940	0.43330	0.0600
H7B	0.12980	0.52670	0.60250	0.0600
H7C	0.22280	0.42790	0.51660	0.0600
H8	0.67500	1.04750	0.80260	0.0230
H9A	0.53630	0.79610	0.94870	0.0250
H9B	0.60000	1.01790	0.99290	0.0250
H11	0.91120	0.69350	0.93590	0.0240
H12	0.83550	0.36820	0.92710	0.0280
H13	0.81050	0.35480	1.16470	0.0400
H14	0.78500	0.68460	1.24400	0.0300

===

The Temperature Factor has the Form of Exp(-T) Where

T = 8*(Pi**2)*U*(Sin(Theta)/Lambda)**2 for Isotropic Atoms

Table S13 - (An)isotropic Displacement Parameters for: **11β** P 1 21 1 R = 0.04

Atom	U(1,1) or U	U(2,2)	U(3,3)	U(2,3)	U(1,3)	U(1,2)
O1	0.0292(8)	0.0422(10)	0.0275(8)	0.0108(7)	-0.0070(7)	-0.0074(7)
O2	0.0317(8)	0.0224(8)	0.0233(7)	0.0043(6)	0.0050(6)	-0.0050(6)
O3	0.0221(7)	0.0217(7)	0.0163(6)	0.0002(6)	0.0051(5)	0.0044(6)
O4	0.0243(7)	0.0210(7)	0.0255(7)	-0.0016(6)	-0.0036(6)	-0.0039(6)
O5	0.0267(7)	0.0244(8)	0.0308(7)	-0.0036(7)	0.0100(6)	-0.0047(6)
N1	0.0218(8)	0.0221(8)	0.0170(8)	0.0066(7)	0.0023(6)	0.0038(7)
N3	0.0198(8)	0.0191(8)	0.0166(7)	0.0015(6)	0.0026(6)	0.0002(7)
C2	0.0212(9)	0.0202(9)	0.0180(9)	-0.0002(7)	0.0066(8)	-0.0003(8)
C4	0.0238(9)	0.0200(10)	0.0184(9)	0.0037(8)	0.0052(7)	-0.0011(8)
C5	0.0201(9)	0.0251(11)	0.0240(9)	0.0020(8)	0.0040(8)	-0.0025(8)
C6	0.0190(9)	0.0279(11)	0.0233(10)	0.0035(8)	0.0047(8)	0.0010(8)
C7	0.0347(12)	0.0344(13)	0.0397(13)	0.0089(11)	-0.0001(10)	-0.0157(11)
C8	0.0167(9)	0.0195(9)	0.0187(9)	-0.0012(8)	0.0029(7)	0.0015(7)
C9	0.0196(9)	0.0230(10)	0.0184(9)	-0.0024(8)	0.0040(7)	0.0031(8)
C10	0.0184(9)	0.0189(10)	0.0183(9)	-0.0018(7)	0.0023(8)	-0.0013(7)
C11	0.0176(9)	0.0209(10)	0.0178(9)	0.0011(7)	0.0034(7)	0.0018(7)
C12	0.0247(9)	0.0189(9)	0.0247(10)	-0.0003(8)	0.0072(8)	0.0016(8)
C13	0.0465(13)	0.0261(11)	0.0235(10)	0.0040(9)	0.0074(9)	-0.0026(10)
C14	0.0273(11)	0.0283(11)	0.0164(10)	0.0022(8)	0.0036(8)	-0.0027(9)

===

The Temperature Factor has the Form of Exp(-T) Where

T = 8*(Pi**2)*U*(Sin(Theta)/Lambda)**2 for Isotropic Atoms

T = 2*(Pi**2)*Sumij(h(i)*h(j)*U(i,j)*Astar(i)*Astar(j)), for Anisotropic Atoms. Astar(i) are Reciprocal Axial Lengths and h(i) are the Reflection Indices.

Table S14 - Bond Distances (Angstrom) for: **11β** P 1 21 1 R = 0.04

O1	-C6	1.220(2)	C9	-C10	1.528(3)
O2	-C2	1.218(2)	C10	-C14	1.515(3)
O3	-C8	1.436(2)	C10	-C11	1.538(3)
O3	-C11	1.442(2)	C11	-C12	1.543(3)
O4	-C10	1.425(2)	C12	-C13	1.511(3)
O5	-C12	1.430(3)	C13	-C14	1.335(3)
O4	-H4A	0.8400	C4	-H4	0.9500
O5	-H5	0.8400	C7	-H7A	0.9800
N1	-C6	1.378(3)	C7	-H7B	0.9800
N1	-C2	1.380(3)	C7	-H7C	0.9800
N3	-C4	1.379(3)	C8	-H8	1.0000
N3	-C8	1.462(2)	C9	-H9A	0.9900
N3	-C2	1.375(2)	C9	-H9B	0.9900
N1	-H1	0.8800	C11	-H11	1.0000
C4	-C5	1.341(3)	C12	-H12	1.0000
C5	-C6	1.451(3)	C13	-H13	0.9500
C5	-C7	1.500(3)	C14	-H14	0.9500
C8	-C9	1.520(3)			

Table S15 - Bond Angles (Degrees) for: **11β** P 1 21 1 R = 0.04

C8	-O3	-C11	109.00(13)	O4	-C10	-C14	114.13(15)
C10	-O4	-H4A	109.00	O3	-C11	-C10	107.36(15)
C12	-O5	-H5	109.00	O3	-C11	-C12	113.34(15)
C2	-N1	-C6	127.21(18)	C10	-C11	-C12	106.01(15)
C4	-N3	-C8	119.59(15)	O5	-C12	-C11	112.51(16)
C2	-N3	-C8	118.83(16)	C11	-C12	-C13	101.21(17)
C2	-N3	-C4	121.31(16)	O5	-C12	-C13	106.19(18)
C2	-N1	-H1	116.00	C12	-C13	-C14	112.0(2)
C6	-N1	-H1	116.00	C10	-C14	-C13	111.44(17)
N1	-C2	-N3	114.45(18)	N3	-C4	-H4	118.00
O2	-C2	-N3	124.44(18)	C5	-C4	-H4	118.00
O2	-C2	-N1	121.10(18)	C5	-C7	-H7A	109.00
N3	-C4	-C5	123.78(18)	C5	-C7	-H7B	109.00
C4	-C5	-C6	117.76(18)	C5	-C7	-H7C	109.00
C4	-C5	-C7	123.9(2)	H7A	-C7	-H7B	109.00
C6	-C5	-C7	118.26(19)	H7A	-C7	-H7C	110.00
O1	-C6	-N1	120.22(19)	H7B	-C7	-H7C	109.00
O1	-C6	-C5	124.7(2)	O3	-C8	-H8	109.00
N1	-C6	-C5	115.13(17)	N3	-C8	-H8	109.00

O3	-C8	-C9	105.73(15)	C9	-C8	-H8	109.00
N3	-C8	-C9	115.07(17)	C8	-C9	-H9A	111.00
O3	-C8	-N3	107.79(14)	C8	-C9	-H9B	111.00
C8	-C9	-C10	102.13(16)	C10	-C9	-H9A	111.00
C9	-C10	-C11	102.14(14)	C10	-C9	-H9B	111.00
C11	-C10	-C14	102.41(16)	H9A	-C9	-H9B	109.00
O4	-C10	-C11	113.47(16)	O3	-C11	-H11	110.00
C9	-C10	-C14	117.47(17)	C10	-C11	-H11	110.00
O4	-C10	-C9	106.72(16)	C12	-C11	-H11	110.00
O5	-C12	-H12	112.00	C14	-C13	-H13	124.00
C11	-C12	-H12	112.00	C10	-C14	-H14	124.00
C13	-C12	-H12	112.00	C13	-C14	-H14	124.00
C12	-C13	-H13	124.00				

Table S16 - Torsion Angles (Degrees) for: **11β** P 1 21 1 R = 0.04

C8	-O3	-C11	-C12	116.94(17)
C11	-O3	-C8	-N3	-146.32(15)
C11	-O3	-C8	-C9	-22.8(2)
C8	-O3	-C11	-C10	0.2(2)
C6	-N1	-C2	-O2	178.4(2)
C2	-N1	-C6	-O1	-173.4(2)
C6	-N1	-C2	-N3	-2.2(3)
C2	-N1	-C6	-C5	6.4(3)
C4	-N3	-C2	-N1	-2.0(3)
C8	-N3	-C2	-O2	-8.5(3)
C4	-N3	-C2	-O2	177.40(19)
C2	-N3	-C8	-O3	-115.68(18)
C4	-N3	-C8	-O3	58.6(2)
C2	-N3	-C8	-C9	126.65(19)
C8	-N3	-C4	-C5	-172.58(19)
C2	-N3	-C4	-C5	1.5(3)
C8	-N3	-C2	-N1	172.11(17)
C4	-N3	-C8	-C9	-59.1(2)
N3	-C4	-C5	-C6	2.9(3)
N3	-C4	-C5	-C7	179.0(2)
C4	-C5	-C6	-O1	173.3(2)
C4	-C5	-C6	-N1	-6.4(3)
C7	-C5	-C6	-O1	-2.9(3)
C7	-C5	-C6	-N1	177.4(2)
O3	-C8	-C9	-C10	35.88(19)
N3	-C8	-C9	-C10	154.70(16)
C8	-C9	-C10	-C11	-34.27(19)
C8	-C9	-C10	-O4	85.07(17)
C8	-C9	-C10	-C14	-145.34(17)
O4	-C10	-C11	-C12	146.02(16)
O4	-C10	-C11	-O3	-92.54(18)
C14	-C10	-C11	-O3	143.97(16)

C14	-C10	-C11	-C12	22.53(19)
O4	-C10	-C14	-C13	-133.1(2)
C9	-C10	-C14	-C13	100.9(2)
C11	-C10	-C14	-C13	-10.0(2)
C9	-C10	-C11	-O3	21.9(2)
C9	-C10	-C11	-C12	-99.51(17)
O3	-C11	-C12	-O5	-30.6(2)
O3	-C11	-C12	-C13	-143.53(18)
C10	-C11	-C12	-O5	86.93(18)
C10	-C11	-C12	-C13	-26.0(2)
O5	-C12	-C13	-C14	-96.7(2)
C11	-C12	-C13	-C14	20.9(3)
C12	-C13	-C14	-C10	-7.1(3)

Table S17 - Contact Distances(Angstrom) for: **11β** P 1 21 1 R = 0.04

O1	.O4_a	2.754(2)	O3	.H4	2.7600
O1	.C14_a	3.343(3)	O3	.H5	2.7800
O1	.C12_b	3.397(3)	O4	.H11_g	2.6600
O2	.C6_b	2.978(3)	O4	.H8	2.8200
O2	.C2_b	3.220(2)	O4	.H13_d	2.8200
O2	.N3_b	3.252(2)	O5	.H8_h	2.7100
O2	.O5_d	2.798(2)	O5	.H9A	2.8400
O2	.C5_b	3.134(3)	O5	.H4	2.4500
O2	.N1_b	3.046(2)	O5	.H9A_i	2.7900
O2	.C4_b	3.214(2)	O5	.H9B_i	2.9100
O3	.O5	2.752(2)	N1	.O2_e	3.046(2)
O3	.O4	3.1930(19)	N1	.O3_b	2.903(2)
O3	.N1_e	2.903(2)	N3	.O2_e	3.252(2)
O4	.O1_f	2.754(2)	N1	.H7C_d	2.7900
O4	.O3	3.1930(19)	C2	.O2_e	3.220(2)
O5	.C9_i	3.288(3)	C4	.O5	3.295(2)
O5	.C9	3.288(2)	C4	.O2_e	3.214(2)
O5	.C4	3.295(2)	C5	.O2_e	3.134(3)
O5	.O2_h	2.798(2)	C6	.O2_e	2.978(3)
O5	.O3	2.752(2)	C9	.O5	3.288(2)
O1	.H4A_a	1.9200	C9	.O5_j	3.288(3)
O1	.H7A	2.4400	C12	.O1_e	3.397(3)
O1	.H12_b	2.7400	C14	.O1_f	3.343(3)
O1	.H7B_c	2.9200	C2	.H7C_d	3.0700
O2	.H8	2.3400	C2	.H5_d	2.8800
O2	.H7C_d	2.9100	C4	.H9A	2.7100
O2	.H5_d	2.0000	C4	.H13_j	3.0900
O3	.H1_e	2.1500	C8	.H5_d	3.0900
C9	.H4	2.8400	H7C	.C2_h	3.0700
H1	.H7C_d	2.3400	H7C	.H1_h	2.3400
H1	.O3_b	2.1500	H8	.O2	2.3400
H4	.O3	2.7600	H8	.O4	2.8200

H4	.O5	2.4500	H8	.O5_d	2.7100
H4	.C9	2.8400	H8	.H5_d	2.3200
H4	.H5	2.4500	H9A	.O5	2.8400
H4	.H9A	2.2700	H9A	.C4	2.7100
H4A	.O1_f	1.9200	H9A	.H4	2.2700
H4A	.H11_g	2.5600	H9A	.O5_j	2.7900
H4A	.H12_g	2.5300	H9A	.H9B_i	2.4800
H5	.O2_h	2.0000	H9B	.O5_j	2.9100
H5	.O3	2.7800	H9B	.H9A_j	2.4800
H5	.C2_h	2.8800	H11	.O4_l	2.6600
H5	.C8_h	3.0900	H11	.H4A_l	2.5600
H5	.H4	2.4500	H11	.H12_g	2.5900
H5	.H8_h	2.3200	H12	.O1_e	2.7400
H7A	.O1	2.4400	H12	.H4A_l	2.5300
H7B	.O1_k	2.9200	H12	.H11_l	2.5900
H7C	.O2_h	2.9100	H13	.O4_h	2.8200
H7C	.N1_h	2.7900	H13	.C4_i	3.0900

Table S18 - Hydrogen Bonds (Angstrom, Deg) for: **11β** P 1 21 1 R = 0.04

N1	-- H1	.. O3	0.8800	2.1500	2.903(2)	143.00	2_656
O4	-- H4A	.. O1	0.8400	1.9200	2.754(2)	171.00	1_656
O5	-- H5	.. O2	0.8400	2.0000	2.798(2)	159.00	1_545
C4	-- H4	.. O5	0.9500	2.4500	3.295(2)	148.00	.
C7	-- H7A	.. O1	0.9800	2.4400	2.873(3)	107.00	.
C8	-- H8	.. O2	1.0000	2.3400	2.788(2)	106.00	.

Translation of Symmetry Code to Equiv.Pos

a =[1454.00] = -1+x,y,-1+z
b =[2656.00] = 1-x,1/2+y,1-z
c =[2556.00] = -x,1/2+y,1-z
d =[1565.00] = x,1+y,z
e =[2646.00] = 1-x,-1/2+y,1-z
f =[1656.00] = 1+x,y,1+z
g =[2757.00] = 2-x,1/2+y,2-z
h =[1545.00] = x,-1+y,z
i =[2647.00] = 1-x,-1/2+y,2-z
j =[2657.00] = 1-x,1/2+y,2-z
k =[2546.00] = -x,-1/2+y,1-z
l =[2747.00] = 2-x,-1/2+y,2-z

Compound tc-T: The quality of the data is highly affected by the very small size of the crystals and their poor quality. Even after cooling, diffraction remains quite limited and for this reason there is a very large internal agreement index and the low precision on bond distances. Absolute configuration could not be determined.

There are two independent molecules in the asymmetric unit, which differ for the conformation of the two five member rings. Therefore, the possibility of higher symmetry or smaller unit cell dimensions could be safely excluded.

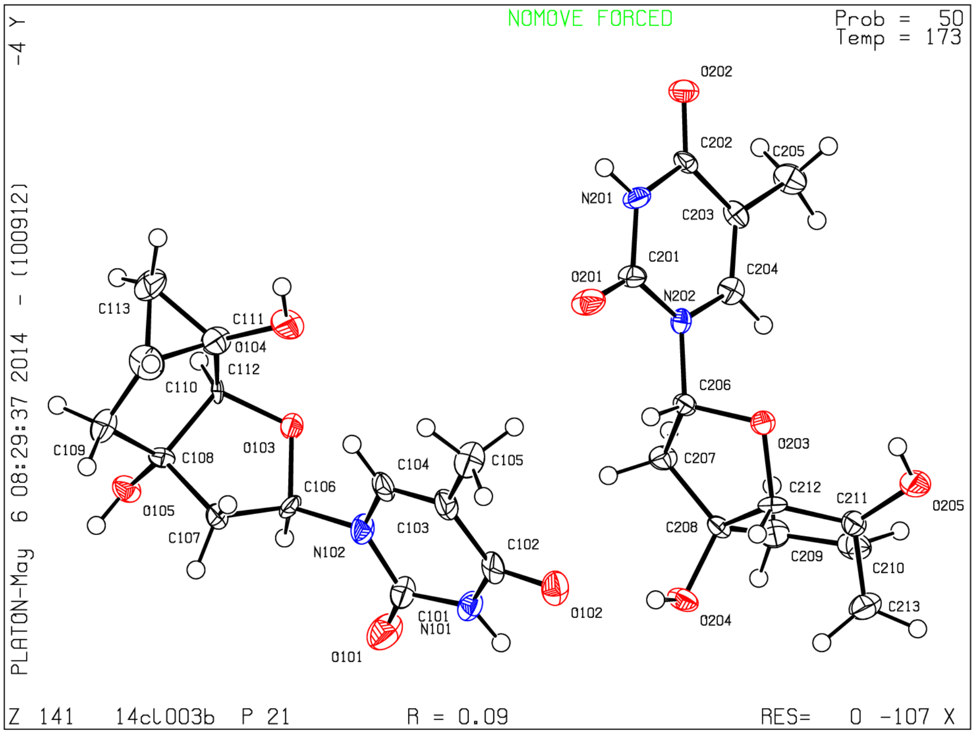

Figure S19: ORTEP view of compound **tc-T** with labeling.

Table S19. Crystal data and structure refinement for **tc-T**.

Identification code	shelx	
Empirical formula	C13 H16 N2 O5	
Formula weight	280.28	
Temperature	173(2) K	
Wavelength	0.71073 Å	
Crystal system	Monoclinic	
Space group	P 21	
Unit cell dimensions	a = 14.1759(10) Å	α= 90°.
	b = 6.0768(4) Å	β= 95.400(6)°.

	c = 14.6965(9) Å	γ = 90°.
Volume	1260.40(14) $Å^3$	
Z	4	
Density (calculated)	1.477 Mg/m^3	
Absorption coefficient	0.115 mm^{-1}	
F(000)	592	
Crystal size	0.1217 x 0.0319 x 0.0281 mm^3	
Theta range for data collection	1.908 to 25.023°.	
Index ranges	-16<=h<=16, -7<=k<=7, -17<=l<=17	
Reflections collected	10070	
Independent reflections	4366 [R(int) = 0.0703]	
Completeness to theta = 25.000°	99.9 %	
Absorption correction	Semi-empirical from equivalents	
Max. and min. transmission	1 and 0.80897	
Refinement method	Full-matrix least-squares on F^2	
Data / restraints / parameters	4366 / 1 / 364	
Goodness-of-fit on F^2	1.085	
Final R indices [I>2sigma(I)]	R1 = 0.0918, wR2 = 0.2292	
R indices (all data)	R1 = 0.1179, wR2 = 0.2493	
Absolute structure parameter	0.0(10)	
Extinction coefficient	0.046(9)	
Largest diff. peak and hole	0.620 and -0.425 e.$Å^{-3}$	

Table S20. Atomic coordinates (x 10^4) and equivalent isotropic displacement parameters ($Å^2$x 10^3) for **tc-T**. U(eq) is defined as one third of the trace of the orthogonalized U^{ij} tensor.

	x	y	z	U(eq)
C(101)	6145(7)	8007(18)	6889(7)	26(2)
C(102)	4939(7)	10441(17)	6111(7)	23(2)
C(103)	4957(7)	11776(18)	6907(7)	25(2)
C(104)	5519(7)	11163(17)	7644(7)	23(2)
C(105)	4337(8)	13789(17)	6893(7)	30(3)
C(106)	6720(7)	8822(19)	8479(6)	22(2)
C(107)	7465(7)	10601(18)	8722(6)	21(2)
C(108)	7667(7)	10350(16)	9746(6)	16(2)
C(109)	7972(8)	12469(18)	10226(7)	29(3)

C(110)	7060(8)	13619(19)	10350(8)	33(3)
C(111)	6270(8)	12015(18)	10229(7)	25(2)
C(113)	6548(8)	12910(20)	11159(7)	35(3)
C(201)	1551(7)	11467(17)	7693(7)	22(2)
C(202)	321(6)	9907(16)	8576(6)	18(2)
C(203)	217(7)	8085(16)	7921(7)	18(2)
C(204)	770(7)	8074(17)	7231(7)	22(2)
C(205)	-495(8)	6307(18)	8074(7)	29(3)
C(206)	1923(6)	9628(16)	6296(6)	16(2)
C(207)	2581(7)	7674(17)	6207(6)	21(2)
C(209)	2049(8)	4804(17)	4994(7)	26(2)
C(210)	1282(8)	5063(18)	4221(7)	30(3)
C(211)	1036(7)	7460(17)	4128(6)	22(2)
C(212)	1704(7)	8722(16)	4771(6)	18(2)
C(213)	1507(9)	6340(20)	3392(7)	33(3)
C(208)	2474(6)	7084(16)	5188(6)	16(2)
C(112)	6677(6)	9766(16)	10033(6)	18(2)
N(101)	5508(6)	8618(15)	6160(5)	24(2)
N(102)	6095(6)	9269(14)	7647(5)	23(2)
N(201)	995(6)	11413(14)	8417(5)	21(2)
N(202)	1415(5)	9672(13)	7120(5)	16(2)
O(101)	6658(6)	6425(14)	6859(5)	40(2)
O(102)	4414(5)	10805(13)	5389(5)	30(2)
O(103)	6183(4)	8715(12)	9252(4)	21(2)
O(104)	5397(5)	12571(13)	9764(5)	33(2)
O(105)	8315(5)	8552(12)	9951(4)	23(2)
O(201)	2109(5)	12976(12)	7590(5)	31(2)
O(202)	-133(5)	10169(11)	9238(5)	25(2)
O(203)	1234(4)	9519(11)	5531(4)	19(2)
O(204)	3321(5)	7165(12)	4759(5)	25(2)
O(205)	107(5)	8210(14)	3936(5)	30(2)

Table S21. Bond lengths [Å] and angles [°] for **tc-T**.

C(101)-O(101)	1.209(13)
C(101)-N(102)	1.360(13)
C(101)-N(101)	1.385(13)

C(102)-O(102)	1.257(12)
C(102)-N(101)	1.367(14)
C(102)-C(103)	1.423(15)
C(103)-C(104)	1.335(14)
C(103)-C(105)	1.506(14)
C(104)-N(102)	1.411(13)
C(106)-O(103)	1.427(11)
C(106)-N(102)	1.466(12)
C(106)-C(107)	1.531(14)
C(107)-C(108)	1.512(12)
C(108)-O(105)	1.440(11)
C(108)-C(109)	1.512(14)
C(108)-C(112)	1.545(13)
C(109)-C(110)	1.497(16)
C(110)-C(111)	1.482(16)
C(110)-C(113)	1.513(16)
C(111)-O(104)	1.397(12)
C(111)-C(113)	1.489(14)
C(111)-C(112)	1.521(14)
C(201)-O(201)	1.230(12)
C(201)-N(202)	1.380(13)
C(201)-N(201)	1.382(12)
C(202)-O(202)	1.227(11)
C(202)-N(201)	1.359(12)
C(202)-C(203)	1.465(14)
C(203)-C(204)	1.338(13)
C(203)-C(205)	1.510(14)
C(204)-N(202)	1.354(12)
C(206)-O(203)	1.419(11)
C(206)-N(202)	1.467(12)
C(206)-C(207)	1.523(13)
C(207)-C(208)	1.533(13)
C(209)-C(210)	1.505(16)
C(209)-C(208)	1.527(14)
C(210)-C(211)	1.501(15)
C(210)-C(213)	1.505(15)
C(211)-O(205)	1.398(12)
C(211)-C(213)	1.486(14)

C(211)-C(212)	1.486(13)
C(212)-O(203)	1.438(10)
C(212)-C(208)	1.560(13)
C(208)-O(204)	1.409(11)
C(112)-O(103)	1.436(11)
O(101)-C(101)-N(102)	123.8(9)
O(101)-C(101)-N(101)	122.4(9)
N(102)-C(101)-N(101)	113.7(9)
O(102)-C(102)-N(101)	119.2(10)
O(102)-C(102)-C(103)	123.8(10)
N(101)-C(102)-C(103)	116.9(9)
C(104)-C(103)-C(102)	118.1(10)
C(104)-C(103)-C(105)	122.7(10)
C(102)-C(103)-C(105)	119.1(9)
C(103)-C(104)-N(102)	122.1(10)
O(103)-C(106)-N(102)	110.3(7)
O(103)-C(106)-C(107)	104.7(7)
N(102)-C(106)-C(107)	114.4(8)
C(108)-C(107)-C(106)	102.9(8)
O(105)-C(108)-C(107)	110.0(8)
O(105)-C(108)-C(109)	113.6(8)
C(107)-C(108)-C(109)	113.4(8)
O(105)-C(108)-C(112)	110.3(8)
C(107)-C(108)-C(112)	101.7(8)
C(109)-C(108)-C(112)	107.2(8)
C(110)-C(109)-C(108)	104.0(9)
C(111)-C(110)-C(109)	109.3(9)
C(111)-C(110)-C(113)	59.6(7)
C(109)-C(110)-C(113)	116.5(10)
O(104)-C(111)-C(110)	121.6(9)
O(104)-C(111)-C(113)	120.4(9)
C(110)-C(111)-C(113)	61.2(8)
O(104)-C(111)-C(112)	117.3(9)
C(110)-C(111)-C(112)	108.5(8)
C(113)-C(111)-C(112)	115.4(9)
C(111)-C(113)-C(110)	59.2(7)
O(201)-C(201)-N(202)	124.6(8)

O(201)-C(201)-N(201)	122.2(9)
N(202)-C(201)-N(201)	113.2(9)
O(202)-C(202)-N(201)	118.6(9)
O(202)-C(202)-C(203)	126.3(9)
N(201)-C(202)-C(203)	115.1(8)
C(204)-C(203)-C(202)	118.1(9)
C(204)-C(203)-C(205)	123.8(9)
C(202)-C(203)-C(205)	118.1(8)
C(203)-C(204)-N(202)	122.5(9)
O(203)-C(206)-N(202)	107.4(7)
O(203)-C(206)-C(207)	106.1(7)
N(202)-C(206)-C(207)	115.9(8)
C(206)-C(207)-C(208)	105.1(7)
C(210)-C(209)-C(208)	106.9(8)
C(211)-C(210)-C(213)	59.3(7)
C(211)-C(210)-C(209)	108.4(9)
C(213)-C(210)-C(209)	118.2(10)
O(205)-C(211)-C(213)	118.8(8)
O(205)-C(211)-C(212)	119.4(8)
C(213)-C(211)-C(212)	113.3(8)
O(205)-C(211)-C(210)	122.8(9)
C(213)-C(211)-C(210)	60.5(7)
C(212)-C(211)-C(210)	108.3(8)
O(203)-C(212)-C(211)	110.8(7)
O(203)-C(212)-C(208)	105.5(7)
C(211)-C(212)-C(208)	107.5(8)
C(211)-C(213)-C(210)	60.2(7)
O(204)-C(208)-C(209)	106.6(8)
O(204)-C(208)-C(207)	114.8(8)
C(209)-C(208)-C(207)	113.3(8)
O(204)-C(208)-C(212)	113.4(7)
C(209)-C(208)-C(212)	104.8(7)
C(207)-C(208)-C(212)	103.7(7)
O(103)-C(112)-C(111)	112.7(8)
O(103)-C(112)-C(108)	105.7(7)
C(111)-C(112)-C(108)	102.5(8)
C(102)-N(101)-C(101)	126.6(9)
C(101)-N(102)-C(104)	122.1(8)

C(101)-N(102)-C(106)	120.3(8)
C(104)-N(102)-C(106)	117.3(8)
C(202)-N(201)-C(201)	127.5(9)
C(204)-N(202)-C(201)	123.4(8)
C(204)-N(202)-C(206)	118.4(8)
C(201)-N(202)-C(206)	117.8(8)
C(106)-O(103)-C(112)	111.1(7)
C(206)-O(203)-C(212)	107.2(7)

Symmetry transformations used to generate equivalent atoms:

Table S22. Anisotropic displacement parameters ($Å^2$x 10^3)for **tc-T**. The anisotropic displacement factor exponent takes the form: $-2\pi^2[h^2a^{*2}U^{11} + ... + 2 h k a^* b^* U^{12}]$

	U^{11}	U^{22}	U^{33}	U^{23}	U^{13}	U^{12}
C(101)	27(6)	25(6)	25(5)	-4(5)	-5(4)	0(5)
C(102)	20(5)	22(5)	26(5)	7(5)	0(4)	-16(5)
C(103)	15(5)	31(6)	29(5)	1(5)	-3(4)	2(5)
C(104)	14(5)	27(6)	27(5)	3(5)	1(4)	-8(5)
C(105)	36(6)	21(6)	32(6)	-3(5)	-6(5)	6(5)
C(106)	20(5)	37(6)	9(4)	-6(4)	-4(4)	7(5)
C(107)	14(5)	36(6)	12(4)	11(4)	-2(4)	0(5)
C(108)	17(5)	16(5)	16(5)	3(4)	6(4)	2(4)
C(109)	33(6)	27(6)	25(5)	-6(5)	-5(5)	-3(5)
C(110)	35(6)	21(6)	45(7)	-2(5)	8(5)	-6(5)
C(111)	27(6)	26(6)	23(5)	0(4)	1(4)	7(5)
C(113)	40(7)	35(6)	31(6)	-18(5)	1(5)	1(6)
C(201)	21(5)	24(6)	20(5)	-3(4)	6(4)	3(5)
C(202)	12(5)	29(6)	14(5)	10(4)	3(4)	1(4)
C(203)	16(5)	12(5)	27(5)	3(4)	3(4)	-2(4)
C(204)	23(5)	20(5)	22(5)	1(4)	1(4)	1(5)
C(205)	29(6)	25(6)	33(6)	5(5)	8(5)	-4(5)
C(206)	14(5)	19(5)	17(5)	1(4)	3(4)	0(4)
C(207)	20(5)	23(5)	20(5)	1(4)	2(4)	10(5)
C(209)	27(6)	19(5)	32(6)	-8(4)	4(5)	6(5)
C(210)	30(6)	27(6)	35(6)	-3(5)	13(5)	-8(5)

C(211)	17(5)	32(6)	16(5)	6(4)	0(4)	2(5)
C(212)	22(5)	17(5)	15(4)	-1(4)	10(4)	2(4)
C(213)	36(6)	39(7)	24(6)	-10(5)	8(5)	-6(6)
C(208)	11(5)	19(5)	18(5)	5(4)	3(4)	2(4)
C(112)	10(5)	24(5)	19(5)	-7(4)	-4(4)	-8(4)
N(101)	28(5)	23(4)	20(4)	-3(4)	-1(4)	-7(4)
N(102)	21(4)	26(5)	20(4)	2(4)	-6(3)	4(4)
N(201)	25(5)	22(4)	16(4)	-5(3)	6(3)	-1(4)
N(202)	16(4)	14(4)	17(4)	-1(3)	-1(3)	-3(4)
O(101)	48(5)	39(5)	32(4)	-8(4)	-4(4)	12(5)
O(102)	28(4)	35(4)	25(4)	9(3)	-3(3)	-8(4)
O(103)	19(3)	28(4)	16(3)	0(3)	0(3)	-5(3)
O(104)	23(4)	43(5)	33(4)	0(4)	1(3)	20(4)
O(105)	19(4)	29(4)	23(3)	7(3)	3(3)	4(3)
O(201)	38(4)	27(4)	29(4)	-12(3)	14(3)	-14(4)
O(202)	25(4)	28(4)	23(4)	-5(3)	10(3)	-4(3)
O(203)	17(3)	23(3)	16(3)	1(3)	0(3)	6(3)
O(204)	20(4)	25(4)	30(4)	-2(3)	12(3)	-4(3)
O(205)	24(4)	40(5)	28(4)	-7(3)	3(3)	12(4)

Oligonucleotide Synthesis.

Syntheses of oligonucleotides were performed on the 1.3 µmol scale of a DNA synthesizer using standard solid-phase phosphoramidite chemistry. Oligomers were assembled using the manufacturer's protocols on nucleoside preloaded dA-CPG 500 (48 µmol/g). Natural phosphoramidites (dT, dC4Bz, dA6Bz, dG2dmf) were coupled as a 0.1 M solution in CH_3CN, iso-tricyclo- and bicyclophosphoramidites **10** and **13** as 0.15 M solution in CH_3CN. The coupling step was 90 s for natural phosphoramidites. An extended coupling step of 7 min for amidites **10** and **13** and 12 min for the **tc-T** phosphoramidite was necessary to achieve >95% coupling efficiency (trityl assay). As a coupling reagent, 5-(ethylthio)-1*H*-tetrazole (0.25 M in CH_3CN) was used. Capping was performed with a solution of DMAP (0.5 M in CH_3CN, Cap A) and a solution of 25% Ac_2O and 12.5% sym-collidine in CH_3CN (Cap B). Oxidation was performed with a solution of 20 mM I_2 and 0.45 M sym-collidine in 2.1:1 CH_3CN/H_2O. In order to achieve the full length sequence of oligonucleotide **ON4**, oxidation was performed using 1.1 M *tert*-butyl hydroperoxide in CH_2Cl_2 (0.8 min). Detritylation after coupling of phosphoramidites was carried out using a solution of 3% dichloroacetic acid in dichloroethane.

Oligonucleotide Deprotection and Purification.

Deprotection of the oligonucleotides and detachment from the solid support was carried out using standard conditions (concd aq NH_3 for 16 h at 55 °C). The solutions were centrifuged after deprotection, the supernatants were collected and the remaining beads washed with 2×0.5 mL of H_2O. The combined supernatants were then concentrated to dryness. Crude oligomers were purified by ion-exchange HPLC (Dionex DNA Pac-PA200 or DNA Pac-PA100). As mobile phases, the following buffers were prepared: (A) 25 mM Trizma (2-amino-2-hydroxymethyl-1,3-propanediol) in H_2O, pH 8.0; (B) 25 mM Trizma, 1.25 M NaCl in H_2O, pH 8.0. Linear gradients of B in A were used (typically 0 to 50% B in A over 30 min), with a 1 mL/min flow rate and detection at 260 nm. Purified oligonucleotides were desalted over Sep-Pak cartridges, quantified at 260 nm with a Nanodrop spectrophotometer, and analyzed by ESI^- mass spectrometry. Oligonucleotides **ON6** and **ON7**, which were synthesized as DMT-on, were after abovementioned purification and desalting lyophilized and detritytlated by treatment with 300 µL 80% AcOH for 45 min at RT. These solutions were subsequently diluted with 300 µL of H_2O and extracted with Et_2O (3×300 µL). Aqueous layer was further diafiltered (Centrifugal Filters Amicon Ultra 0.5 mL, Ultracel – 3K, MWCO 3 kDa) against water until neutral pH. All solutions of oligonucleotides in H_2O were then stored at −18 °C.

UV−Melting Curves.

UV−melting curves were recorded on a Varian Cary Bio100 UV/vis spectrophotometer. Absorbances were monitored at 260 nm, and the heating rate was set to 0.5 °C/min. A cooling−heating−cooling cycle in the temperature range 20−70 °C was applied. T_m values were obtained from the maximum of the first derivative curves and reported as the average of at least three ramps (±1 °C error). All measurements were carried out in NaCl (150 mM), NaH_2PO_4 (10 mM) buffer at pH 7.0 with a duplex concentration of 1.2 µM.

Table S23. ESI-MS and HPLC characterization of modified oligonucleotides **ON1-7**

Entry	m/z calc	m/z found	t_r (min)	Conditions	HPLC-purity
**ON1**	3700.4	3700.6	22.6	Buffer A: 25 mM Trizma in H_2O, pH 8<br>Buffer B: 25 mM Trizma, 1.25 M NaCl in H_2O, pH 8<br>Dionex DNA-Pac PA200, 0-50 % B in 30 min	87%
**ON2**	3724.5	3724.4	22.6		90%
**ON3**	3724.5	3724.4	22.4		93%
**ON4**	3714.5	3714.6	22.4		89%
**ON5**	3714.5	3714.6	22.5	Buffer A: 10 mM NaOH in H_2O, pH 12<br>Buffre B: 10 mM NaOH, 1.5 M NaCl in H_2O, pH 12<br>Dionex DNA-Pac PA200, 0-50 % B in 30 min	---
**ON6**	3752.5	3752.6	22.1		---
**ON7**	3752.5	3752.7	22.1		---

ON1

Acq. File: Dugovic GSE 1-15 min.wiff

Electrospray Low Resolution

Negative Ion Mode

ACN / H2O (1:1) + 1% TEA

ON1

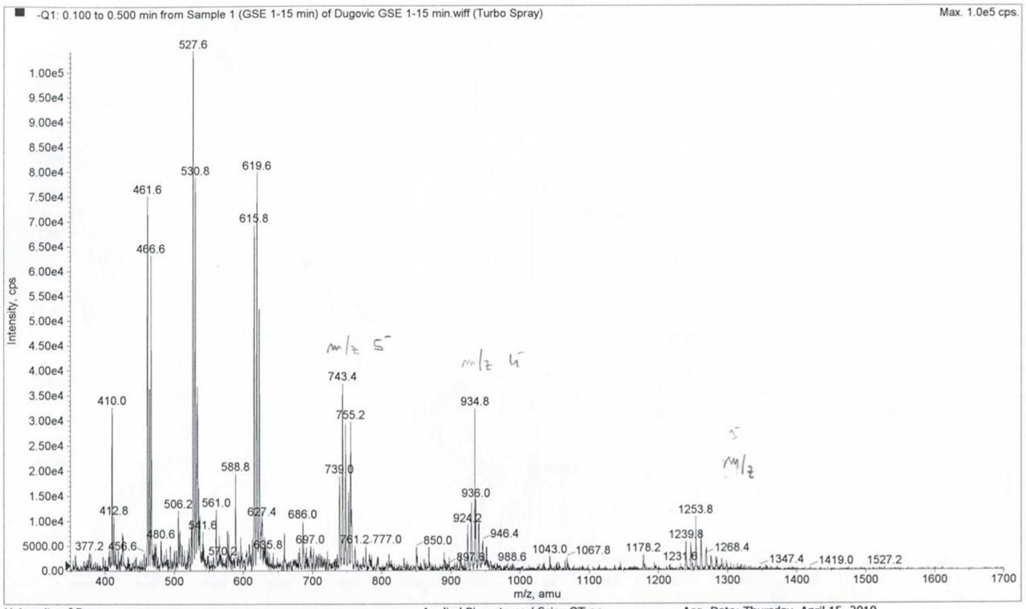

University of Bern

Department of Chemistry and Biochemistry

Applied Biosystems / Sciex QTrap

Acq. Date: Thursday, April 15, 2010

Acq. Time: 11:35

Acq. File: n/a

Electrospray Low Resolution

Negative Ion Mode

ACN / H2O (1:1) + 1% TEA

Dataset Name: -Q1: 0.100 to 0.500 min from Sample 1 (GSE 1-15 min) of Dugovic GSE 1-15 min.wiff (Turbo Spray)

Analysis Results

#	Mass (avg.)	m/z	z	Area	+/-	Apex Mass	Area	Evidence
1	3700.6046					3700.6581	1506015.8…	C
		410.1504	-9	152737.78…	-0.0205			
		461.5606	-8	346733.53…	-0.0075			
		527.6580	-7	503227.12…	0.0076			
		615.7912	-6	358442.03…	0.0312			
		739.0614	-5	96918.9297	-0.0521			
		924.0405	-4	47956.4375	-0.1032			
2	3722.7711					3722.8630	1208203.2…	C
		412.7083	-9	52692.9766	0.0745			
		464.3528	-8	155716.04…	0.0139			
		530.8301	-7	386223.18…	0.0131			
		619.4428	-6	341376.34…	-0.0116			
		743.5231	-5	195747.26…	-0.0237			
		929.5511	-4	76447.4375	-0.1343			
3	3743.7892					3744.2500	829107.26…	C
		415.0649	-9	17200.1426	0.0957			
		466.8518	-8	170048.98…	-0.1144			
		533.8320	-7	206255.82…	0.0124			
		623.0342	-6	212756.96…	0.0768			
		747.8260	-5	132430.01…	0.0756			
		934.8704	-4	90415.3281	-0.0695			

University of Bern

Department of Chemistry and Biochemistry

Applied Biosystems / Sciex QTrap

Acq. Date: n/a

Acq. Time: n/a

ON2

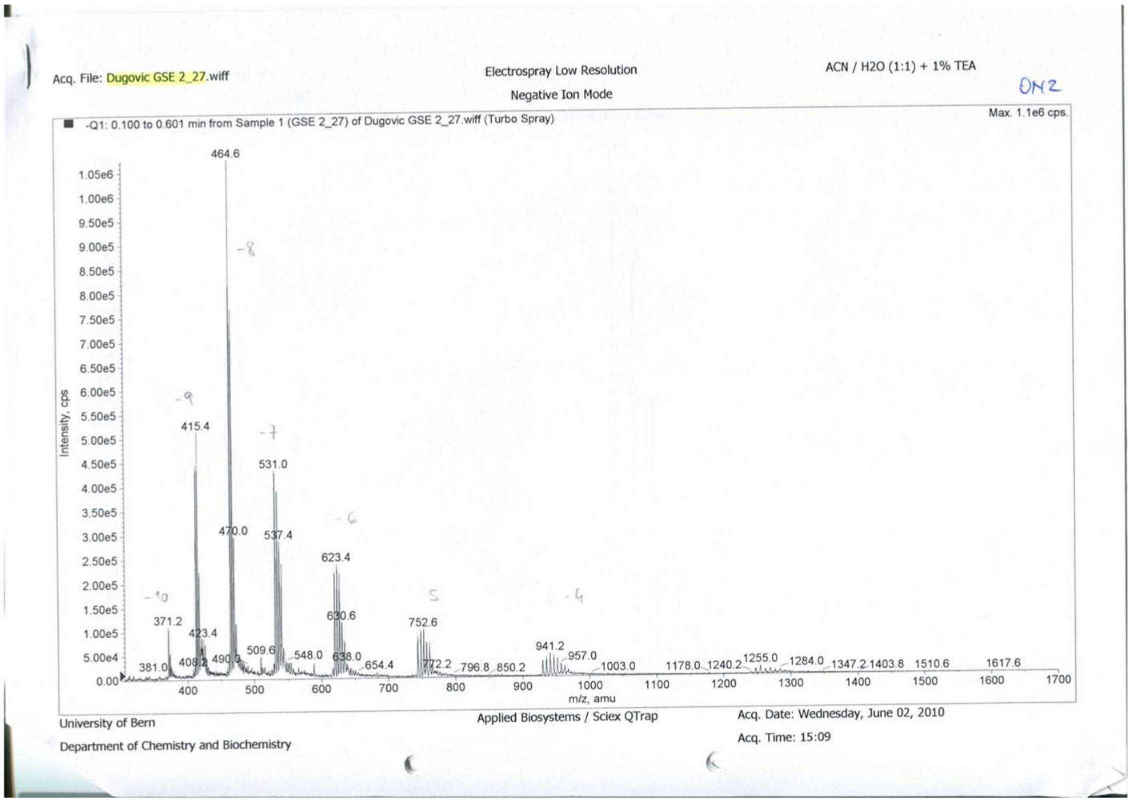

Acq. File: n/a

Electrospray Low Resolution

Negative Ion Mode

ACN / H2O (1:1) + 1% TEA

Dataset Name: -Q1: 0.100 to 0.601 min from Sample 1 (GSE 2_27) of Dugovic GSE 2_27.wiff (Turbo Spray)

Analysis Results

#	Mass (avg.)	m/z	z	Area	+/-	Apex Mass	Area	Evidence
1	3724.4116					3724.3495	11181023....	C
		371.4135	-10	406437.68...	-0.0202			
		412.8394	-9	2212810.5...	0.0233			
		464.5363	-8	4589628.5...	-0.0078			
		531.0537	-7	2231224.5...	0.0023			
		619.7095	-6	1098304.3...	-0.0183			
		743.9055	-5	461537.78...	0.0306			
		929.9800	-4	181080.39...	-0.1155			
2	3746.4456					3746.3282	9514602.0...	C
		373.6485	-10	191537.70...	0.0113			
		415.2745	-9	1808198.2...	0.0102			
		467.2836	-8	3582552.5...	-0.0147			
		534.2224	-7	1876631.8...	0.0233			
		623.4140	-6	1249058.1...	0.0138			
		748.2537	-5	592186.93...	-0.0280			
		935.4959	-4	214436.65...	-0.1081			
3	3767.8742					3767.8558	5898605.2...	C
		375.5768	-10	114225.94...	-0.2032			
		417.5914	-9	897905.68...	-0.0539			
		469.9745	-8	1503313.0...	-0.0023			
		537.2980	-7	1465355.6...	0.0377			
		627.0160	-6	1087575.2...	0.0444			
		752.5794	-5	539404.50...	0.0120			
		940.9495	-4	290825.28...	-0.0116			

University of Bern

Department of Chemistry and Biochemistry

Applied Biosystems / Sciex QTrap

Acq. Date: n/a

Acq. Time: n/a

ON3

Acq. File: Dugovic GSE 3_27.wiff | Electrospray Low Resolution | ACN / H2O (1:1) + 1% TEA

Negative Ion Mode

ON 3

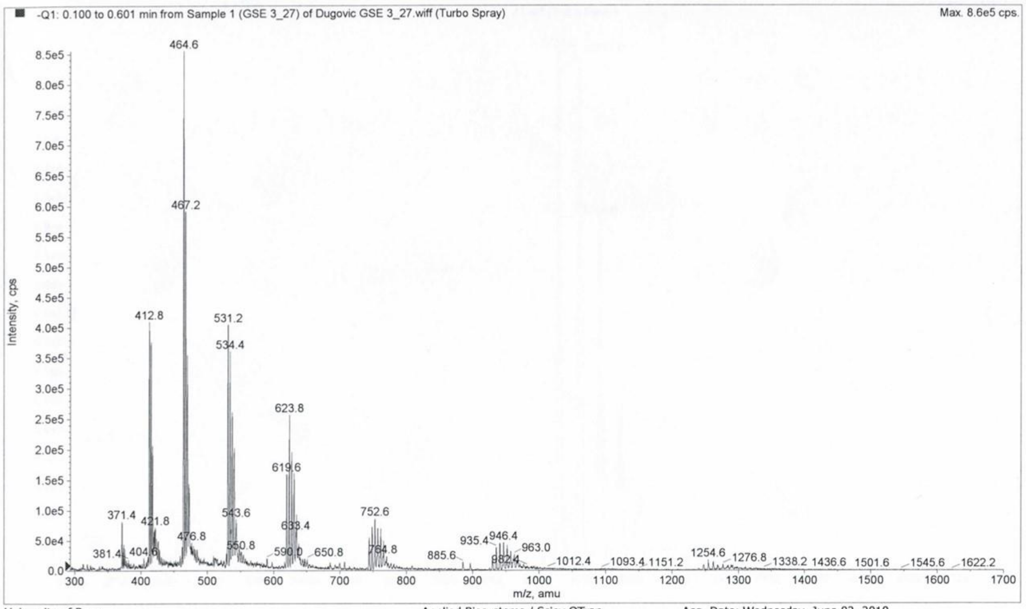

University of Bern | Applied Biosystems / Sciex QTrap | Acq. Date: Wednesday, June 02, 2010

Department of Chemistry and Biochemistry | Acq. Time: 15:24

Acq. File: n/a | Electrospray Low Resolution | ACN / H2O (1:1) + 1% TEA

Negative Ion Mode

Dataset Name: -Q1: 0.100 to 0.601 min from Sample 1 (GSE 3_27) of Dugovic GSE 3_27.wiff (Turbo Spray)

Analysis Results

#	Mass (avg.)	m/z	z	Area	+/-	Apex Mass	Area	Evidence
1	3724.3984					3724.4112	9558867.5...	C
		371.4176	-10	335889.96...	-0.0148			
		412.8089	-9	2008751.2...	-0.0057			
		464.5440	-8	3986916.7...	0.0016			
		531.0667	-7	1977006.6...	0.0172			
		619.7244	-6	871151.06...	-0.0012			
		743.8578	-5	261677.60...	-0.0145			
		929.8568	-4	117474.26...	-0.2354			
2	3746.5299					3746.2269	8342563.0...	C
		373.7011	-10	167928.26...	0.0556			
		415.3039	-9	1661423.0...	0.0302			
		467.2709	-8	2957939.5...	-0.0379			
		534.2522	-7	1753576.5...	0.0411			
		623.4203	-6	1134682.3...	0.0061			
		748.2772	-5	433132.84...	-0.0213			
		935.4664	-4	233880.57...	-0.1586			
3	3767.4685					3767.4625	6412650.4...	C
		375.5595	-10	108821.96...	-0.1799			
		417.5286	-9	1066407.6...	-0.0716			
		469.9254	-8	2046674.8...	-0.0008			
		537.2310	-7	1469329.2...	0.0287			
		626.9644	-6	958665.62...	0.0604			
		752.5690	-5	497113.34...	0.0827			
		940.9045	-4	265637.78...	0.0448			

University of Bern | Applied Biosystems / Sciex QTrap | Acq. Date: n/a

Department of Chemistry and Biochemistry | Acq. Time: n/a

ON4

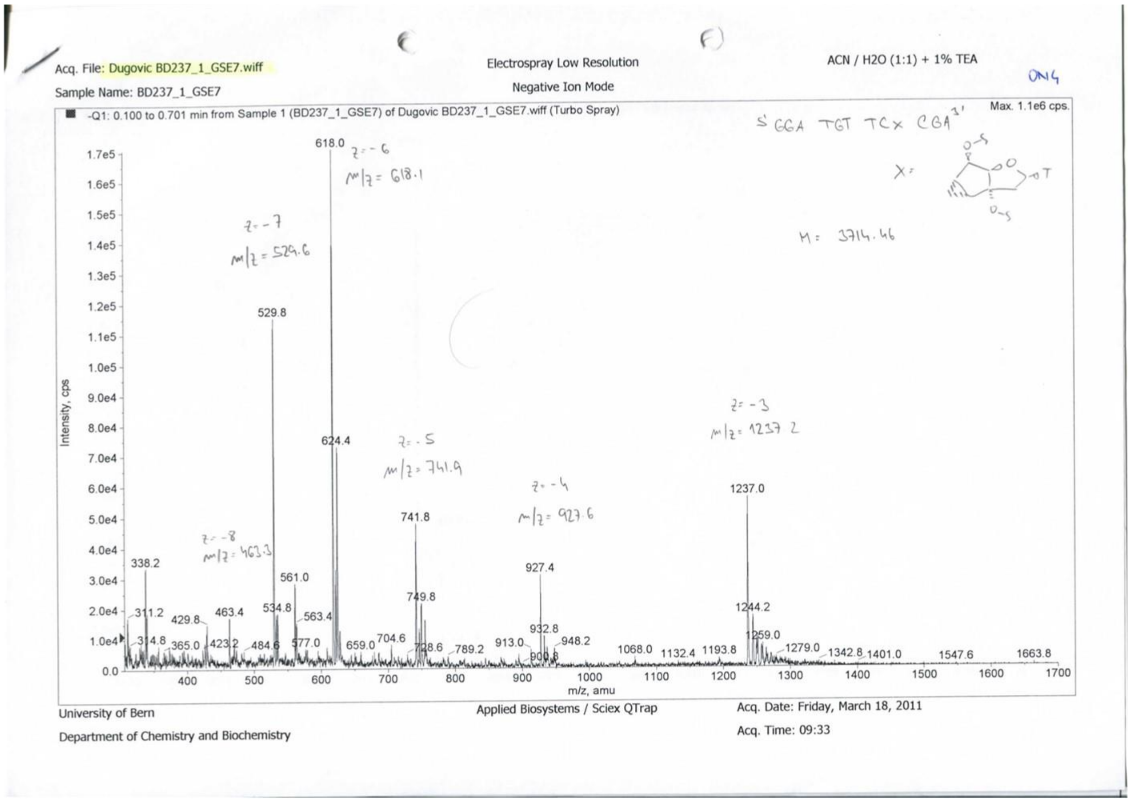

Acq. File: n/a

Sample Name: n/a

Electrospray Low Resolution

Negative Ion Mode

ACN / H2O (1:1) + 1% TEA

Dataset Name: -Q1: 0.100 to 0.701 min from Sample 1 (BD237_1_GSE7) of Dugovic BD237_1_GSE7.wiff (Turbo Spray)

Analysis Results

#	Mass (avg.)	m/z	z	Area	+/-	Apex Mass	Area	Evidence
1	3714.5049					3714.4507	1669831.1...	C
		529.6863	-7	531963.37...	0.0502			
		618.0677	-6	776530.56...	-0.0090			
		741.8129	-5	216861.75...	-0.0807			
		927.5195	-4	144475.43...	-0.0993			
2	3751.8730					3751.9331	485002.60...	C
		534.9659	-7	70487.8984	-0.0085			
		624.3148	-6	269635.43...	0.0100			
		749.3767	-5	102372.42...	0.0095			
		936.8615	-4	42506.8359	-0.0993			
3	3736.5180					3736.7343	341014.32...	C
		532.8142	-7	73807.8594	0.0333			
		621.7816	-6	158940.18...	0.0360			
		746.2697	-5	53899.8672	-0.0265			
		932.9177	-4	54366.4063	-0.2044			

University of Bern

Department of Chemistry and Biochemistry

Applied Biosystems / Sciex QTrap

Acq. Date: n/a

Acq. Time: n/a
